# Organocopper cross-coupling reaction for C–C bond formation on highly sterically hindered structures†
†Electronic supplementary information (ESI) available. CCDC 1566502. For ESI and crystallographic data in CIF or other electronic format see DOI: 10.1039/c9sc00891h


**DOI:** 10.1039/c9sc00891h

**Published:** 2019-05-10

**Authors:** Miku Oi, Ryo Takita, Junichiro Kanazawa, Atsuya Muranaka, Chao Wang, Masanobu Uchiyama

**Affiliations:** a Graduate School of Pharmaceutical Sciences , University of Tokyo , Hongo 7-3-1, Bunkyo-ku , Tokyo , Japan . Email: takita@mol.f.u-tokyo.ac.jp ; Email: uchiyama@mol.f.u-tokyo.ac.jp; b Advanced Elements Chemistry Research Team , RIKEN Center for Sustainable Resource Science , Elements Chemistry Laboratory , RIKEN , Wako-shi , Saitama 351-0198 , Japan

## Abstract

We describe a powerful, broadly applicable cross-coupling protocol that enables carbon–carbon bond formation at highly sterically hindered carbon centers (both sp^2^ and sp^3^) by employing organocopper reagents under palladium catalysis. Experimental studies and theoretical calculations indicated that the key to the unique reactivity of copper is the relatively low activation energy of the compact transmetalation transition state, due to Cu(i)–Pd(ii) interaction, which is associated with small values of deformation energy of the reactants. This reaction is applicable to a variety of bulky substrates, including compounds inert to previous cross-coupling chemistry and has high functional group tolerance.

## Abstract 2

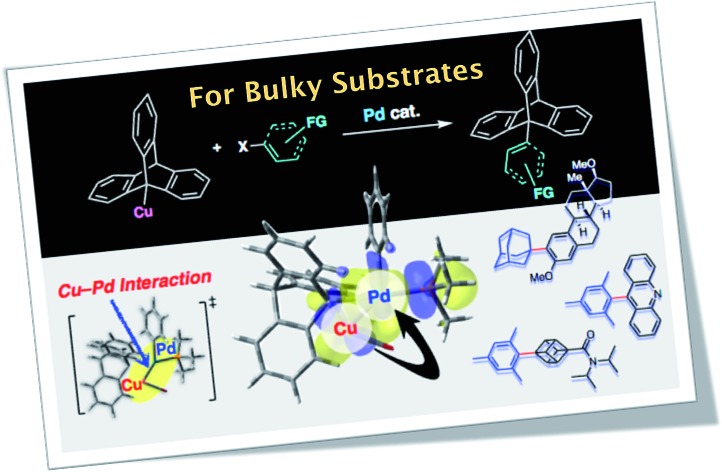
A potent cross-coupling methodology that enables efficient carbon–carbon bond formation at sterically hindered sp^2^- and sp^3^-carbons has been developed.

## Introduction

**Scheme 1:**
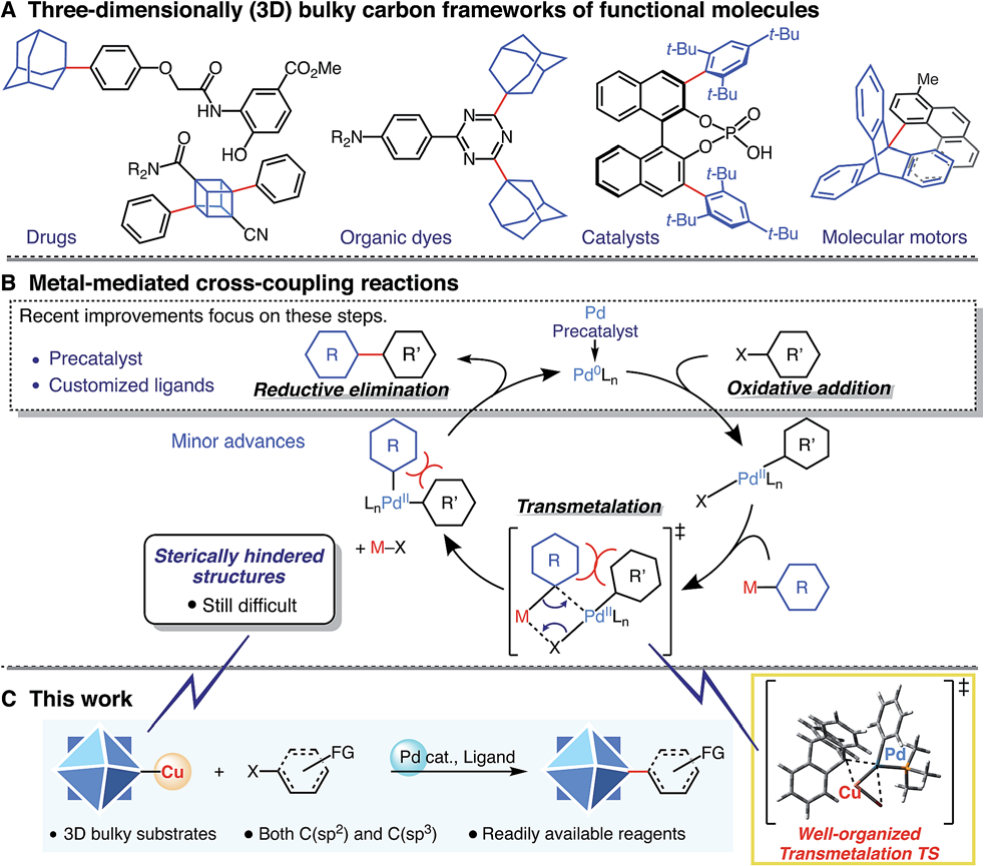
(A) Three-dimensionally (3D) bulky carbon frameworks in a broad range of disciplines. (B) Fundamental steps in cross-coupling reactions and recent developments in the catalytic cycle. (C) Pd-catalyzed organocopper cross-coupling reaction on highly sterically hindered structures (this work).

Three-dimensionally (3D) bulky carbon frameworks and other bulky substrates have become important scaffolds for a broad range of functional molecules (Scheme 1A). They are useful in many fields due to features such as improved solubility, enhanced stability, and increased control of molecular stacking.^1^–^4^ While transition metal-catalyzed cross-coupling reactions are among the most developed of C–C bond-forming reactions,^5^ bond formation at sterically hindered structures remains a challenging task in cross-coupling chemistry, even with sp^2^- or sp^3^-carbon substrates. Recent improvements have focused mostly on the design of (pre)catalysts and customized ligands in order to achieve efficient generation of active palladium species, and on the oxidative addition/reductive elimination step in the catalytic cycle (Scheme 1B).^6^ These “state-of-the-art” systems, in particular with customized ligands, enable bond formation on sterically hindered substrates.^7^ However, transmetalation, a fundamental step in cross-coupling reactions,^8^ can also be targeted to address this challenge.

Herein, we report a powerful and broadly applicable (to both sp^2^- and sp^3^-carbons) Pd-catalyzed cross-coupling reaction of organocopper reagents that enables efficient C–C bond formation even on 3D bulky carbon frameworks (Scheme 1C). Notably, this reaction proceeds under mild conditions using readily available reagents and has high functional group tolerance. Experimental and theoretical studies revealed that copper(i)–palladium(ii) interaction facilitates the formation of a compact and well-organized transition state in the transmetalation step.

## Results & discussion

We commenced our search to develop a potent methodology by focusing on the triptycene framework,^9^ due to its extremely hindered *tertiary* sp^3^-carbon at the bridgehead 9-position and unprecedent use in cross-coupling chemistry. The cross-coupling reactions using a boronate ester or zinc(ii) complex of 9-triptycene (**1a** and **1b**) failed to afford any coupled product under the representative palladium-catalyzed conditions (runs 1–3, Table 1).7e,g,h On the other hand, we found that the cross-coupling reaction of 9-triptycenylcopper(i) complex **1c**^10^ with **2** smoothly proceeded using 5 mol% of Pd(OAc)_2_ and tris(*o*-methoxyphenyl)phosphine (**L1**) at 80 °C to afford the coupling product **3** in 86% yield (run 4). The direct use of organocopper reagents in Pd-catalyzed cross-coupling reactions is limited,^11^ and a copper co-catalyst^12^ as well as relay catalysis using palladium and copper^13^ have been employed. The absence of a palladium catalyst yielded no desired product (run 5). Under similar reaction conditions, other organometallic reagents (**1a**, **1b**, **1d**, and **1e**) were also not competent (runs 6–9).

**Table 1:** Screening of 9-metalated triptycene derivatives

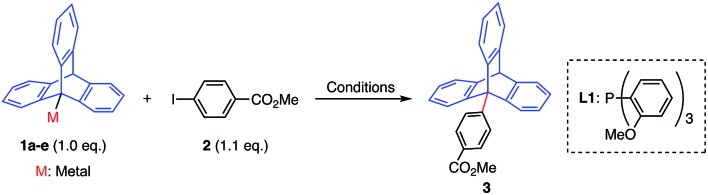			
Run	Metal	Conditions, time (h)	Yield^*a*^ (%)
1	Bpin (**1a**)	Pd(OAc)_2_, SPhos, K_3_PO_4_, toluene, 100 °C^*b*^	0
2	ZnCl (**1b**)	Pd(OAc)_2_, RuPhos, THF–toluene, 100 °C^*c*^	0
3	ZnCl (**1b**)	XPhos G3, XPhos, THF–toluene, 100 °C^*d*^	0
4	Cu (**1c**)	Pd(OAc)_2_, **L1**^*e*^, THF–toluene, 80 °C	86
5	Cu (**1c**)	**L1** ^*e*^, THF–toluene, 80 °C	0
6	Bpin (**1a**)	Pd(OAc)_2_, **L1**^*e*^, K_3_PO_4_, THF–toluene, 100 °C	0
7	ZnCl (**1b**)	Pd(OAc)_2_, **L1**^*e*^, THF–toluene, 100 °C	Trace
8	MgCl (**1d**)	Pd(OAc)_2_, **L1**^*e*^, THF–toluene, 100 °C	0
9	Li (**1e**)	Pd(OAc)_2_, **L1**^*e*^, THF–toluene, 100 °C	0

^*a*^NMR yields determined using dimethylsulfone as an internal standard. Reaction time: 4 h (runs 4 and 5) or 20 h (other runs).

^*b*^ Ref. 7*e*.

^*c*^ Ref. 7*g*.

^*d*^ Ref. 7*h*.

^*e*^ **L1**: tris(*o*-methoxyphenyl)phosphine.

**Scheme 2:**
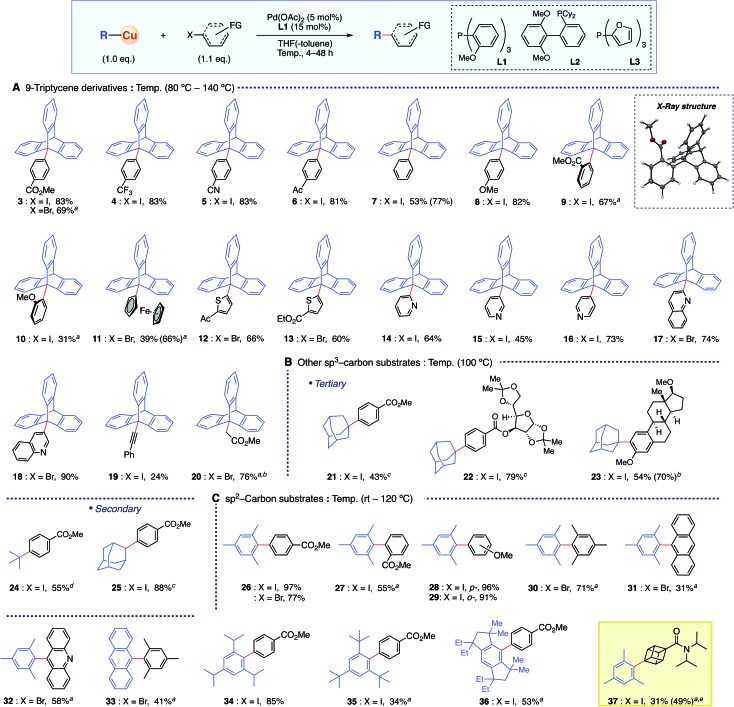
Organocopper cross-coupling reaction of sp^3^- and sp^2^-carbon substrates with sterically hindered structures. Isolated yields are shown (^1^H NMR yields in parentheses). ^*a*^**L2** was used instead of **L1**. ^*b*^ Ni(acac)_2_ was used instead of Pd(OAc)_2_. ^*c*^**L2** was used instead of **L1** and 3 eq. of TMEDA were added. ^*d*^**L3** was used instead of **L1** at –20 °C and 3 eq. of TMEDA were added. ^*e*^ 10 mol% Pd catalyst and 30 mol% **L2** were used.

The optimized conditions of the present organocopper cross-coupling reaction were then applied to bond formation using a variety of electrophiles (Scheme 2A). Both electron-withdrawing and -donating groups on aryl halides, containing CO_2_Me, CF_3_, CN, acetyl, and MeO groups, were compatible with the reaction conditions, and the desired products were obtained in good yields (**3–8**). Moreover, sterically demanding *ortho*-substituted substrates gave 9-arylated triptycene derivatives **9** and **10**. X-ray crystallography of **9** confirmed the desired C–C bond formation. A straightforward, one-step synthesis of 9-ferrocenyltriptycene **11** (ref. 4*b*) was accomplished with the present coupling strategy. Various heteroaromatic moieties were successfully coupled (**12–18**), and bond formation was also achieved with sp-carbon of an alkynyl substrate **19**. The coupling reaction with ethyl bromoacetate proceeded to give the product **20** in 76% yield having a C(sp^3^)–C(sp^3^) bond.^14^ Since existing syntheses of 9-functionalized triptycenes are rather lengthy,^9^ the present methodology offers substantial synthetic advantages, including high functional group tolerance.

Other sterically hindered *tertiary* sp^3^-carbon compounds were also competent substrates for this reaction (Scheme 2B). The adamantyl group was successfully introduced not only onto a simple aryl group (**21**), but also onto aryl halide moieties on a sugar skeleton and into a steroid backbone, affording the corresponding products **22** and **23** in good yields. These results indicate that the present methodology would be suitable for late-stage functionalization of biologically relevant compounds and functional molecules with bulky *tertiary* alkyl moieties. Similarly, the introduction of a *tert*-butyl group having β-hydrogen was achieved, affording **24** in 63% yield. A *secondary* 2-adamantyl group was also introduced onto an aromatic ring to give **25** in 88% yield.

Further examination revealed that this methodology was also applicable to sterically hindered sp^2^-carbon using conditions essentially identical to those employed for sp^3^-substrates (Scheme 2C). The reaction between mesitylcopper(i) and **2** proceeded efficiently at room temperature, affording the coupled product **26** in quantitative yield. The same organocopper(i) reagent reacted smoothly under these conditions with various electrophiles, affording the corresponding biaryl products **27–32** in good yields, including hindered *ortho*-substituted substrates. Furthermore, the present protocol realized the reactions with much bulkier copper reagents, such as “super-mesityl”^15^ (**35**) and EMind^16^ (**36**) substrates. Importantly, this reaction could also be applied to the cubane skeleton (*i.e.***37**). Although the cubane motif has recently attracted great attention as a bioisostere of benzene in pharmaceuticals,^17^ there have only been two examples of cross-coupling chemistry, and the yields were not high with respect to the catalyst loading.^18^ In contrast, the present organocopper cross-coupling reaction enabled catalytic C–C bond formation with an iodocubane derivative for the first time, installing the sterically hindered mesityl group in 49% yield using a 10 mol% Pd catalyst.^14^

**Fig. 1:**
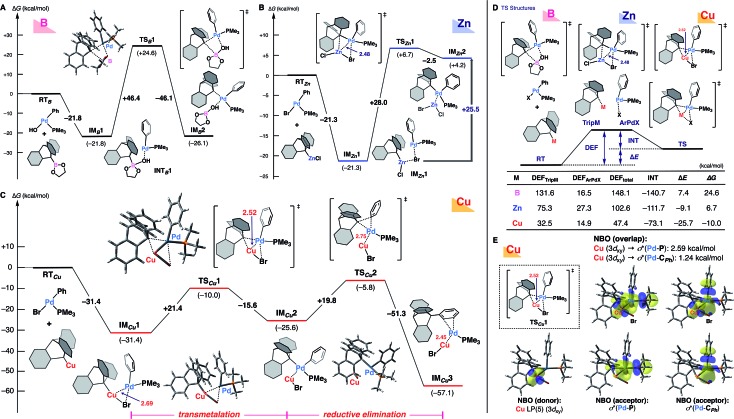
Theoretical calculations for the transmetalation step between an arylpalladium complex and 9-metalated triptycene complexes (A) with a 9-triptycenylboronate reagent, (B) with a 9-triptycenylzinc chloride reagent, and (C) with a 9-triptycenylcopper reagent (the reductive elimination step is also shown). Energy changes and bond lengths at the *ω*B97X-D/SDD (for Pd, Cu, Zn, and Br) & 6-31+G* (for other atoms) level of theory are shown in kcal mol^–1^ and Å, respectively. (D) Results of EDA. (E) Results of NBO analysis.

Given that the nature of the metal species should have the greatest influence on the transmetalation step (Scheme 1B), and taking into account the mechanistic implications of experimental findings,^19^ we performed theoretical calculations for the ligand transfer process from 9-metalated triptycene (boron, zinc, and copper) to an arylpalladium complex as a model of the transmetalation step (Fig. 1) at the *ω*B97X-D/SDD & 6-31+G* level of theory. DFT calculation for the reaction of 9-triptycenylboronate with *trans*-Pd(Ph)OH(PMe_3_)^20^ indicated that the bulky triptycene group distorts the transition-state structure (**TS*_B_*1**), resulting in a high activation energy (Δ*G*^‡^ = +46.4 kcal mol^–1^, Fig. 1A). Although a lower activation barrier (Δ*G*^‡^ = +28.0 kcal mol^–1^) was observed with 9-triptycenylzinc chloride *via***TS*_Zn_*1**, this reaction (**IM*_Zn_*1**–**TS*_Zn_*1**–**IM*_Zn_*2**) is thermodynamically unfavorable; the transmetalated product **IM*_Zn_*2** is quite unstable (+25.5 kcal mol^–1^, compared with **IM*_Zn_*1**), suggesting that efficient transmetalation is improbable (Fig. 1B). These findings are consistent with the experimental results shown in Table 1.

The situation with the copper reagent is completely different; the initial coordination of 9-triptycenylcopper and *trans*-Pd(Ph)Br(PMe_3_) (**RT*_Cu_***) affords **IM*_Cu_*1** with a large stabilization energy (–31.4 kcal mol^–1^) (Fig. 1C). Furthermore, the distance between the copper and palladium atoms is shorter (2.69 Å) than the sum of their van der Waals radii (3.03 Å), supporting the existence of strong metal–metal interaction.^21^ This Cu–Pd interaction results in the formation of a compact transition-state structure (**TS*_Cu_*1**, Cu–Pd: 2.52 Å) that facilitates delivery of the bulky triptycenyl group from the copper to the palladium center with a reasonable activation energy (Δ*G*^‡^ = +21.4 kcal mol^–1^). The resultant **IM*_Cu_*2** having both phenyl and triptycenyl ligands on palladium in a *cis*-fashion then undergoes reductive elimination. The reductive elimination also reasonably proceeds (Δ*G*^‡^ = +19.8 kcal mol^–1^) to achieve the C–C bond formation on the triptycene framework (**IM*_Cu_*3**).

Thus, the unique observed reactivity of the copper complex, in contrast to the boron or zinc complex, is supported by the theoretical calculations. Although the possibility of a similar metal–metal interaction in transmetalation has been suggested previously,^22^–^24^ our DFT calculations directly compare the transmetalation transition-state structures of an arylpalladium complex with these organometallic reagents. Energy decomposition analysis (EDA)^25^ of these transition-state structures clearly indicates that small values of the deformation energy (DEF) are mainly responsible for the low activation barrier in the case of the copper reagent (Fig. 1D). In particular, the 9-triptycenylcopper unit involves a much lower DEF_TripM_ than those in the cases of boron and zinc, probably due to the favorable Cu(i)–Pd(ii) interaction, as well as the abundance of vacant sites around the copper center. Interestingly, the Zn(ii) center also shows some interaction with Pd(ii), but the DEFs of both the 9-triptycenyl zinc reagent and the arylpalladium complex are much larger. In the less-distorted **TS*_Cu_*1**, smooth delivery of the triptycenyl ligand is facilitated by the well-organized triangular arrangement of copper–palladium–carbon (Cu(i)–Pd(ii)–C). In addition, natural bond orbital (NBO)^26^ analysis showed that electron donation from Cu(i) to Pd(ii) is predominant in **IM*_Cu_*1** and **TS*_Cu_*1** (Fig. 1E), while the reverse donation from the Pd(ii) center to Lewis acidic Zn(ii) was observed in **TS*_Zn_*1**.^14^,23d,e These results reflect the characteristic reactivity of organocopper reagents arising from the high-energy d orbitals and vacant coordination sites of copper. Thus, transfer of the bulky triptycenyl ligand is promoted not only by the adjacency of the two metal centers, but also by the favorable electronic interaction between them.

## Conclusions

In conclusion, the experimental results and DFT calculations confirm that this organocopper cross-coupling reaction is a potent C–C bond-forming methodology with unprecedented applicability to 3D bulky molecules. The unique ability of copper to facilitate efficient transmetalation *via* a compact transition state arising from an efficient metal–metal interaction is the key to the success of this reaction. Thus, this reaction is quite efficient with high functional group tolerance, and applicable to both sp^2^- and sp^3^-substrates. Further investigations on the substrate scope and detailed reaction mechanism are the subjects of ongoing research.

## Conflicts of interest

There are no conflicts to declare.

## Supplementary Material

Supplementary information

Crystal structure data
