# Supplementary material for: Organocopper cross-coupling reaction for C–C bond formation on highly sterically hindered structures
Source: Chem Sci. 2019 May 10;10(24):6107–12. doi: 10.1039/c9sc00891h (PMC6585593; doi:10.1039/c9sc00891h)
Supplement: Supplementary file 1 [file SC-010-C9SC00891H-s001.pdf]

Electronic Supplementary Material (ESI) for Chemical Science.
This journal is © The Royal Society of Chemistry 2019

Supporting Information for

Organocopper Cross-coupling Reaction for C–C Bond Formation on Highly Sterically Hindered Structures

Miku Oi,[a,b] Ryo Takita,*[a,b] Junichiro Kanazawa,[a] Atsuya Muranaka,[b] Chao Wang[a] and Masanobu Uchiyama*[a,b]

correspondence to: takita@mol.f.u-tokyo.ac.jp, uchiyama@mol.f.u-tokyo.ac.jp

Table of Contents

1. General S1

2. Supplementary discussions S2

2-1. Optimization of reaction conditions S2

2-2. Mechanistic insights S8

3. Experimental section S24

3-1. General procedures S24

3-2. Characterization of the products S30

3-3. X-Ray crystallographic data S49

4. Computational details S52

5. Copies for NMR spectra S70

6. References S96

1. General

Instrumentation

NMR spectra were obtained on JEOL JNM-AL300, 400 or ECA-500, or Bruker AVANCE III HD 500, or Varian VNMR 500 spectrometers. Chemical shifts are expressed in δ (ppm) values referenced to tetramethylsilane as an internal standard, and coupling constants are expressed in hertz (Hz). The following abbreviations are used: s = singlet, d = doublet, t = triplet, q = quartet, sep = septet, m = multiplet, and br-s = broad singlet. IR spectra were obtained on a JSACO FT/IR-4700. Melting points were determined with a Yanaco micro melting point apparatus. ESI and APCI mass spectra were measured on a Bruker micrOTOF-II or micrOTOF-QIII spectrometer.

Materials

n-BuLi in n-hexane was obtained from Kanto Chemical Co. Ltd. The concentration of n-BuLi was determined by titration prior to use. $Pd(OAc)_2$ (>99.9%) was purchased from Aldrich, and CuI and CuCl were purchased from Wako Chemical Co. Ltd., and these reagents were used without any purification. Anhydrous THF was purchased from Wako Chemical Co. Ltd. Aryl iodides and bromides were used after appropriate purification (recrystallization or alumina column chromatography). All other chemicals were of reagent grade and used as received. Air- and moisture-sensitive manipulations were performed with standard Schlenk techniques under argon atmosphere. Normal-phase column chromatography was performed with silica gel 60 (230-400 mesh) from Merck or CHROMATREX FL60D from Fuji Silysia Chemical LTD. Reverse-phase column chromatography was performed with Flash ODS column from Yamazen Corporation.

2. Supplementary discussions

2-1. Optimization of reaction conditions

• Optimization with 9-triptycenyl substrates (Table 1 in the Main Text)

The corresponding copper reagent **1c** was prepared by the lithiation of 9-bromotriptycene, followed by the addition of copper salts. The appropriate solvent system was essential for the efficient lithiation[S1] (**Fig. S1**), the use of mixed solvent (THF:toluene = 2:3) solely afforded the 9-lithiated triptycene, and thus we decided to use this solvent system for the preparation of copper reagent as well as the following cross-coupling reaction.

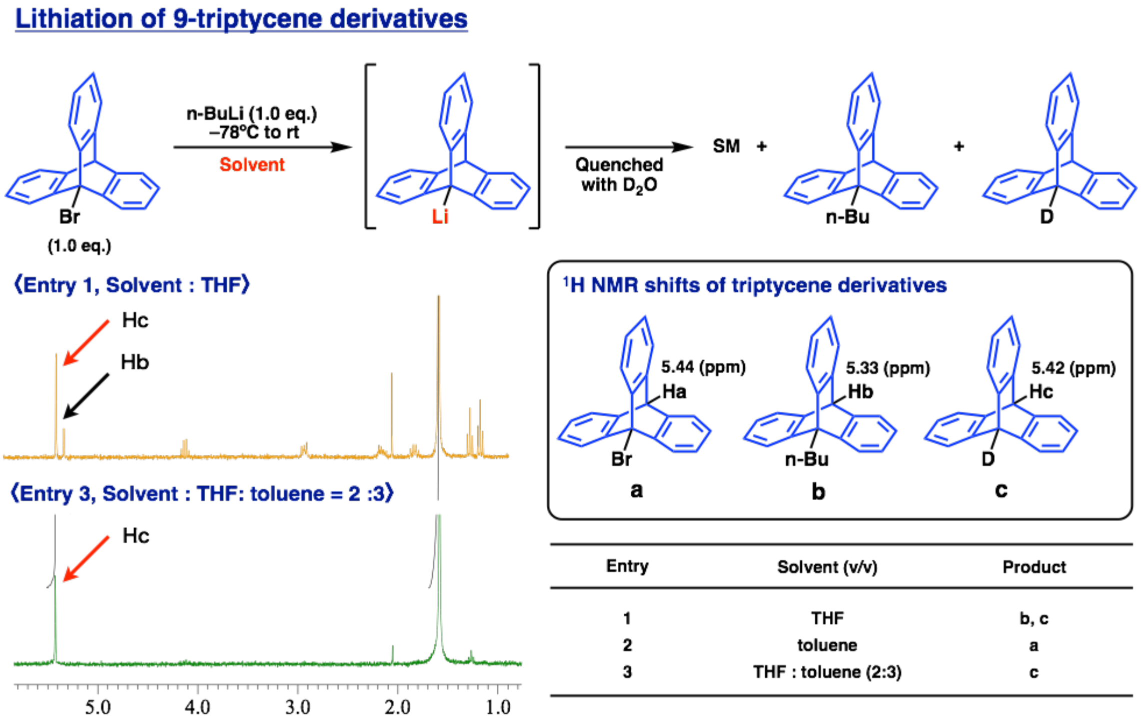

Entry	Solvent (v/v)	Product
1	THF	b, c
2	toluene	a
3	THF : toluene (2:3)	c

Fig. S1. Optimization of lithiation of 9-bromotriptycene.

In the cross-coupling reaction using 9-triptycenyl copper reagent (**1c**), ligand effects were examined with 1:1.2 Pd/ligand ratio (**Table S1**). Steric and electronic effects of phosphine ligands did not affect the chemical yield of product so much. Among screened under these conditions, **L1** and PPh_3 gave optimal chemical yields. It should be noted that the use of PMe_3 (**L7**) also promoted the reaction, which was utilized as a model for theoretical calculation (*vide infra*).

Table S1. Optimization of reaction conditions with 9-triptycenylcopper: *Ligand effects 1*.

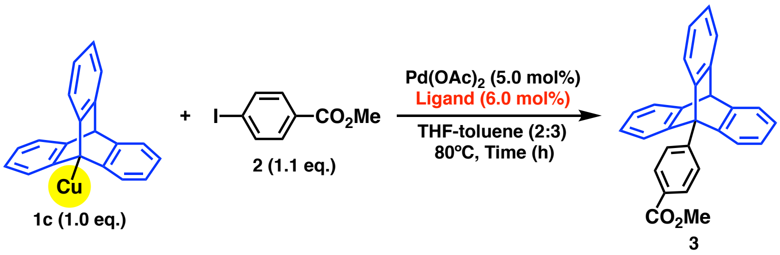

Entry	Ligand	Time (h)	Yield (%)[a]	Entry	Ligand	Time (h)	Yield (%)[a]
1	L1	25	84	8	PPh_3	4	82
2	L1	5	80 (80)	9	L7	4	64
3	L2	5	69	10	XantPhos	4	77
4	L3	4	77	11	L8	4	30
5	L4	4	33	12	L9	4	64
6	L5	4	74	13	L10	4	57
7	L6	4	74				

[a] ^{1}H NMR yields. (Isolated yield).

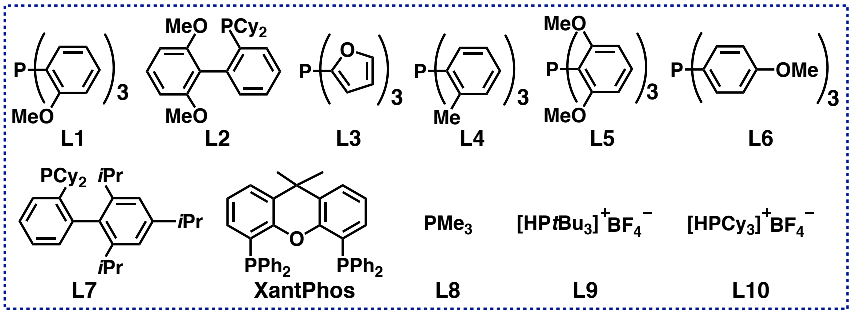

Pd/ligand ratio was also examined using **L1** as summarized in **Table S2**. While the change in Pd/ligand ratio did not cause big difference in these ranges (1.0:1.2 to 1.0:3.0), a slightly better result was obtained when **L1** was employed in a 1:3 ratio with $Pd(OAc)_2$ with a good reproducibility. In the case of PPh_3, such improvement was not observed even with a higher Pd/ligand ratio. Thus, we have determined the conditions in entry 4 as optimized conditions.

Table S2. Optimization of reaction conditions with 9-triptycenylcopper: *Ligand effects 2*.

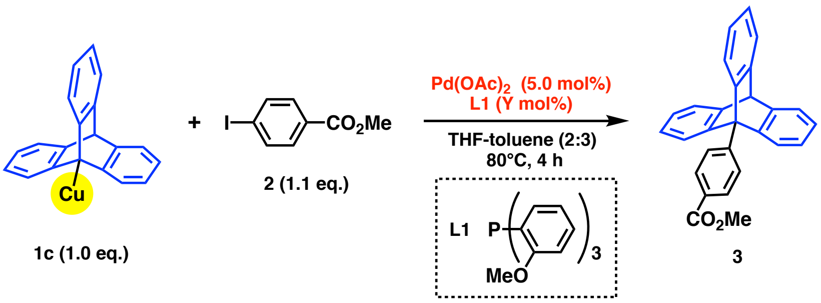

Entry	L1 (Y mol%)	$Pd(OAc)_2$: L1	Yield (%)[a]
1	6.0	1.0 : 1.2	83
2	10.0	1.0 : 2.0	76
3	12.5	1.0 : 2.5	80
4	15.0	1.0 : 3.0	86

[a] ^{1}H NMR yields.

• Optimization of cross-coupling reaction to afford 20 (Fig. 2 in the Main Text)

The cross-coupling reaction between 9-triptycenyl copper reagent and methyl bromoacetate, in which C(sp^3)–C(sp^3) bond is formed, was also possible under the similar conditions. Further improvement was observed when the palladium catalyst was replaced to a nickel catalyst (**Table S3**).

Table S3. Optimization of reaction conditions for C(sp^3)–C(sp^3) bond formations.

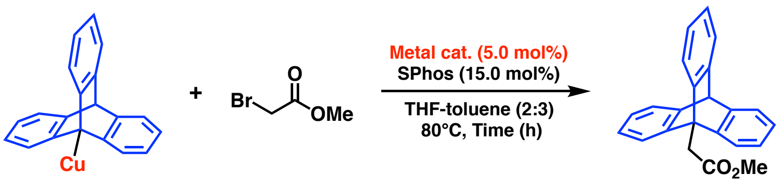

1c (1.0 eq.) (1.1 eq.) 20

Entry	Metal cat.	Time (h)	Yield (%)[a]
1	$Pd(OAc)_2$	24	41
2	$Ni(acac)_2$	24	54
3	$Ni(acac)_2$	53	76[b]

[a] ^{1}H NMR yields. [b] Isolated yield.

• **Optimization with mesityl-type substrates (Fig. 2 in the Main Text)**

With mesityl-type substrates, the corresponding copper reagents were prepared by the lithiation of aryl halides, followed by the addition of copper salts. It was found that the copper source and reaction temperatures during the preparation of copper reagents and the following cross-coupling reaction were crucial for the reaction outcome (**Table S4**).

Table S4. Optimization of reaction conditions with mesityl-type substrate: Copper source and reaction temperature.

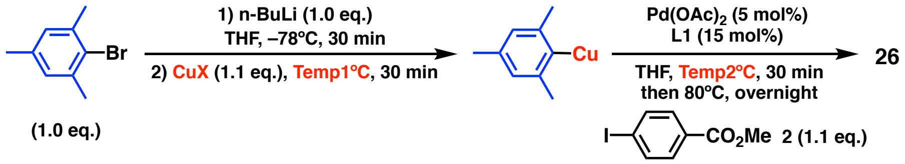

Entry	CuX	Temp1 (°C)	Temp2 (°C)	Yield (%)[a]
1	CuI	room temp	room temp	0
2	CuI	0	room temp	0
3	CuI	0	0	0
4	CuI	–78	room temp	Complex mixture
5	CuI	–78	0	88
6	CuCl	–78	0	Complex mixture

[a] ^{1}H NMR yields.

• **Comments on the preparation of copper reagents**

Much of copper reagents used in this manuscript were prepared simply by the addition of copper(I) salt (CuI or CuCl) to the lithiated substrates (triptycene or mesityl-type) or *t*-BuLi. On the other hand, adamantyl reagents were prepared from the corresponding 1- or 2-adamantyl zinc bromide (0.5 M THF solution commercially available from Aldrich). So, while the lithiation generally causes the narrow substrate scope, the broader functional group tolerance should be expected with available zinc reagents.

• **Preliminary results using catalytic amount of copper**

The use of catalytic amount of copper with the lithiated 9-triptycene (**1e**) gave low yield of the coupling product (i.e. **8**) (**Fig. S2**). GC-MS analysis revealed the generation of 9-iodotriptycene and 4,4'-dimethoxy-1,1'-biphenyl, suggesting that halogen-lithium exchange is a major side reaction.

On the other hand, the use of catalytic amount (10 mol%) of copper with 1-adamantylzinc bromide gave **21** in 35% yield. The trace amount of **21** was obtained without copper iodide. Further optimization of catalytic conditions is a subject of ongoing study.

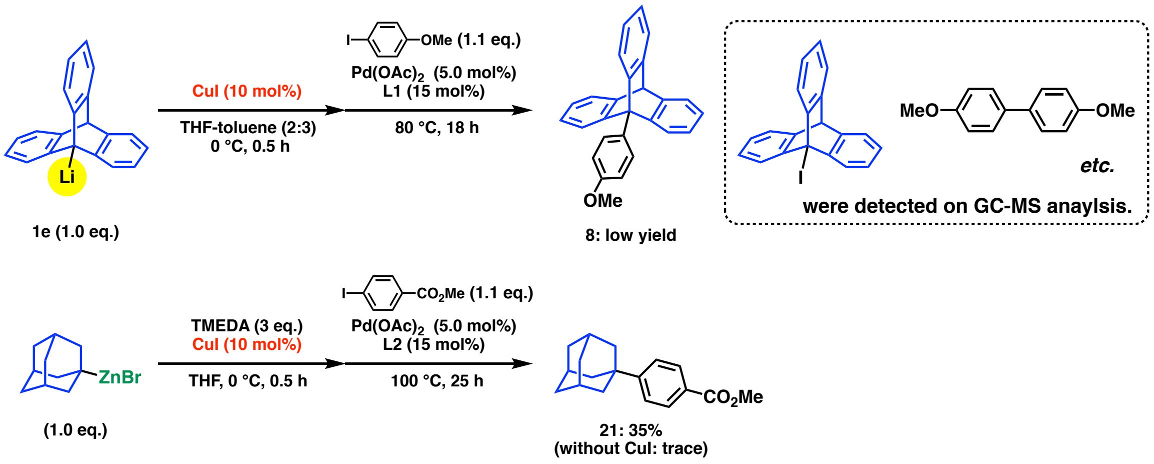

Fig. S2. Preliminary investigations using catalytic amoung of copper.

2-2. Mechanistic insights

• Experimental results

To gain insight into the mechanism of the present reaction, we examined the effects of radical-trapping reagent. The addition of 9,10-dihydroanthracene or 2,2,6,6-tetramethylpiperidine-1-oxyl (TEMPO) as a radical-trapping reagent to optimized conditions had little effect on the product yields, ruling out significant involvement of radical pathways in the present copper-mediated reaction (**Fig. S3**).

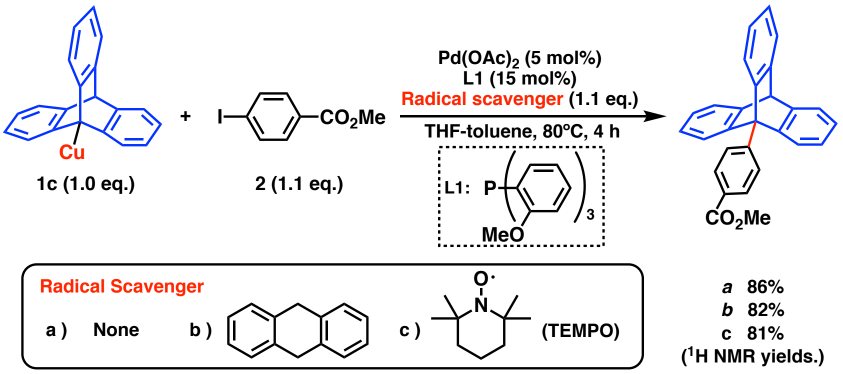

Fig. S3. Control experiments with radical scavengers.

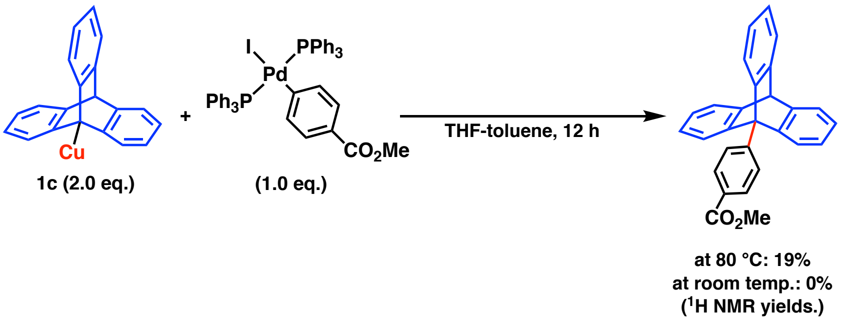

Fig. S4. Control reaction of **1c** with the isolated aryl-Pd(II) complex.

In addition, the control reaction in entry 5, Table 1 (in the manuscript) indicated that a Pd catalyst is necessary for the cross-coupling reaction. Further, **1c** reacted with aryl-Pd(II) complex ($Pd(Ar)I(PPh_3)_2$) at 80 °C to achieve the desired C–C bond formation (**Fig. S4**). With these mechanistic implications in hand, the following catalytic cycle was considered; the cycle was based on three fundamental steps, that is, oxidative addition, transmetalation, and reductive elimination, as broadly observed in palladium-catalyzed cross-coupling

reaction (**Fig. S5**). The rate determining step should be either transmetalation or reductive elimination steps, since the reaction of **1c** with aryl-Pd(II) complex in **Fig. S4** did not proceed at all at room temperature.

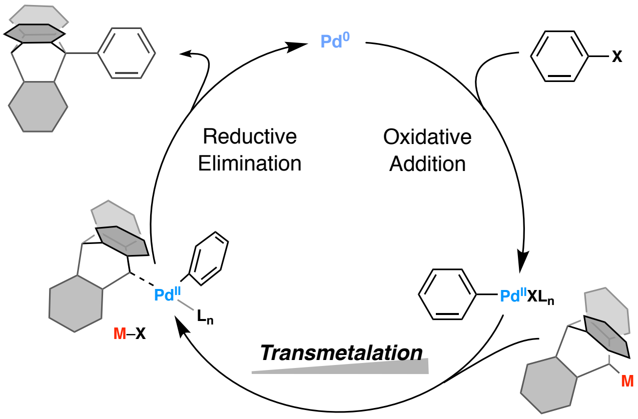

Fig. S5. Catalytic cycle of palladium-catalyzed cross-coupling reaction.

The copper reagents utilized in this study were prepared from the corresponding lithium reagents and copper(I) halides. Although the detailed structure of 9-triptycenyl copper reagent is not clear at this moment, the following experiments revealed that the resulting lithium halides in the reaction mixture are not crucial for the reaction. As shown in **Fig. S6**, the lithium salt-free mesityl copper reagent prepared according to the literature procedure[S2] was able to provide the desired product in a slightly lower yield.

Although much of alkyl/aryl copper reagents should be in an oligomeric form in solution, or have the coordinating compounds such as solvent molecules, the monomeric 9-triptycenyl copper was used as a model substrate to make the discussion simplified.

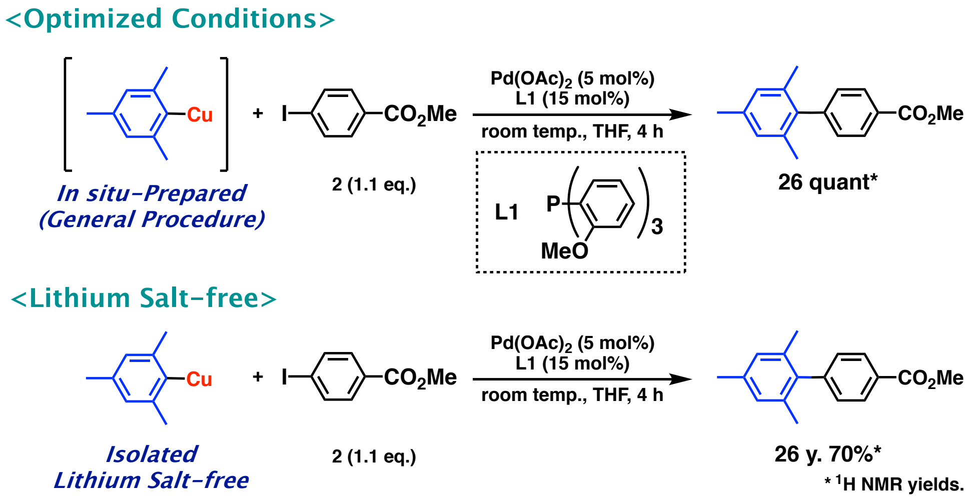

Fig. S6. Cross-coupling reactions using in situ-prepared or lithium salt-free mesityl copper reagents.

• Results of theoretical investigations

The properties of organometallic reagents should have the momentous effects on the transmetalation step (**Fig. 1** and **Fig. S5**). Based on the experimental results, we performed theoretical calculations to identify the detailed reaction process during the transmetalation with copper reagents as well as zinc and boron reagents.

¶ DFT calculation methods

Among the DFT methods employed for the related metal-catalyzed reactions[S3], a *classical* method, B3LYP[S4], and M06-2X[S5] and *ω*B97X-D[S6] were compared. SDD basis set for Pd, Cu, Zn, and Br atoms and 6-31+G* basis set for all other atoms were used. The obtained results were summarized in **Fig. S7**.

By the comparison of these results, it was found that: 1) basically, similar reaction pathways and results were obtained using any of three methods, and the same chemistry can be discussed in any cases. In particular, the compact transition state *via* Cu–Pd interaction was obtained when the copper reagent was employed, and 2) weak interactions were well reproduced with M06-2X and *ω*B97X-D. Given that the accuracy for the details as well as the calculation costs, *ω*B97X-D was used for the discussions in this study.

● *Transmetalation step with triptycene-Cu complex*

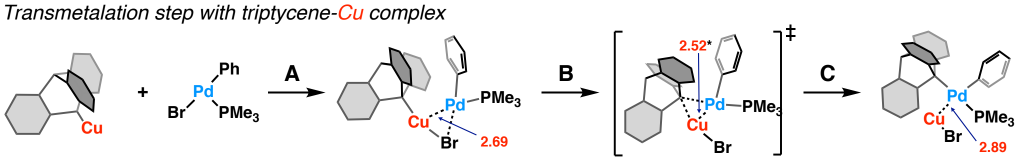

Methods	A	B	C
B3LYP	$\Delta E = -28.6$<br>$\Delta G = -16.1$	$\Delta E = +22.1$<br>$\Delta G = +22.4$	$\Delta E = -13.0$<br>$\Delta G = -13.0$
M06-2X	$\Delta E = -48.8$<br>$\Delta G = -33.1$	$\Delta E = +20.0$<br>$\Delta G = +20.0$	$\Delta E = -10.0$<br>$\Delta G = -11.4$
ωB97X-D	$\Delta E = -46.6$<br>$\Delta G = -31.4$	$\Delta E = +20.9$<br>$\Delta G = +21.4$	$\Delta E = -14.6$<br>$\Delta G = -15.8$

● *Transmetalation step with triptycene-Zn complex*

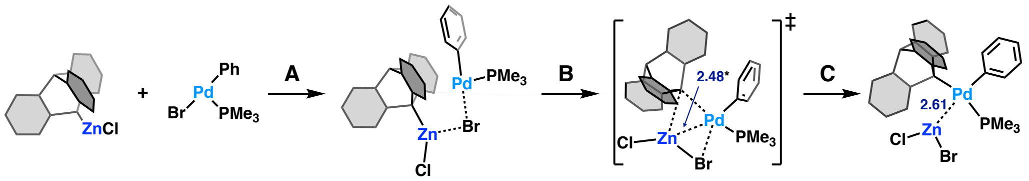

Methods	A	B	C
B3LYP	$\Delta E = -12.7$<br>$\Delta G = +2.3$	$\Delta E = +24.1$<br>$\Delta G = +24.9$	$\Delta E = -4.2$<br>$\Delta G = -6.1$
M06-2X	$\Delta E = -40.4$<br>$\Delta G = -23.9$	$\Delta E = +29.7$<br>$\Delta G = +29.7$	$\Delta E = -12.0$<br>$\Delta G = -13.4$
ωB97X-D	$\Delta E = -38.0$<br>$\Delta G = -21.3$	$\Delta E = +29.0$<br>$\Delta G = +28.0$	$\Delta E = -2.6$<br>$\Delta G = -2.5$

● *Transmetalation step with triptycene-B complex*

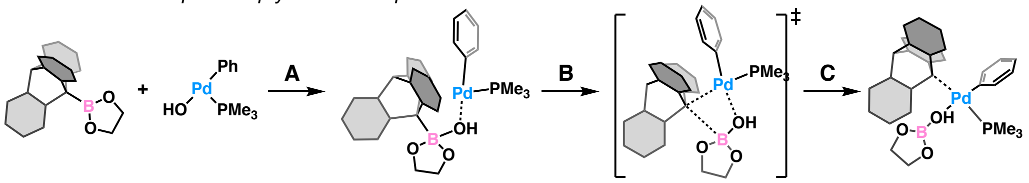

Methods	A	B	C
B3LYP	$\Delta E = -9.8$<br>$\Delta G = +8.5$	$\Delta E = +44.1$<br>$\Delta G = +41.7$	$\Delta E = -48.7$<br>$\Delta G = -49.8$
M06-2X	$\Delta E = -45.8$<br>$\Delta G = -26.4$	$\Delta E = +43.8$<br>$\Delta G = +42.0$	$\Delta E = -37.8$<br>$\Delta G = -39.3$
ωB97X-D	$\Delta E = -40.8$<br>$\Delta G = -21.8$	$\Delta E = +48.2$<br>$\Delta G = +46.4$	$\Delta E = -44.7$<br>$\Delta G = -46.1$

* The selected bond lengths at the ωB97X-D/SDD (for Pd, Cu, Zn and Br) & 6-31+G* (for other atoms) level of theory are shown in Å.

Fig. S7. Modeled pathways for the transmetalation step between arylpalladium complex and 9-metalated triptycene complexes with various calculation methods.

¶ Comparison of transmetalation with copper, zinc, and boron reagents

The computed pathways for transmetalation using copper, zinc, and boron reagents were summarized in **Fig. S8**. The use of copper reagent gave the most favorable pathway with a low activation barrier. In the case of zinc, while the activation barrier was not too high, the reaction pathway was clearly unstabilized, where the energy of **IM$_{Zn}$2** was higher than the corresponding reactant **RT**. The activation energy with boron reagent was too high to enable the transmetalation under the reaction conditions. Thus, the further details will be discussed mainly by focusing on the copper and zinc cases in the following discussions.

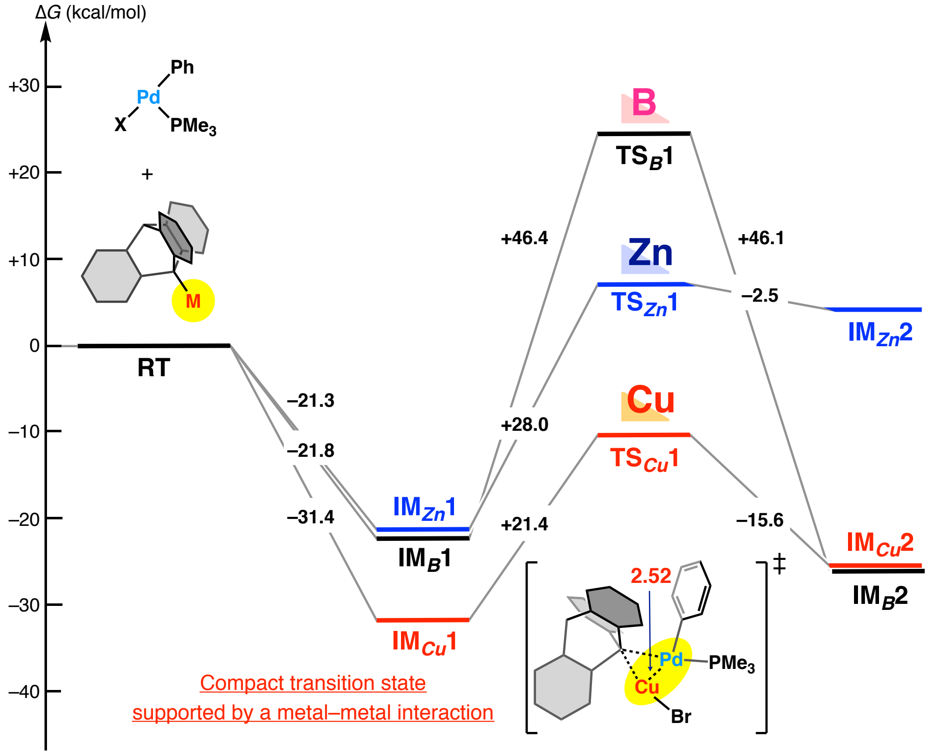

Fig. S8. Energy diagrams for the transmetalation step between arylpalladium and 9-metalated triptycene complexes.

Energy changes and bond lengths at the *ω*B97X-D/SDD & 6-31+G* level of theory are shown in kcal/mol and Å, respectively.

¶ The consequent reductive elimination: Reaction profiles with copper and zinc

Fig. S9 summarizes the reaction profile using the copper reagent for the transmetalation and following reductive elimination steps (cf. **Fig. 1C**). **TS*Cu*1** represents for the transition state of transmetalation and **TS*Cu*2** represents for the transition state of reductive elimination. The formation of **IM*Cu*1** (–31.4 kcal/mol from **RT** by the coordination of arylpalladium complex with 9-triptycenyl copper reagent) and **IM*Cu*2** (–15.6 kcal/mol from **TS*Cu*1**) gave the large stabilization. As a result, the relative Gibbs energies of **TS*Cu*1** and **TS*Cu*2** are more stable than that of **RT**. In total, all of intermediates and transition states are in low energies and the activation barriers for both **TS*Cu*1** and **TS*Cu*2** are reasonably low (+21.4 kcal/mol and 19.8 kcal/mol, respectively).

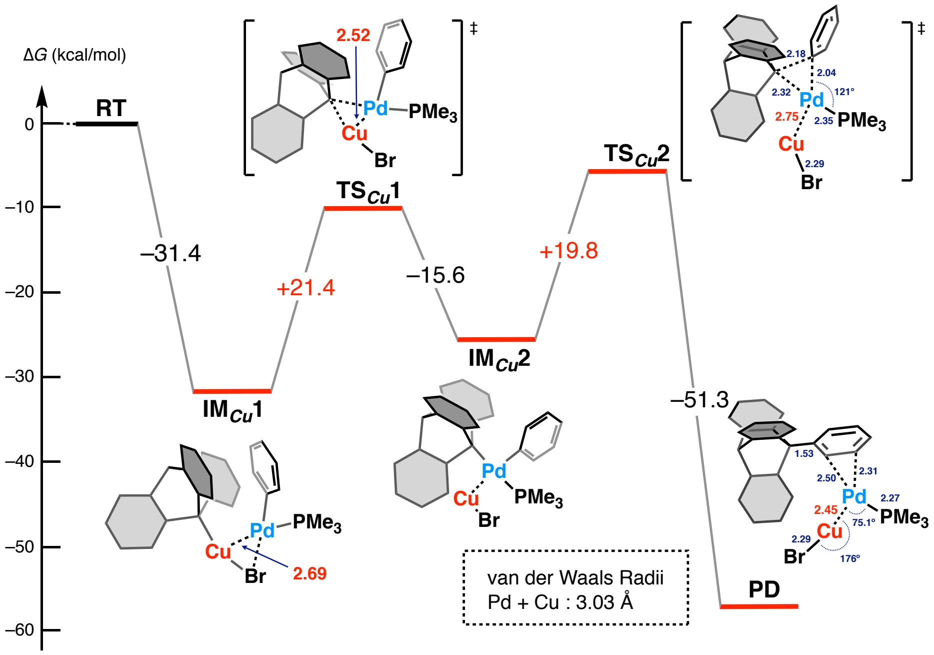

Fig. S9. Energy diagrams for the transmetalation and reductive elimination steps between arylpalladium and 9-triptycenyl copper complexes.

Energy changes and bond lengths at the *ω*B97X-D/SDD & 6-31+G* level of theory are shown in kcal/mol and Å, respectively.

Fig. S10 summarizes the reaction profile without copper reagent (CuBr) for the reductive elimination step. **TS2"** represents the transition state of reductive elimination. The activation energy of reductive elimination step without CuBr (+20.1 kcal/mol) is almost same as that with copper salt (CuBr) (+19.8 kcal/mol, in **Fig. 1C** or **Fig. S9**). So, the presence of copper should have a minimal effect on the reductive elimination step.

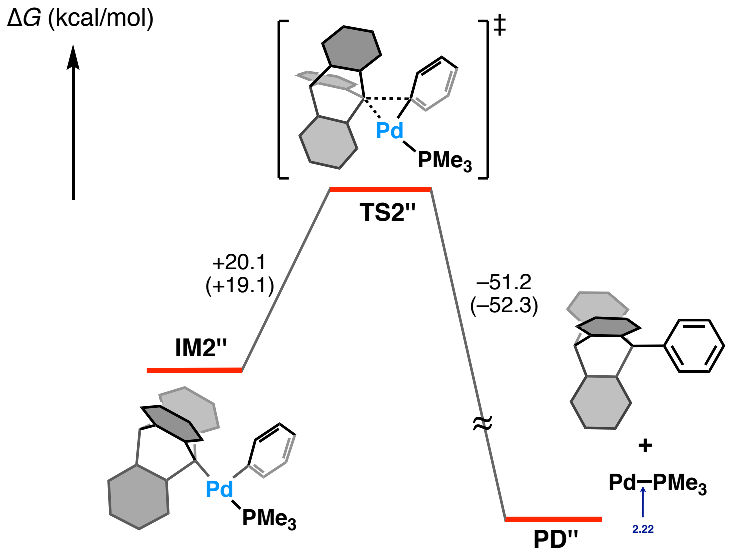

Fig. S10. Energy diagrams for the reductive elimination steps without copper salt (CuBr). Energy changes and bond lengths at the *ω*B97X-D/SDD & 6-31+G* level of theory are shown in kcal/mol and Å, respectively.

Fig. S11 summarizes the reaction profile using the zinc reagent for the transmetalation and following reductive elimination steps. **TS*Zn*1** represents for the transition state of transmetalation and **TS*Zn*2** represents for the transition state of reductive elimination. While the formation of **IM*Zn*1** (–21.3 kcal/mol from **RT**) provided the stabilization, **IM*Zn*2** was unstable compared with **RT**, due to the high activation barrier (+28.0 kcal/mol to **TS*Zn*1**) and poor stabilization (–2.5 kcal/mol) from **TS*Zn*1**. Thus, the energy of transition state for reductive elimination (**TS*Zn*2**) is rather high (+16.6 kcal/mol from **RT**) and the reaction pathway should be unfavorable compared with the copper case (**Fig. S9**).

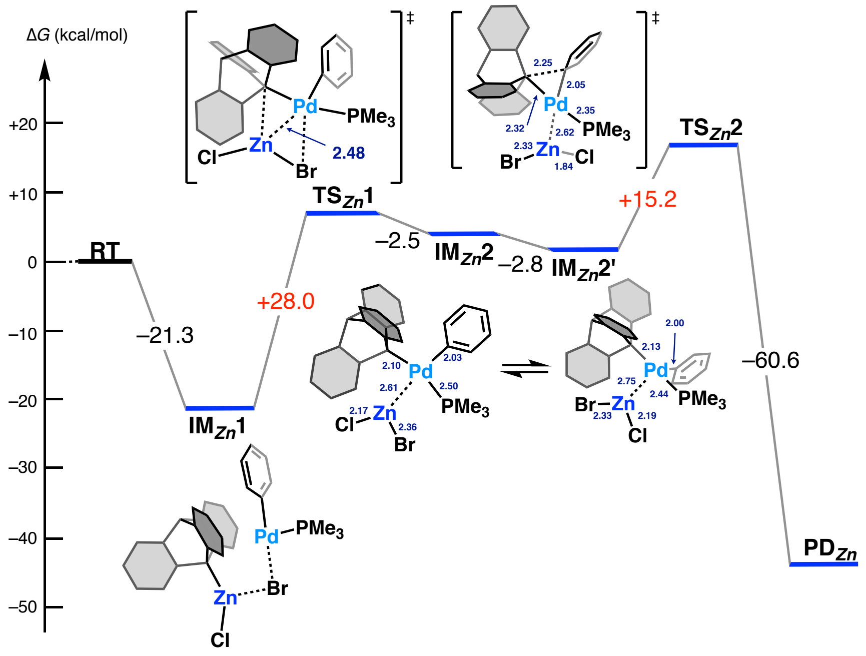

Fig. S11. Energy diagrams for the transmetalation and reductive elimination steps between arylpalladium and 9-triptycenylzinc chloride complexes.

Energy changes and bond lengths at the *ω*B97X-D/SDD & 6-31+G* level of theory are shown in kcal/mol and Å, respectively.

¶ The origin of efficient transmetalation with 9-triptycenyl copper reagent

<Energy Decomposition Analysis (EDA) of IM1>

- *with triptycene-Cu complex*
- *with triptycene-Zn complex*

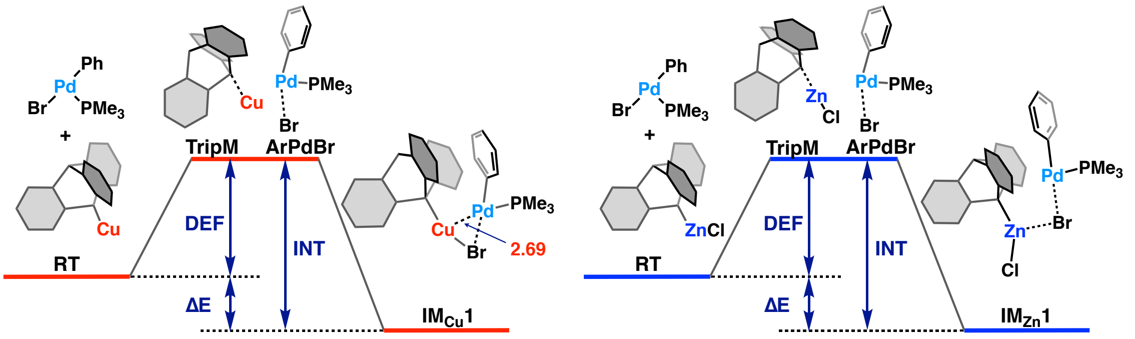

M	DEF_{TripM}	DEF_{ArPdBr}	DEF_{total}	INT	ΔE	ΔG
Cu	0.7	104.4	105.1	–151.8	–46.1	–31.4
Zn	9.2	3.5	12.7	–50.7	–38.1	–21.3

(kcal/mol)

Fig. S12. Energy decomposition analysis for the **IM*Cu*1** and **IM*Zn*1** at the *ω*B97X-D/SDD & 6-31+G* level of theory.

Firstly, the large stabilization was observed in the formation of **IM*Cu*1** from **RT**, compared with that of **IM*Zn*1**. Energy decomposition analysis (EDA)[S7] were performed with these complexes at the *ω*B97X-D/SDD & 6-31+G* level of theory (**Fig. S12**). In the zinc case (**IM*Zn*1**), the total deformation energy (**DEF_{total}**) was mostly associated with 9-trypticenyl zinc unit, probably because the bending of C_{Trip}–Zn–Cl should be necessary to the coordination with bromide atom. On the other hand, **DEF_{TripCu}** is quite small (0.7 kcal/mol) due to the linear structure of the copper reagent. Very large interaction energy (–151.8 kcal/mol) fully compensated the unfavorable energy loss by **DEF_{ArPdBr}**, implying that this interaction includes not only the ionic interaction between Br and Cu, but also the Pd–Cu interaction. Natural bond orbital (NBO)[S8] analysis (at the *ω*B97X-D/LanL2DZ & 6-31G* level of theory) revealed that this interaction consists of the electron donation from copper center (mainly 3d_{xy} orbital) to the anti-bonding orbital between palladium and phosphine (**Fig. S13**).

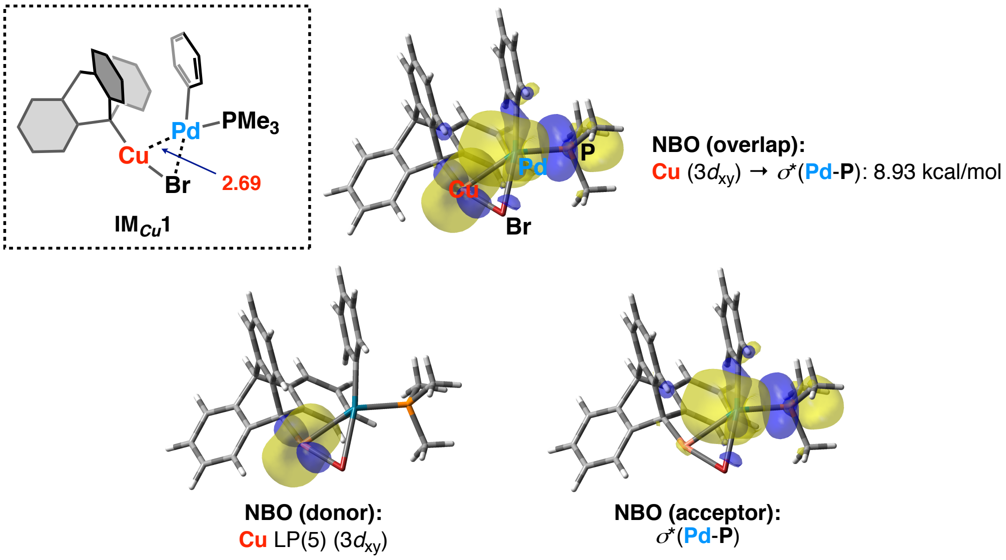

Fig. S13. Donor-acceptor overlap of natural bond orbitals within **IM*Cu*1** at the *ω*B97X-D/LanL2DZ & 6-31G* level of theory.

Next, the transition states for the transmetalation were investigated in detail. **Fig. S14** presents the structures of **TS*Cu*1** and **TS*Zn*1**. **TS*Cu*1** adopts the square planar structure around palladium center, while the bromine atom has to go downward from the palladium plane in the case of **TS*Zn*1**, resulting in a distorted structure. The results of EDA showed these phenomena quantitatively, the total deformation energy of **TS*Zn*1** was much larger than that of **TS*Cu*1** (**DEF$_{total}$** for **TS*Zn*1**: 102.6 kcal/mol, and **DEF$_{total}$** for **TS*Cu*1**: 47.4 kcal/mol, at the *ω*B97X-D/SDD & 6-31+G* level of theory) (**Fig. 1D** in the manuscript and **Fig. S15**). NBO analysis revealed that electronic properties were also different in these complexes. The donation from copper to palladium was weakened during the course of transmetalation (**Fig. 1E** in the manuscript and **Fig. S16** top), probably because the transfer of triptycene ligand should make the Pd center more electron-rich and the Cu center more electron-deficient. These perturbated electronic interactions between copper and palladium should be crucial for the compact transition state structure, enabling the efficient delivery of bulky triptycenyl ligand. In contrast, zinc center acts as Lewis acid in **TS*Zn*1**, and the electron-donation is necessary from the palladium *d*-electrons (**Fig. S16** bottom).

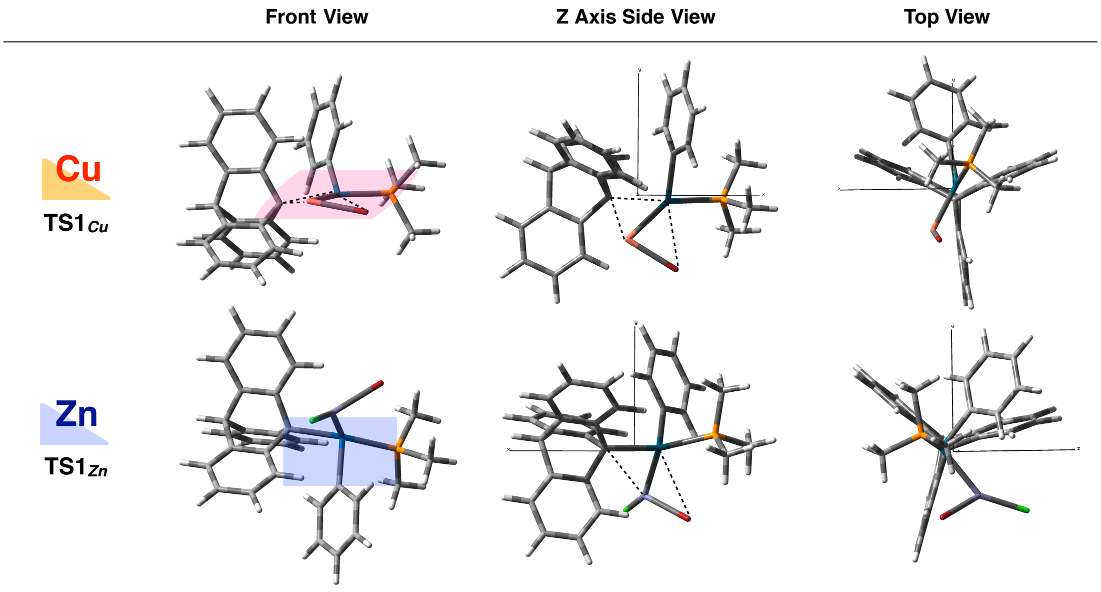

Fig. S14. Comparison of transition state structures (**TS$_{Cu}$1** and **TS$_{Zn}$1**).

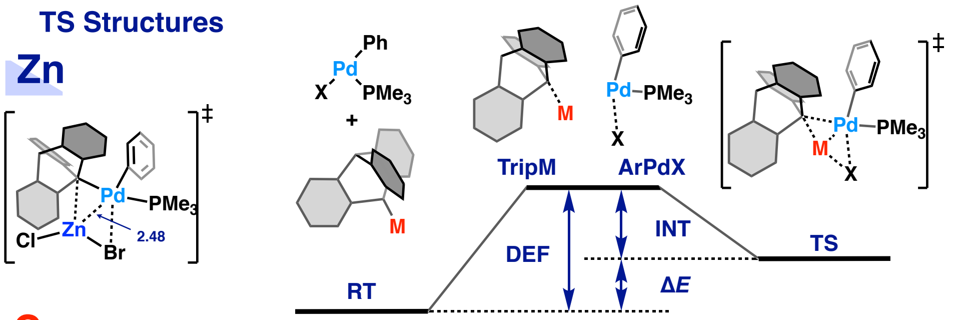

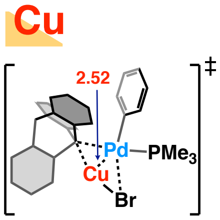

M	DEF$_{TripM}$	DEF$_{ArPdX}$	DEF$_{total}$	INT	*ΔE*	*ΔG*
Zn	75.3	27.3	102.6	–111.7	–9.1	6.7
Cu	32.5	14.9	47.4	–73.1	–25.7	–10.0

(kcal/mol)

Fig. S15. Energy decomposition analysis for the **TS$_{Cu}$1** and **TS$_{Zn}$1** at the *ω*B97X-D/SDD & 6-31+G* level of theory.

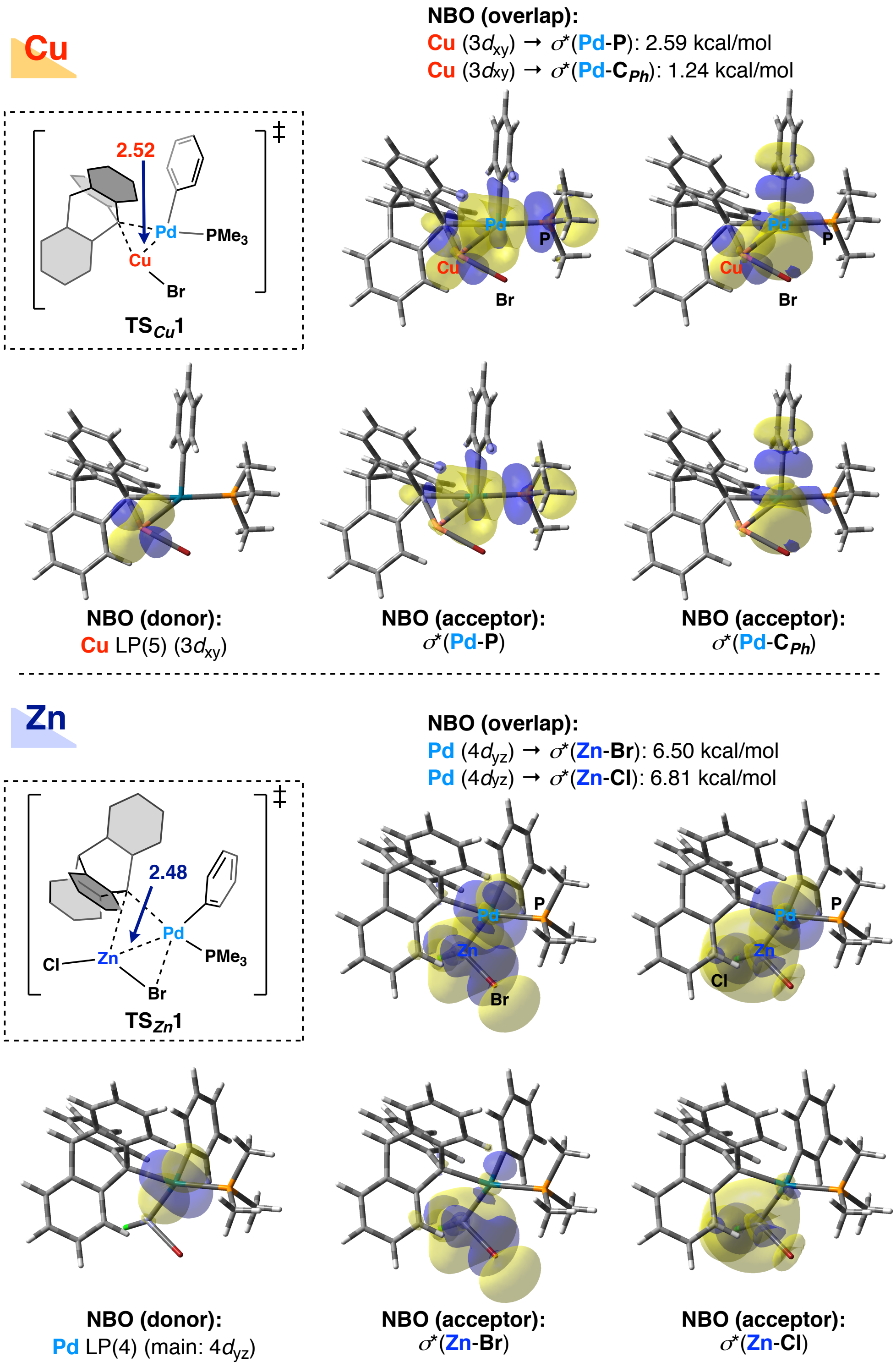

Fig. S16. Donor-acceptor overlap of natural bond orbitals within **TS*Cu*1** (top) and **TS*Zn*1** (bottom) at the *ω*B97X-D/LanL2DZ & 6-31G* level of theory.

¶ Additional discussions on structure of modeled arylpalladium complexes

In this study, *trans*-Pd(Ph)Br(PMe_3) complex was used as a model arylpalladium complex. The precise structure under the reaction conditions is not defined. But this model structure should be reasonable due to the following reasons.

1) The experimental results showed that the structure (and amount) of phosphine ligands did not affect the reaction outcome so much (**Table S1** and **S2**). In particular, good yields were obtained with a variety of ligands that gave the different structures of arylpalladium complexes through the oxidative addition of aryl halides[S9] (**Table S5**).
2) In entry 3 of **Table S5**, the use of PPh_3 afforded the optimal results. It is reported that the transmelation proceeds with the corresponding *trans*-complex when PPh_3 was used.[S10]
3) Our calculation revealed that the *trans*-configuration should be most stable among the *cis*- and *trans*-Pd(Ph)Br(PMe_3) complexes at the ωB97X-D/SDD & 6-31+G* level of theory. Furthermore, it was experimentally found that the use of PMe_3 as a ligand promoted the reaction (**Table S1**).

It should be noted that, while the structure of arylpalladium may vary under the reaction conditions, the main chemistry in transmetalation should be the key, that is, the compact transition state *via* Pd–Cu interaction that enables the delivery of bulky triptycenyl ligand.

Table S5. Ligand effects and the structures of arylpalladium intermediates.

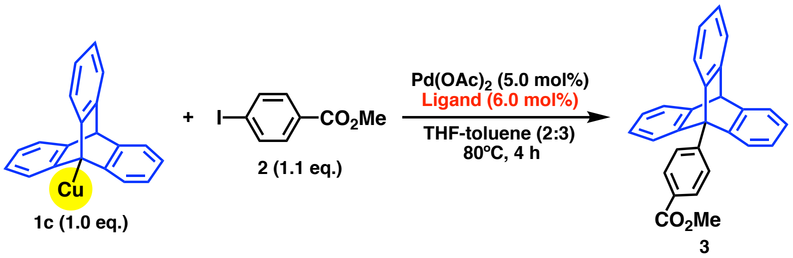

Entry		Ligand	Arylpalladium Complex Structure		Yield (%)[a]
1	L2	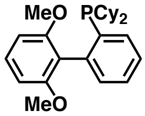	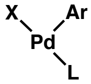	*cis*-complex	69
2		[HP*t*Bu$_3$] BF$_4$			64
3		PPh$_3$	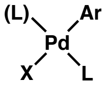	*trans*-complex	82
4		[HPCy$_3$] BF$_4$			57
5	XantPhos	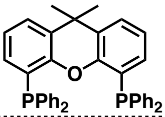	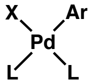	*cis*-complex	77
6	L3	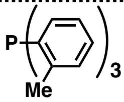	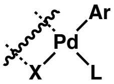	dimer (*trans*-complex)	33

[a] ^{1}H NMR yields.

¶ The results of calculation with 2,6-dimethylphenyl reagents as “non-triptycene” model substrate

To prove the versatility of the above-mentioned mechanism, the calculations were performed with 2,6-dimethylphenyl copper and boron reagents as “non-triptycene” model substrates (**Fig. S17**). Compared with the case of triptycene, much lower activation energies were observed in both cases. In the copper case, the mechanism based on Cu-Pd interaction still facilitated the efficient ligand transfer. In the boron case, the moderate activation energy is consistent with the precedents that enabled the bond formation of sterically-hindered biaryls by Suzuki-Miyaura cross-coupling reactions (See Ref. 7 in Manuscript).

• *Transmetalation with 2,6-dimethylphenylcopper reagent*

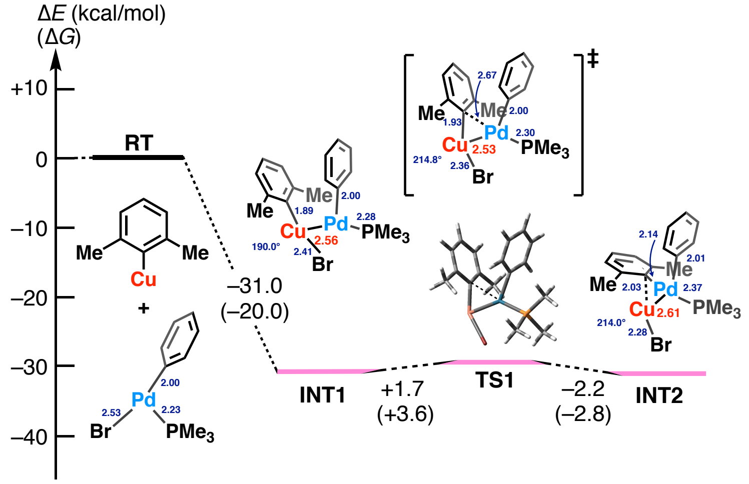

• *Transmetalation with 2,6-dimethylphenylboronic acid ester reagent*

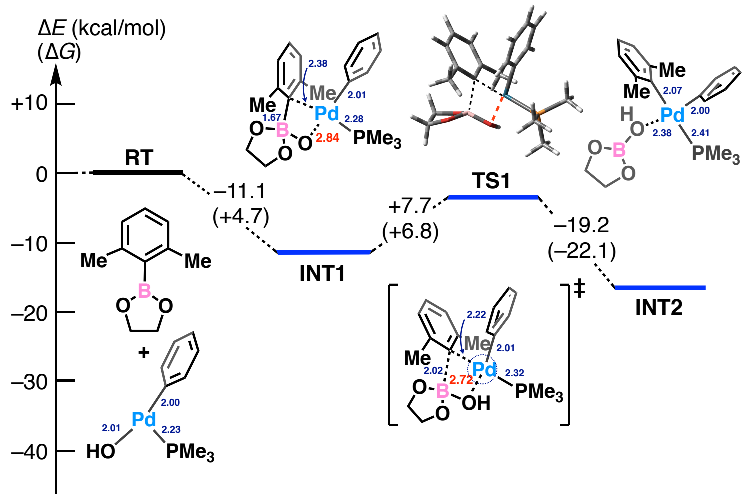

Fig. S17. Theoretical calculations for the transmetalation step between arylpalladium complex with 2,6-dimenthylphenylcopper reagent (top) and 2,6-dimemethylphenylboronic acid glycol ester (bottom) at the *ω*B97X-D/SDD&6-31+G* level of theory.

3. Experimental section

3-1. General procedures

Representative procedure 1: The cross-coupling reaction of 9-triptycenyl copper reagent

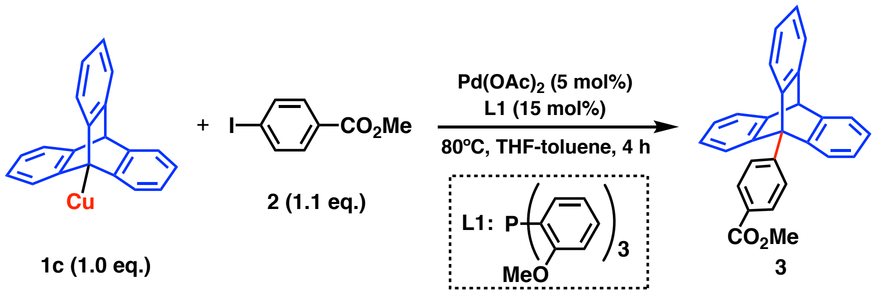

9-Bromotriptycene (166.1 mg, 0.50 mmol) was charged in a flame-dried Schlenk flask, and 5.0 mL of anhydrous THF and 7.5 mL of anhydrous toluene were added at room temperature under an argon atmosphere. The solution was cooled to –78 ºC and n-BuLi (2.60 M in n-hexane, 0.55 mmol) was added. The mixture was stirred at room temperature for 1.5 hours. The solution was cooled to 0ºC and copper(I) iodide (104.7 mg, 0.55 mmol) was added. The reaction mixture was warmed to room temperature and stirred for 30 min. Then, a solution of palladium(II) acetate (5.6 mg, 0.025 mmol), tris(*o*-methoxyphenyl)phosphine (**L1**) (27.6 mg, 0.075 mmol), and methyl 4-iodobenzoate (**2**) (144.1 mg, 0.55 mmol) in 3.0 mL of anhydrous THF was added *via* a cannula at the same temperature. Stirring was continued at 80 °C for 4 h, and formation of the desired compound was checked by TLC and GC-MS analyses. The reaction mixture was filtered through a short pad of Celite, and diluted with AcOEt. The mixture was washed with saturated NH_4Cl aq. (5 mL×3) and brine, dried over Na_2SO_4, and evaporated in vacuo. The chemical yield was determined by ^{1}H NMR analysis using dimethylsulfone (18.9 mg, 0.20 mmol) as an internal standard. The NMR yield of **3** was determined as 86%. The obtained material was purified by silica gel flash column chromatography (eluent: AcOEt/n-hexane) to give **3** in 83% isolated yield.

Representative procedure 2: The cross-coupling reaction of adamantyl copper reagent

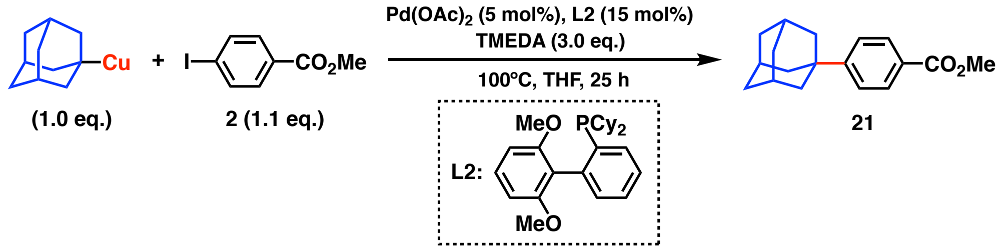

To the solution of TMEDA (174 mg, 1.5 mmol) in 4.0 mL of anhydrous THF in a flame-dried Schlenk tube, 1-adamantylzinc bromide (0.5 M in THF, 1.0 mL, 0.5 mmol) was added at 0 °C under an argon atmosphere. Then, copper(I) iodide (104.7 mg, 0.55 mmol) was added at 0 ºC. The reaction mixture was stirred for 30 min. Then, a solution of palladium(II) acetate (5.6 mg, 0.025 mmol), SPhos (**L2**) (30.7 mg, 0.075 mmol), and methyl 4-iodobenzoate (**2**) (144.1 mg, 0.55 mmol) in 3.0 mL of anhydrous THF was added *via* a cannula at 0 ºC. Stirring was continued at 100 ºC for 25 h, and formation of the desired compound was checked by TLC and GC-MS analyses. The reaction mixture was filtered through a short pad of Celite, and diluted with AcOEt. The mixture was washed with saturated NH_4Cl aq. (5 mL×3) and brine, dried over Na_2SO_4, and evaporated in vacuo. The obtained material was purified by normal-phase silica gel flash column chromatography (eluent: AcOEt/n-hexane) and then reverse-phase silica gel flash column chromatography (eluent: $MeOH/H_2O$) to give **21** in 43% isolated yield.

Representative procedure 3: The cross-coupling reaction of *tert*-butyl copper reagent

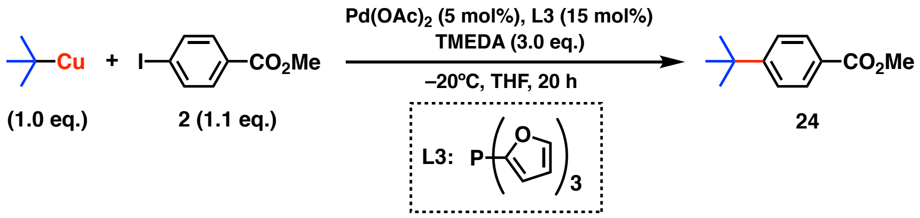

Copper(I) iodide (104.9 mg, 0.55 mmol) was charged in a Schlenk flask and dried under reduced pressure. Then, 5.0 mL of anhydrous THF and TMEDA (225 μL, 1.50 mmol) were added at room temperature under an argon atmosphere. The solution was cooled to –78 ºC and *t*-BuLi (1.56 M in n-hexane, 0.50 mmol) was added. The mixture was stirred at the same temperature for 30 min. Then, a solution of palladium(II) acetate (5.6 mg, 0.025 mmol), tris(2-furyl)phosphine (**L3**) (17.4 mg, 0.075 mmol), and methyl 4-iodobenzoate (**2**) (144.2 mg, 0.55 mmol) in 3.0 mL of anhydrous THF was added *via* a cannula at –78 ºC. Stirring was continued at –20 ºC for 20 h, and formation of the desired compound was checked by GC-MS analysis. The reaction mixture was quenched with MeOH, and then was filtered through a short pad of Celite, and diluted with AcOEt. The mixture was washed with saturated NH_4Cl aq. (5 mL×3) and brine, dried over Na_2SO_4, and evaporated in vacuo. The obtained material was purified by normal-phase silica gel flash column chromatography (eluent: AcOEt/n-hexane) and then reverse-phase silica gel flash column chromatography (eluent: MeOH/H_2O) to give **24** in 55% isolated yield. This compound is unexpectedly volatile.

Representative procedure 4: The cross-coupling reaction of mesityl-type substrates

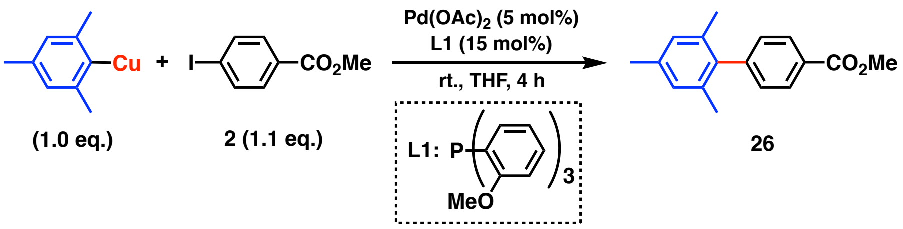

1-Bromo-2,4,6-trimethylbenzene (99.5 mg, 0.5 mmol) was charged in a flame-dried Schlenk flask using a syringe, and 5.0 mL of anhydrous THF was added under an argon atmosphere. The solution was cooled to –78 ºC and *t*-BuLi (1.61 M in n-hexane, 1.00 mmol) was added. The mixture was stirred at the same temperature for 30 min. Copper(I) iodide (104.7 mg, 0.55 mmol) was added at –78 ºC, and the reaction mixture was warmed to 0 ºC and stirred for 30 min. Then, solution of palladium(II) acetate (5.6 mg, 0.025 mmol), tris(*o*-methoxyphenyl)phosphine (**L1**) (26.4 mg, 0.075 mmol), and methyl 4-iodobenzoate (**2**) (144.1 mg, 0.55 mmol) in 3.0 mL of anhydrous THF was added *via* a cannula at 0 ºC. Stirring was continued at room temperature for 4 h, and formation of the desired compound was checked by TLC and GC-MS analyzes. The reaction mixture was filtered through a short pad of Celite, and diluted with AcOEt. The mixture was washed with saturated NH_4Cl aq. (5 mL×3) and brine, dried over Na_2SO_4, and evaporated in vacuo. The obtained material was purified by silica gel flash column chromatography (eluent: AcOEt/n-hexane) to give **26** in 97% isolated yield.

Synthesis of 4-Iodo-*N*,*N*-diisopropylcubane-1-carboxamide (S1)

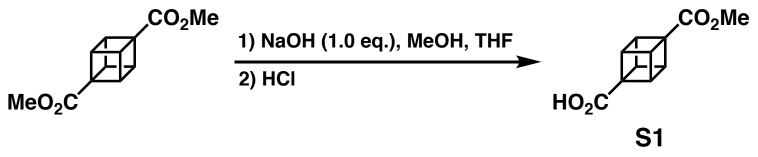

4-Methoxycarbonylcubane carboxylic acid (S1)

This compound was prepared according to the literature procedure.[S11] The use of dimethyl 1,4-cubanedicarboxylate (1.10 g, 5.00 mmol) as a starting material yielded 828 mg (80%) of 4-methoxycarbonylcubane carboxylic acid (**S1**), **^{1}H NMR (500 MHz, $CDCl_3$)**: δ 4.28-4.26 (m, 6H), 3.71 (s, 3H). These NMR data are in accordance with the literature data.

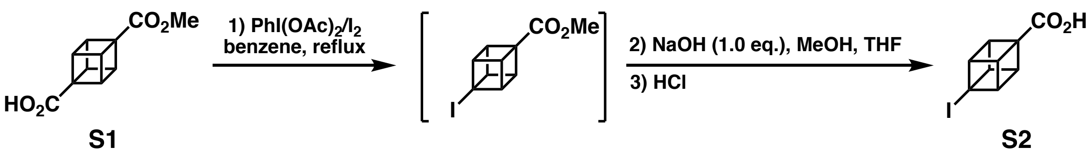

Synthesis of 4-Iodocubanecarboxylic acid (S2)

This compound was prepared according to the literature procedure.[S12] The use of **S1** (1.10 g, 5.34 mmol) as a starting material yielded 1.40 g (96%) of 4-iodocubanecarboxylic acid (**S2**) for 2 steps, **^{1}H NMR (500 MHz, $CDCl_3$)**: δ 4.44-4.40 (m, 3H), 4.32-4.28 (m, 3H). These NMR data are in accordance with the literature data.

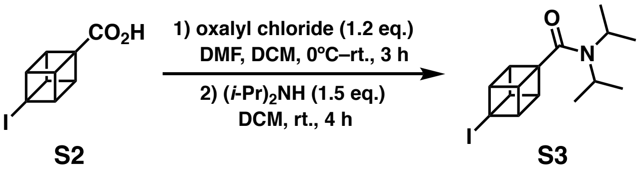

S2 S3

4-Iodo-*N*,*N*-diisopropylcubane-1-carboxamide (S3)

To a two-necked round bottom-flask equipped with a magnetic stir bar were added 4-iodocubanecarboxylic acid (1.47 g, 5.38 mmol, 1.00 eq.), DMF (10 drops) and CH_2Cl_2 (16.4 mL) under an argon atmosphere. The mixture was cooled to 0 °C, and oxalyl chloride (552 μL, 6.46 mmol, 1.20 eq.) was added in few portions. The reaction was allowed to stir at room temperature for 3 h. Then, all the volatiles were removed under reduced pressure, and the crude acid chloride was dissolved in CH_2Cl_2 (7.0 mL). In a separate flame-dried flask under an argon atmosphere, a solution of diisopropylamine (846 μL, 6.46 mmol, 1.20 eq.) and triethylamine (980 μL, 7.01 mmol, 1.30 eq.) in CH_2Cl_2 (4.7 mL) was cooled to 0 °C and, this solution was added dropwise to the acid chloride solution *via* cannula. The mixture was then stirred at room temperature for 3 h, washed successively two times with 1N HCl and brine. The organic layer was dried over $MgSO_4$, filtered and evaporated under reduced pressure. The product was purified by silica gel flash column chromatography (eluent: AcOEt/n-hexane). 92% isolated yield (white solid).

4-Iodo-*N*,*N*-diisopropylcubane-1-carboxamide (S3):

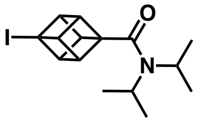

A white solid: **^{1}H NMR (500 MHz, CDCl$_3$)**: δ 4.34-4.31 (m, 3H), 4.28-4.25 (m, 3H), 3.38 (sep, *J* = 6.7 Hz, 1H), 3.29 (sep, *J* = 6.7 Hz, 1H), 1.41 (d, *J* = 6.7 Hz, 6H), 1.19 (d, *J* = 6.7 Hz, 6H).

^{13}C NMR (125 MHz, CDCl$_3$): δ 169.9, 59.9, 54.3, 50.3, 48.5, 46.1, 36.1, 21.1, 20.6.

HRMS (ESI (+)) *m/z* calcd for $C_{15}H_{20}INNaO^+$ $[M+Na]^+$ 380.0482, found 380.0480.

IR (neat) 2993, 2963, 1621, 1440, 1342, 1213, 1028, 820, 619, 431 cm^{-1}.

mp 114-115 ºC.

3-2. Characterization of the products

9-(4,4,5,5-Tetramethyl-1,3,2-dioxaborolane)triptycene (1a):

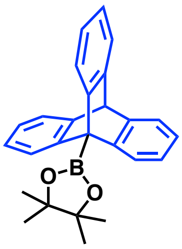

A white powder: **^{1}H NMR (500 MHz, CDCl$_3$)**: δ 7.79-7.76 (m, 3H), 7.35-7.32 (m, 3H), 6.98-6.93 (m, 6H), 5.32 (s, 1H), 1.61 (s, 12H).
^{13}C NMR (125 MHz, CDCl$_3$): δ 147.0, 146.6, 125.0, 124.9 (overlapped), 123.6, 84.4, 55.1, 25.7. The carbon directly attached to the boron atom was not detected, probably due to quadropolar relaxation.
HRMS (APCI (+)) *m/z* calcd for $C_{26}H_{26}BO_2^+$ [(M+H)$^+$] 381.2020, found 381.2019.
IR (neat) 3057, 2991, 2979, 1360, 1165, 1138, 987, 850, 741, 620 cm^{-1}.
mp 228-230 ºC.

9-(4-Methoxycarbonyl)phenyltriptycene (3):

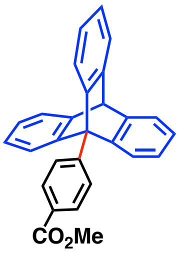

Compound (**3**) was prepared according to **Representative Procedure 1**, using methyl 4-iodobenzoate (144.4 mg, 0.55 mmol), CuI as a copper source, and **L1** as a ligand. The reaction was carried out at room temperature, stirred at 80 ºC for 4 h. The obtained material was purified by silica gel flash column chromatography (eluent: AcOEt/n-hexane) to give the desired compound **1** in 83% isolated yield.

A white powder: **^{1}H NMR (500 MHz, CDCl$_3$)**: δ 8.32 (d, *J* = 8.6 Hz, 2H), 8.21 (d, *J* = 8.6 Hz, 2H), 7.43 (dd, *J* = 7.5, 1.1 Hz, 3H), 7.19 (dd, *J* = 7.5, 1.1 Hz, 3H), 7.01 (ddd, *J* = 7.5, 7.5, 1.1 Hz, 3H), 6.94 (ddd, *J* = 7.5, 7.5, 1.1 Hz, 3H), 5.42 (s, 1H), 4.01 (s, 3H).
^{13}C NMR (125 MHz, CDCl$_3$): δ 167.1, 146.8, 146.2, 141.9, 131.7, 129.8, 129.2, 125.4, 124.8, 124.3, 123.8, 60.4, 55.2, 52.3.
HRMS (ESI (+)) *m/z* calcd for $C_{28}H_{20}NaO_2^+$ [(M+Na)$^+$] 411.1356, found 411.1372.
IR (neat) 3067, 1717, 1605, 1559, 1451, 1435, 1275, 855, 771, 743, 716 cm^{-1}.
mp 268-270 ºC.

9-(4-Trifluoromethyl)phenyltriptycene (4):

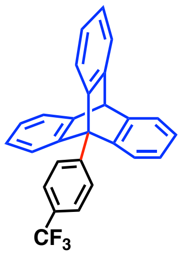

Compound (**4**) was prepared according to **Representative Procedure 1**, using 1-iodo-4-(trifluoromethyl)benzene (150.8 mg, 0.55 mmol), CuI a as copper source, and **L1** as a ligand. The reaction was carried out at 100 ºC for 4 h. The obtained material was purified by silica gel flash column chromatography (eluent: AcOEt/n-hexane) to give the desired compound **4** in 83% isolated yield.

A white powder: **^{1}H (500 MHz, CDCl$_3$)**: δ 8.26 (d, J = 8.0 Hz, 2H), 7.92 (d, J = 8.0 Hz, 2H), 7.44 (dd, J = 7.5, 1.1 Hz, 3H), 7.18 (dd, J = 7.5, 1.1 Hz, 3H), 7.02 (ddd, J = 7.5, 7.5, 1.1 Hz, 3H), 6.95 (ddd, J =7.5, 7.5, 1.1 Hz, 3H), 5.43 (s, 1H**)**.

^{13}C NMR (125 MHz, CDCl$_3$): δ 146.8, 146.1, 140.8, 132.0, 129.7 (q, $^2J_{C–F}$ = 32.3 Hz) 125.5, 125.5, 124.8, 124.4 (q, $^1J_{C–F}$ = 272.3 Hz), 124.2 123.9, 60.2, 55.2.

^{19}F NMR (470 MHz, CDCl$_3$): δ –62.3 (s, 3F).

HRMS (APCI (+)) *m/z* calcd for $C_{27}H_{18}F_3^+$ [(M+H)$^+$] 399.1355, found 399.1353.

IR (neat) 3070, 2954, 1620, 1455, 1326, 1111, 754, 750, 639, 493 cm^{-1}.

mp 263-266 ºC.

9-(4-Cyano)phenyltriptycene (5):

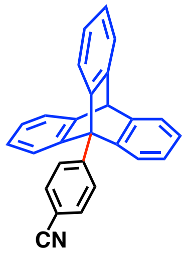

Compound (**5**) was prepared according to **Representative Procedure 1**, using 4-iodobenzonitrile (126.2 mg, 0.55 mmol), CuI as a copper source, and **L1** as a ligand. The reaction was carried out at 100 ºC for 4 h. The obtained material was purified by silica gel flash column chromatography (eluent: AcOEt/n-hexane) to give the desired compound **5** in 83% isolated yield.

A white powder: **^{1}H NMR (500 MHz, CDCl$_3$)**: δ 8.25 (d, J = 8.0 Hz, 2H), 7.94 (d, J = 8.0 Hz, 2H), 7.45 (dd, J = 7.5, 1.1 Hz, 3H), 7.13 (dd, J = 7.5, 1.1 Hz, 3H), 7.02 (ddd, J = 7.5, 7.5, 1.1 Hz, 3H), 6.95 (ddd, J = 7.5, 7.5, 1.1 Hz, 3H), 5.44 (s, 1H).

^{13}C NMR (125 MHz, CDCl$_3$): δ 146.7, 145.7, 142.3, 132.3, 125.6, 124.8, 124.0, (overlapped), 123.7, 118.9, 111.4, 60.3, 55.1.

HRMS (ESI (+)) *m/z* calcd for $C_{54}H_{34}N_2Na^+$ [(2M+Na)$^+$] 733.2614, found 733.2621.

IR (neat) 3071, 2967, 2231, 1455, 748, 732, 636, 567, 499, 475 cm^{-1}.

mp 274-275 ºC.

1-{4-(9-Triptycenyl)phenyl}ethan-1-one (6):

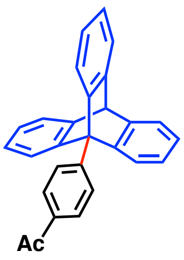

Compound (**6**) was prepared according to **Representative Procedure 1**, using 1-(4-iodophenyl)ethan-1-one (135.4 mg, 0.55 mmol) and CuI as a copper source, and **L1** as a ligand. The reaction was carried out at 80 ºC for 24 h. The obtained material was purified by silica gel flash column chromatography (eluent: AcOEt/n-hexane) to give the desired compound **6** in 81% isolated yield.

A yellow powder: **^{1}H NMR (500 MHz, C_6D_6)**: δ 7.97 (d, *J* = 9.0 Hz, 2H), 7.91 (d, *J* = 9.0 Hz, 2H), 7.26 (dd, *J* = 7.0, 1.0 Hz, 3H), 7.22 (dd, *J* = 7.5, 1.0 Hz, 3H), 6.86 (ddd, *J* = 7.5, 7.0, 1.0 Hz, 3H), 6.80 (ddd, *J* = 7.5, 7.5, 1.0 Hz, 3H), 5.24 (s, 1H), 2.27 (s, 3H).

^{13}C NMR (125 MHz, C_6D_6): δ 196.1, 147.3, 146.8, 141.9, 136.4, 131.9, 128.6, 125.6, 125.0, 124.7, 124.0, 60.8, 55.8, 26.2.

HRMS (ESI (+)) *m/z* calcd for $C_{56}H_{40}NaO_2^+$ [(2M+Na)$^+$] 767.2921, found 767.2947.

IR (neat) 3070, 2961, 1725, 1681, 1455, 1266, 944, 757, 638, 474 cm^{-1}.

mp 271-274 ºC.

9-Phenyltriptycene (7):

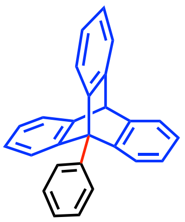

Compound (**7**) was prepared according to **Representative Procedure 1**, using iodobenzene (112.2 mg, 0.55 mmol) and CuI as a copper source, and **L1** as a ligand. The reaction was carried out at room temperature, stirred at 100 ºC and the desired product was obtained in 77% ^{1}H NMR yield with this procedure. The obtained material was purified by silica gel flash column chromatography (eluent: AcOEt/n-hexane) to give the desired compound **7** in 53% isolated yield (The lowered yield should be due to the poor solubility of **7**).

A white powder: **^{1}H NMR (500 MHz, $CDCl_3$)**: δ 8.12 (d, *J* = 8.0 Hz, 2H), 7.64 (dd, *J* = 8.0, 7.5 Hz, 2H), 7.53 (dd, *J* = 7.5, 7.5 Hz, 1H), 7.42 (dd, *J* = 7.5, 1.1 Hz, 3H), 7.25 (d, *J* = 7.5, 1.1 Hz, 3H), 6.99 (ddd, *J* = 7.5, 7.5, 1.1 Hz, 3H), 6.93 (ddd, *J* = 7.5, 7.5, 1.1 Hz, 3H), 5.42 (s, 1H).

^{13}**C NMR (125 MHz, CDCl$_3$)**: δ 146.9, 146.8, 136.5, 131.6, 128.6, 127.3, 125.2, 124.6, 124.6, 123.7, 60.2, 55.3.

HRMS (ESI (+)) *m/z* calcd for $C_{52}H_{36}Ag^+$ [2M+Ag]$^+$ 767.1862, found 767.1863.

IR (neat) 3057, 2950, 1494, 1455, 1141, 758, 737, 713, 638, 629 cm^{-1}.

mp 150-153 ºC.

9-(4-Methoxyphenyl)triptycene (8):

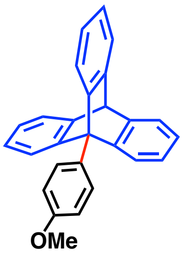

Compound (**8**) was prepared according to **Representative Procedure 1**, using 1-iodo-4-methoxybenzene (128.7 mg, 0.55 mmol), CuI as a copper source, and **L1** as a ligand. The reaction was carried out at 80 ºC for 18 h. The obtained material was purified by silica gel flash column chromatography (eluent: AcOEt/n-hexane) to give the desired compound **8** in 82% isolated yield.

A white powder: ^{1}**H NMR (500 MHz, CDCl$_3$)**: δ 8.03 (d, *J* = 8.6 Hz, 2H), 7.41 (d, *J* = 6.9 Hz, 3H), 7.26 (d, *J* = 6.9 Hz, 3H), 7.18 (d, *J* = 8.6 Hz, 2H), 6.99 (dd, *J* = 6.9, 6.9 Hz, 3H), 6.93 (dd, *J* = 6.9, 6.9 Hz, 3H), 5.41 (s, 1H), 3.97 (s, 1H), 1.54 (s, 3H).

^{13}**C NMR (125 MHz, CDCl$_3$)**: δ 158.5, 147.1, 146.8, 132.8, 128.3, 125.1, 124.6, 124.5, 123.8, 113.8, 59.7, 55.5, 55.2.

HRMS (ESI (+)) *m/z* calcd for $C_{27}H_{20}NaO^+$ [(M+Na)$^+$] 383.1406, found 383.1438.

IR (neat) 3060, 2954, 1512, 1450, 1252, 1031, 754, 745, 636, 628 cm^{-1}.

mp 242-244 ºC.

9-(2-Methoxycarbonylphenyl)triptycene (9):

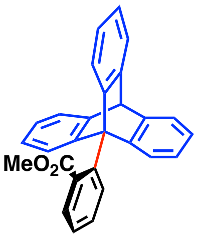

Compound (**9**) was prepared according to **Representative Procedure 1**, using methyl 2-iodobenzoate (145.5 mg, 0.56 mmol), CuCl as a copper source, and **L2** (SPhos) as a ligand. The reaction was carried out at 140 ºC for 24 h. The obtained material was purified by silica gel flash column chromatography (eluent: AcOEt/n-hexane) to give the desired compound **9** in 67% isolated yield.

A white powder: ^{1}**H NMR (500 MHz, CDCl$_3$)**: δ 8.30 (d, *J* = 8.0 Hz, 1H), 8.03 (d, *J* = 7.5 Hz, 1H), 7.75 (dd, *J* = 7.5, 7.5 Hz, 1H), 7.60 (dd, *J* = 8.0, 7.5 Hz, 1H), 7.37 (d, *J* = 7.0 Hz,

3H), 7.15 (br-s, 3H), 6.95 (dd, $J = 7.5, 7.0$ Hz, 3H), 6.90 (dd, $J = 7.5, 7.5$ Hz, 3H), 5.33 (s, 1H), 2.83 (s, 3H).

^{13}C NMR (125 MHz, CDCl$_3$): δ 169.9, 147.2, 145.9, 136.0, 135.2, 133.2, 131.6, 130.8, 127.6, 125.0, 124.5, 124.3, 123.5, 61.3, 55.4, 51.2.

HRMS (ESI (+)) *m/z* calcd for $C_{28}H_{20}O_2$ [(M+H)$^+$] 320.9553, found 320.9549.

IR (neat) 3064, 2947, 1725, 1455, 1248, 1080, 744, 727, 639, 509 cm^{-1}.

mp 265-268 ºC.

9-(2-Methoxyphenyl)triptycene (10):

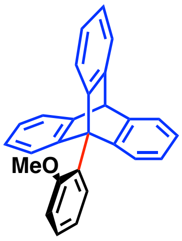

Compound (**10**) was prepared according to **Representative Procedure 1**, using 1-iodo-2-methoxybenzene (129.6 mg, 0.55 mmol), CuCl as a copper source, and **L2** (SPhos) as a ligand. The reaction was carried out at 140 ºC for 24 h. The obtained material was purified by silica gel flash column chromatography (eluent: AcOEt/n-hexane) to give the desired compound **10** in 31% isolated yield.

A white powder: **^{1}H NMR (500 MHz, CDCl$_3$)**: δ 8.39 (d, $J = 7.5$ Hz, 1H), 7.55 (dd, $J = 8.0, 7.5$ Hz, 1H), 7.37 (d, $J = 6.9$ Hz, 3H), 7.26 (dd, $J = 8.0, 7.5$ Hz, 1H), 7.21 (d, $J = 7.5$ Hz, 1H), 7.15 (d, $J = 7.5$ Hz, 3H), 6.96 (dd, $J = 7.5, 6.9$ Hz, 3H), 6.89 (dd, $J = 7.5, 7.5$ Hz, 3H), 5.35 (s, 1H), 3.15 (s, 3H).

^{13}C NMR (125 MHz, CDCl$_3$): δ 159.3, 146.3, 132.1, 129.2, 125.0, 124.9, 124.7, 124.3, 123.4, 120.5, 112.6, 59.4, 55.4, 54.5. (One peak is missing due to the overlap in the aromatic region.)

HRMS (ESI (+)) *m/z* calcd for $C_{27}H_{20}NaO^+$ [(M+Na)$^+$] 383.1406, found 383.1427.

IR (neat) 3007, 2831, 1488, 1455, 1246, 1138, 1027, 770, 736, 640 cm^{-1}.

mp 219-221 ºC.

9-Ferrocenyltriptycene (11):

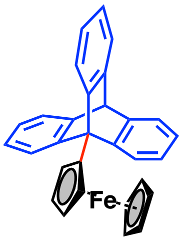

Compound (**11**) was prepared according to **Representative Procedure 1**, using bromoferrocene (145.5 mg, 0.55 mmol), CuCl as a copper source, and **L2** (SPhos) as a ligand. The reaction was carried out at 140 ºC for 24 h. The desired product was obtained in 66% ^{1}H NMR yield with this procedure. The obtained material was purified by silica gel flash column chromatography (eluent: AcOEt/n-hexane) to give the desired compound **11** in 33% isolated yield (The lowered yield should be due to the poor solubility of **11**).

A yellow powder: **^{1}H NMR (500 MHz, CDCl$_3$)**: δ 8.58 (d, J = 7.5 Hz, 2H), 7.40 (d, J = 6.9 Hz, 2H), 7.35 (d, J = 7.5 Hz, 1H), 7.09 (dd, J = 7.5, 7.5 Hz, 2H), 7.01 (dd, J = 7.5, 6.9 Hz, 2H), 6.95 (dd, J = 7.5, 7.5 Hz, 1H), 6.78 (dd, J = 7.5, 7.5 Hz, 1H), 6.16 (d, J = 7.5 Hz, 1H), 5.37 (s, 1H), 4.62 (dd, J = 1.7, 1.7 Hz, 2H), 4.59 (dd, J = 1.7, 1.7 Hz, 2H), 4.38 (s, 5H).

All spectral data are consistent with those previously reported.[S13]

1-{5-(9-Triptycenyl)thiophen-2-yl}ethan-1-one (12):

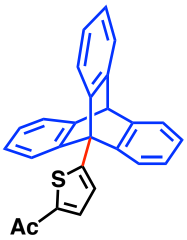

Compound (**12**) was prepared according to **Representative Procedure 1**, using 1-(5-bromothiophen-2-yl)ethan-1-one (112.7 mg, 0.55 mmol), CuCl as a copper source, and **L1** as a ligand. The reaction was carried out at 100 ºC for 24 h. The obtained material was purified by silica gel flash column chromatography (eluent: AcOEt/n-hexane) to give the desired compound **12** in 66% isolated yield.

A white powder: **^{1}H NMR (500 MHz, CDCl$_3$)**: δ 7.97 (d, J = 4.0 Hz, 1H), 7.58 (d, J = 4.0 Hz, 1H), 7.44 (dd, J = 7.5, 1.4 Hz, 3H), 7.42 (dd, J = 7.5, 1.1 Hz, 3H), 7.04 (ddd, J = 7.5, 7.5, 1.1 Hz, 3H), 6.99 (ddd, J = 7.5, 7.5, 1.4 Hz, 3H), 5.44 (s, 1H), 2.72 (s, 3H).

^{13}C NMR (125 MHz, CDCl$_3$): δ 191.1, 147.9, 146.1, 146.0, 143.6, 132.9, 132.1, 126.0, 143.6, 132.9, 132.1, 126.0, 125.3, 124.0, 123.6, 59.5, 54.9, 27.3.

HRMS (ESI (+)) *m/z* calcd for $C_{52}H_{36}NaO_2S_2^+$ [$(2M+Na)^+$] 779.2049, found 779.2072.

IR (neat) 3067, 2962, 1651, 1439, 1272, 1034, 759, 739, 613, 494 cm^{-1}.

mp 276-278 ºC.

Ethyl 5-(9-triptycenyl)thiophene-2-carboxylate (13):

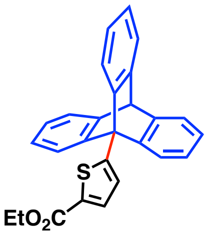

Compound (**13**) was prepared according to **Representative Procedure 1**, using ethyl 5-bromothiophene-2-carboxylate (145.2 mg, 0.61 mmol), CuCl as a copper source, and **L1** as a ligand. The reaction was carried out at 100 ºC for 24 h. The obtained material was purified by silica gel flash column chromatography (eluent: AcOEt/n-hexane) to give the desired compound **13** in 60% isolated yield.

A white powder: **^{1}H NMR (500 MHz, $CDCl_3$)**: δ 8.07 (d, J = 4.0 Hz, 1H), 7.56 (d, J = 4.0 Hz, 1H), 7.42 (m, 6H), 7.04 (ddd, J = 7.5, 7.5, 1.5 Hz, 3H), 6.99 (ddd, J = 7.5, 7.5, 1.5 Hz, 3H), 5.44 (s, 1H), 4.42 (q, J = 7.0 Hz, 2H), 1.46 (t, J = 7.0 Hz, 3H).

^{13}C NMR (125 MHz, $CDCl_3$): δ 162.4, 146.0, 145.9, 133.2, 132.8, 132.3, 125.8, 125.1 (overlapped), 123.8, 123.4, 61.5, 59.3, 54.8, 14.6.

HRMS (ESI (+)) *m/z* calcd for $C_{54}H_{40}NaO_4S_2^+$ [$(2M+Na)^+$] 839.2260, found 839.2318.

IR (neat) 3073, 2954, 1716, 1456, 1443, 1248, 1227, 1091, 737, 646 cm^{-1}.

mp 215-217 ºC.

2-(9-Triptycenyl)pyridine (14):

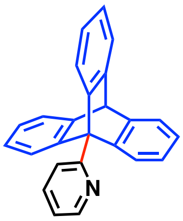

Compound (**14**) was prepared according to **Representative Procedure 1**, using 2-iodopyridine (114.8 mg, 0.56 mmol), CuCl as a copper source, and **L1** as a ligand. The reaction was carried out at 140 ºC for 48 h. The obtained material was purified by silica gel flash column chromatography (eluent: AcOEt/n-hexane) to give the desired compound **14** in 64% isolated yield.

A white powder: **^{1}H NMR (500 MHz, $CDCl_3$)**: δ 8.97-8.98 (m, 1H), 7.91 (ddd, J = 8.0, 7.5, 1.9 Hz, 1H), 7.75 (d, J = 8.0 Hz, 1H), 7.61 (dd, J = 7.5, 1.5 Hz, 3H), 7.41-7.46 (m, 4H), 7.00 (ddd, J = 7.5, 7.5, 1.5 Hz, 3H), 6.94 (ddd, J = 7.5, 7.5, 1.5 Hz, 3H), 5.43 (s, 1H).

^{13}C NMR (125 MHz, $CDCl_3$): δ 158.6, 149.0, 146.6, 135.4, 128.2, 125.2, 125.0 (overlapped), 124.7, 123.5, 122.1, 60.6, 55.1.

HRMS (ESI (+)) *m/z* calcd for $C_{50}H_{34}N_2Na^+$ [$(2M+Na)^+$] 685.2614, found 685.2619.

IR (neat) 3068, 2952, 1586, 1455, 1421, 741, 638, 618, 494, 477 cm^{-1}.

mp 242-244 ºC.

3-(9-Triptycenyl)pyridine (15):

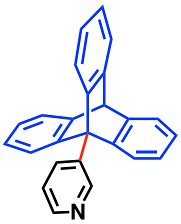

Compound (**15**) was prepared according to **Representative Procedure 1**, using 3-iodopyridine (112.8 mg, 0.55 mmol), CuCl as a copper source, and **L1** as a ligand. The reaction was carried out at 140 ºC for 48 h. The obtained material was purified by silica gel flash column chromatography (eluent: AcOEt/n-hexane) to give the desired compound **15** in 45% isolated yield.

A white powder: **^{1}H NMR (500 MHz, CDCl$_3$)**: δ 9.40 (d, *J* = 2.5 Hz, 1H), 8.80 (dd, *J* = 5.0, 1.5 Hz, 1H), 8.44 (ddd, *J* = 8.0, 2.5, 1.5 Hz, 1H), 7.61 (dd, *J* = 8.0, 5.0 Hz, 1H), 7.45 (dd, *J* = 7.5, 1.1 Hz, 3H), 7.18 (dd, *J* = 7.5, 1.5 Hz, 3H), 7.02 (ddd, *J* = 7.5, 7.5, 1.1 Hz, 3H), 6.95 (ddd, *J* = 7.5, 7.5, 1.5 Hz, 3H), 5.45 (s, 1H).

^{13}C NMR (125 MHz, CDCl$_3$): δ 152.7, 148.5, 146.6, 145.9, 139.1, 132.5, 125.5, 124.8, 124.0, 124.0, 123.3, 58.5, 55.1.

HRMS (ESI (+)) *m/z* calcd for $C_{25}H_{18}N^+$ $[M+H]^+$ 332.1434, found 332.1446.

IR (neat) 3066, 3038, 1681, 1455, 741, 707, 638, 605, 499, 475 cm^{-1}.

mp 258-261 ºC.

4-(9-Triptycenyl)pyridine (16):

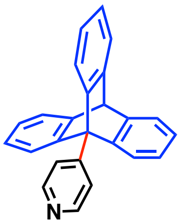

Compound (**16**) was prepared according to **Representative Procedure 1**, using 4-iodopyridine (112.8 mg, 0.55 mmol) and CuCl as a copper source, and **L1** as a ligand. The reaction was carried out at 140 ºC for 48 h. The obtained material was purified by silica gel flash column chromatography (eluent: AcOEt/n-hexane) to give the desired compound **16** in 73% isolated yield.

A white powder: **^{1}H NMR (500 MHz, CDCl$_3$)**: δ 9.12 (br-s, 2H), 8.10 (br-s, 2H), 7.45 (dd, *J* = 7.5, 1.5 Hz, 3H), 7.20 (dd, *J* = 7.5, 1.1 Hz, 3H), 7.04 (ddd, *J* = 7.5, 7.5, 1.1 Hz, 3H), 6.96 (ddd, *J* = 7.5, 7.5, 1.5 Hz, 3H), 5.45 (s, 1H).

^{13}C NMR (125 MHz, CD$_2$Cl$_2$): δ 150.7, 147.0, 145.7, 145.4, 126.7, 125.8, 125.1, 124.3, 124.2, 60.0, 55.2.

HRMS (ESI (+)) *m/z* calcd for $C_{50}H_{34}N_2Na^+$ $[(2M+Na)^+]$ 685.2614, found 685.2628.

IR (neat) 3068, 2849, 1681, 1455, 749, 739, 638, 628, 486, 474 cm^{-1}.

mp 290-293 ºC.

2-(9-Triptycenyl)quinoline (17):

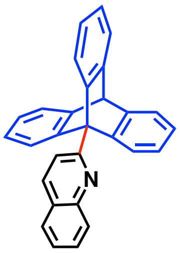

Compound (**17**) was prepared according to **Representative Procedure 1**, using 2-bromoquinoline (114.4 mg, 0.55 mmol) and CuCl as a copper source, and **L1** as a ligand. The reaction was carried out at 100 ºC for 24 h. The obtained material was purified by silica gel flash column chromatography (eluent: AcOEt/n-hexane) to give the desired compound **17** in 74% isolated yield.

A yellow powder: **^{1}H NMR (500 MHz, CDCl$_3$)**: δ 8.39 (d, *J* = 8.3 Hz, 1H), 8.31 (d, *J* = 8.3 Hz, 1H), 7.98 (dd, *J* = 8.5, 1.4 Hz, 1H), 7.82 (ddd, *J* = 8.5, 6.9, 1.4 Hz, 1H), 7.79 (dd, *J* = 7.8, 1.1 Hz, 3H), 7.74 (dd, *J* = 8.5, 1.4 Hz, 1H), 7.67 (ddd, *J* = 8.5, 6.9, 1.4 Hz, 1H), 7.44 (dd, *J* = 7.5, 1.4 Hz, 3H), 7.02 (ddd, *J* = 7.5, 7.5, 1.1 Hz, 3H), 6.95 (ddd, *J* = 7.8, 7.5, 1.4 Hz, 3H), 5.46 (s, 1H).

^{13}C NMR (125 MHz, CDCl$_3$): δ 159.3, 147.8, 146.7, 146.5, 134.6, 130.1, 129.4, 127.6, 127.1, 126.8, 126.1, 125.4, 125.3, 124.8, 123.5, 60.7, 55.2.

HRMS (ESI (+)) *m/z* calcd for $C_{29}H_{20}N^+$ [(M+H)$^+$] 382.1590, found 382.1605.

IR (neat) 3060, 3030, 1602, 1500, 1455, 1423, 747, 620, 489, 478 cm^{-1}.

mp over 300 ºC.

3-(9-Triptycenyl)quinoline (18):

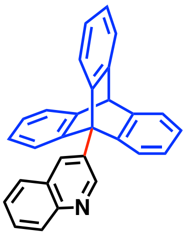

Compound (**18**) was prepared according to **Representative Procedure 1**, using 3-bromoquinoline (115.8 mg, 0.56 mmol), CuCl as a copper source, and **L1** as a ligand. The reaction was carried out at 140 ºC for 24 h. The obtained material was purified by silica gel flash column chromatography (eluent: AcOEt/n-hexane) to give the desired compound **18** in 90% isolated yield.

A yellow powder: **^{1}H NMR (500 MHz, CDCl$_3$)**: δ 9.52 (d, *J* = 2.3 Hz, 1H), 9.06 (d, *J* = 2.3 Hz, 1H), 8.28 (dd, *J* = 8.6, 1.2 Hz, 1H), 8.00 (dd, *J* = 8.0, 1.7 Hz, 1H), 7.86 (ddd, *J* = 8.6, 8.0, 1.7 Hz, 1H), 7.68 (ddd, *J* = 8.0, 8.0, 1.2 Hz, 3H), 7.48 (dd, *J* = 7.5, 1.2 Hz, 3H),

7.25 (dd, J = 7.5, 1.2 Hz, 3H), 7.06 (ddd, J = 7.5, 7.5, 1.2 Hz, 3H), 6.97 (ddd, J = 7.5, 7.5, 1.2 Hz, 3H), 5.49 (s, 1H).

^{13}C NMR (125 MHz, CDCl$_3$): δ 153.9, 146.9, 146.6, 146.0, 138.1, 130.1, 129.9, 129.3, 128.3, 127.8, 127.1, 125.6, 124.9, 124.1, 124.0, 58.5, 55.1.

HRMS (ESI (+)) *m/z* calcd for $C_{29}H_{20}N^+$ [M+H]$^+$ 382.1590, found 382.1607.

IR (neat) 3061, 1455, 862, 788, 760, 743, 669, 638, 632, 566 cm^{-1}.

mp 290-291 ºC.

9-(Phenylethynyl)triptycene (19):

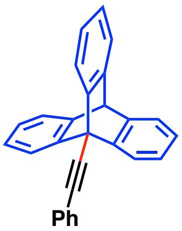

Compound (**19**) was prepared according to **Representative Procedure 1**, using (iodoethynyl)benzene (125.2 mg, 0.55 mmol) and CuI as a copper source, and **L1** as a ligand. The reaction was carried out at 100 ºC for 4 h. The obtained material was purified by silica gel flash column chromatography (eluent: AcOEt/n-hexane) and by GPC (eluent: CHCl$_3$) to give the desired compound **19** in 24% isolated yield.

A white powder: **^{1}H NMR (500 MHz, CDCl$_3$)**: δ 7.84-7.81 (m, 5H), 7.46 (dd, J = 7.2, 1.7 Hz, 3H), 7.40 (dd, J = 7.2, 1.7 Hz, 3H), 7.08-7.02 (m, 6H), 5.44 (s, 1H).

^{13}C NMR (125 MHz, CDCl$_3$): δ 144.5, 132.2, 128.8, 128.6, 125.8, 125.3, 124.8, 123.5, 123.1, 122.6, 92.8, 83.8, 53.6, 53.3.

HRMS (ESI (+)) *m/z* calcd for $C_{56}H_{36}Ag^+$ [2M+Ag]$^+$ 815.1862, found 815.1871.

IR (neat) 3064, 1455, 1441, 755, 750, 737, 639, 487, 477, 419 cm^{-1}.

mp 286-288 ºC.

Methyl (9-triptycenyl)acetate (20):

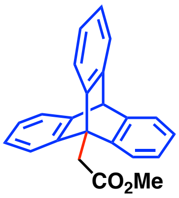

Compound (**20**) was prepared according to **Representative Procedure 1**, using methyl 2-bromoacetate (85.2 mg, 0.56 mmol) and CuI as a copper source, nickel(II) acetylacetonate (Ni(acac)$_2$) was used instead of Pd(OAc)$_2$ as a metal catalyst, and **L2** (SPhos**)** as a ligand. The reaction was carried out at 80 ºC for 53 h. The obtained material was purified by silica gel flash column chromatography (eluent: AcOEt/n-hexane) to give the desired compound **20** in 76% isolated yield.

A white powder: **^{1}H NMR (500 MHz, CDCl$_3$)**: δ 7.39-7.36 (m, 3H), 7.23-7.20 (m, 3H), 7.01-6.97 (m, 6H), 5.39 (s, 1H), 3.99 (s, 2H), 3.84 (s, 3H).

^{13}C NMR (125 MHz, CDCl$_3$): δ 171.6, 146.2, 145.1, 125.3, 124.8, 123.6, 121.9, 54.4, 52.1, 51.5, 32.9.

IR (neat) 3073, 3020, 2947, 1747, 1455, 1176, 987, 743, 633, 482 cm^{-1}.

HRMS (ESI (+)) *m/z* calcd for $C_{23}H_{19}O_2^+$ [M+H]$^+$ 327.1380, found 327.1378.

mp 187-189 ºC.

All spectral data are consistent with those previously reported. [S14]

Methyl *p*-adamantylbenzoate (21):

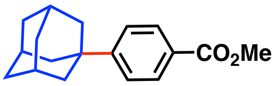

Compound (**21**) was prepared according to **Representative Procedure 2**.

A white solid: **^{1}H NMR (300 MHz, CDCl$_3$)**: δ 7.98 (d, J = 7.0 Hz, 2H), 7.43 (d, J = 7.0 Hz, 2H), 3.90 (s, 3H), 2.11 (br-s, 3H), 1.92 (s, 6H), 1.78 (m, 6H).

All spectral data are consistent with those previously reported.[S15]

5-(2,2-Dimethyl-1,3-dioxolan-4-yl)-2,2-dimethyltetrahydrofuro[2,3-*d*][1,3]dioxol-6-yl 4-(adamantan-1-yl)benzoate (22):

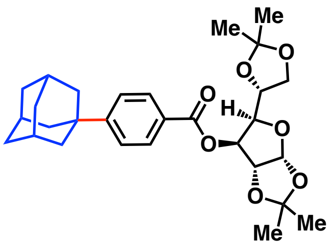

Compound (**22**) was prepared according to **Representative Procedure 2**, using 3-*O*-(4-iodobenzoyl)-1,2:5,6-di-*O*-isopropylidene-α-D-glucofuranose (269.7 mg, 0.55 mmol) and CuI as a copper source, and **L2** (SPhos) as a ligand. The reaction was carried out at 120 ºC for 48 h. The obtained material was purified by silica gel flash column chromatography (eluent: AcOEt/n-hexane) gave the desired compound **22** in 79% isolated yield.

A white powder: **^{1}H NMR (500 MHz, CDCl$_3$)**: δ 7.96 (dd, *J* = 8.5, 1.8 Hz, 2H), 7.44 (dd, *J* = 8.5, 1.8 Hz, 2H), 5.93 (d, *J* = 3.7 Hz, 1H), 5.48 (d, *J* = 2.8 Hz, 1H), 4.39-4.32 (m, 2H), 4.14-4.08 (m, 2H), 2.11 (br-s, 3H), 1.92 (d, *J* = 2.4 Hz, 6H), 1.82-1.74 (m, 6H), 1.55 (s, 3H), 1.41 (s, 3H), 1.31 (s, 3H), 1.27 (s, 3H).

^{13}C NMR (125 MHz, CDCl$_3$): δ 165.3, 157.5, 129.7, 126.8, 125.2, 112.4, 109.4, 105.2 83.5, 80.1, 76.5, 72.7, 67.3, 43.0, 36.8, 36.7, 28.9, 26.9 (overlapped), 26.3, 25.3.

HRMS (ESI (+)) *m/z* calcd for $C_{29}H_{38}NaO_7^+$ [$(M+Na)^+$] 521.2510, found 521.2557.

IR (neat) 2902, 2848, 1723, 1372, 1266, 1094, 1073, 1014, 843, 703 cm^{-1}.

mp over 300 ºC.

(8*R*,9*S*,13*S*,14*S*,17*S*)-2-(Adamantan-1-yl)-3,17-dimethoxy-13-methyl-7,8,9,11,12,13,14,15,16,17-decahydro-6*H* cyclopenta[*a*]phenanthrene (23):

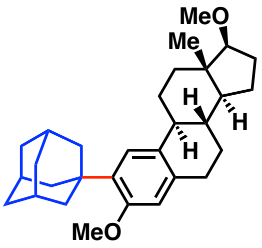

Compound (**23**) was prepared according to **Representative Procedure 2**, using (17*β*)-2-iodo-3,17-dimethoxyestra-1,3,5(10)-triene (234.6 mg, 0.55 mmol) and CuI as a copper source, and **L2** (SPhos) as a ligand. The reaction was carried out at 100 ºC for 24 h. The desired product was obtained in 70% ^{1}H NMR yield with this procedure. The obtained material was purified by silica gel flash column chromatography (eluent: AcOEt/n-hexane) gave the desired compound **23** in 54% isolated yield.

A white powder: **^{1}H NMR (500 MHz, $CDCl_3$)**: δ 7.15 (s, 1H), 6.58 (s, 1H), 3.79 (s, 3H), 3.37 (s, 3H), 3.31 (t, *J* = 8.5 Hz, 1H), 2.88-2.77 (m, 2H), 2.32 (ddd, *J* = 12.8, 7.0, 4.0 Hz, 1H), 2.19 (td, *J* = 12.8, 4.0 Hz, 1H), 2.08 (d, *J* = 2.7 Hz, 6H), 2.06-2.02 (m, 5H), 1.86 (ddt, *J* = 14.0, 4.5, 2.1 Hz, 1H), 1.75 (br-s, 6H), 1.68 (tdd, *J* = 14.0, 7.0, 3.0 Hz, 1H),1.55-1.46 (m, 4H), 1.42 (dt, *J* = 10.0, 2.4 Hz, 1H), 1.33 (tdd, *J* = 12.2, 9.5, 6.1 Hz, 1H), 1.21 (ddd, *J* = 12.5, 11.0, 7.0 Hz, 1H), 0.78 (s, 3H).

^{13}C NMR (125 MHz, $CDCl_3$): δ 156.7, 135.9, 135.0, 131.7, 123.8, 112.2, 90.9, 58.0, 55.1, 50.4, 44.4, 43.4, 40.9, 38.9, 38.2, 37.3, 37.0, 31.0, 29.5, 29.3, 29.2, 27.9, 27.4, 26.6, 23.2, 11.7.

HRMS (APCI (+)) *m/z* calcd for $C_{30}H_{43}O_2^+$ [$(M+H)^+$] 435.3258, found 435.3275.

IR (neat) 2904, 2883, 2866, 1501, 1463, 1230, 1122, 1102, 1041, 861 cm^{-1}.

mp 296-297 °C (decomp.).

4-(1,1-Dimethylethyl)benzoic acid methyl ester (24):

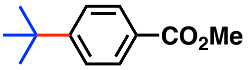

Compound (**24**) was prepared according to **Representative Procedure 3**.

Colorless oil: **^{1}H NMR (500 MHz, $CDCl_3$)**: δ 7.97 (d, *J* = 8.4 Hz, 2H), 7.45 (d, *J* = 8.4 Hz, 2H), 3.90 (s, 3H), 1.34 (s, 9H).

All spectral data are consistent with those previously reported.[S16]

Methyl 4-(adamantan-2-yl)benzoate (25):

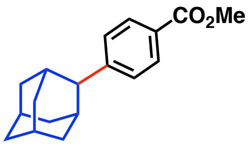

Compound (**25**) was prepared according to **Representative Procedure 2**, using methyl 4-iodobenzoate (144.3 mg, 0.55 mmol) and CuI as a copper source, and **L1** as a ligand. The reaction was carried out at 100 °C for 48 h. The obtained material was purified by silica gel flash column chromatography (eluent: AcOEt/n-hexane) gave the desired compound **25** in 88% isolated yield.

A white powder: **^{1}H NMR (500 MHz, $CDCl_3$)**: δ 7.98 (d, *J* = 8.5 Hz, 2H), 7.42 (d, *J* = 8.5 Hz, 2H), 3.90 (s, 3H), 3.03 (br-s, 1H), 2.49 (br-s, 2H), 2.02-1.93 (m, 5H), 1.79-1.77 (m, 5H), 1.59-1.564 (m, 1H).

^{13}C NMR (125 MHz, CDCl$_3$): δ 167.4, 150.3, 129.6, 127.2, 127.0, 52.0, 47.2, 39.1, 37.8, 32.1, 31.1, 28.0, 27.8.

HRMS (ESI (+)) *m/z* calcd for $C_{36}H_{44}NaO_4^+$ [(2M+Na)$^+$] 563.3132, found 563.3154.

IR (neat) 2903, 2846, 1720, 1605, 1434, 1272, 1228, 1103, 741, 503 cm^{-1}.

mp 141-143 ºC.

Methyl 2',4',6'-trimethyl-[1,1'-biphenyl]-4-carboxylate (26):

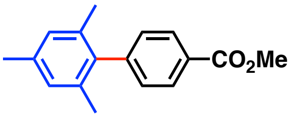

Compound (**26**) was prepared according to **Representative Procedure 4**, using 1-bromo-2,4,6-trimethylbenzene (99.5 mg, 0.50 mmol) and methyl 4-iodobenzoate (144.1 mg, 0.55 mmol), CuI as a copper source, and **L1** as a ligand. Compound (**26**) was also prepared using methyl 4-bromobenzoate (118.2 mg, 0.55 mmol) with the same procedure (and the purification with reverse-phase silica gel chromatography was needed), and the desired product was obtained in 77% yield.

A white powder: **^{1}H NMR (400 MHz, CDCl$_3$)**: δ 8.10 (d, J = 8.3 Hz, 2H), 7.22 (d, J = 8.3 Hz, 2H), 6.95 (s, 2H), 3.94 (s, 3H), 2.33 (s, 3H), 1.98 (s, 6H).

All spectral data are consistent with those previously reported.[S17]

Methyl 2',4',6'-trimethyl-[1,1'-biphenyl]-2-carboxylate (27):

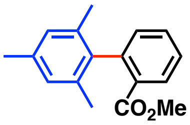

Compound (**27**) was prepared according to **Representative Procedure 4**, using 1-bromo-2,4,6-trimethylbenzene (112.3 mg, 0.56 mmol) and methyl 2-iodobenzoate (143.6 mg, 0.55 mmol), CuI as a copper source, and **L2** (SPhos) as a ligand. The reaction was carried out at 80 ºC (and the purification with reverse-phase silica gel chromatography was needed) and the desired product was obtained in 55% yield with this procedure.

A white powder: **^{1}H NMR (400 MHz, CDCl$_3$)**: δ 7.99 (d, J = 7.5 Hz, 1H), 7.55 (dd, J = 7.5, 7.5 Hz, 1H), 7.41 (dd, J = 7.5, 7.5 Hz, 1H), 7.14 (d, J = 7.5 Hz, 1H), 6.90 (s, 2H), 3.63 (s, 3H), 2.32 (s, 3H), 1.90 (s, 6H).

All spectral data are consistent with those previously reported.[S18]

4'-Methoxy-2,4,6-trimethyl-1,1'-biphenyl (28):

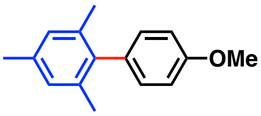

Compound (**28**) was prepared according to **Representative Procedure 4**, using 1-bromo-2,4,6-trimethylbenzene (99.7 mg, 0.50 mmol) and 1-iodo-4-methoxybenzene (128.7 mg, 0.55 mmol), CuI as a copper source, and **L1** as a ligand. The reaction was carried out at room temperature and the desired product was obtained in 96% yield with this procedure.

A white powder: **^{1}H NMR (400 MHz, $CDCl_3$)**: δ 7.05 (d, J = 6.7 Hz, 2H), 6.95 (d, J = 6.7 Hz, 2H), 6.93 (s, 2H), 3.85 (s, 3H), 2.32 (s, 3H), 2.01 (s, 6H).

All spectral data are consistent with those previously reported.[S19]

2'-Methoxy-2,4,6-trimethyl-1,1'-biphenyl (29):

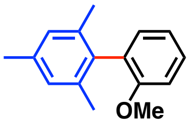

Compound (**29**) was prepared according to **Representative Procedure 4**, using 1-bromo-2,4,6-trimethylbenzene (101.5 mg, 0.51 mmol) and 1-iodo-2-methoxybenzene (131.0 mg, 0.56 mmol), CuI as a copper source, and **L1** as a ligand. The reaction was carried out at 80 ºC and the desired product was obtained in 91% yield with this procedure.

A white powder: **^{1}H NMR (400 MHz, $CDCl_3$)**: δ 7.32 (ddd, J = 8.7, 6.7, 2.8 Hz, 1H), 7.03-6.96 (m, 3H), 6.93 (s, 2H), 3.74 (s, 3H), 2.32 (s, 3H), 1.98 (s, 6H).

All spectral data are consistent with those previously reported.[S20]

2,2',4,4',6,6'-Hexamethyl-1,1'-biphenyl (30):

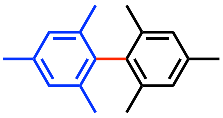

Compound (**30**) was prepared according to **Representative Procedure 4**, using 1-bromo-2,4,6-trimethylbenzene (for the preparation of copper reagent) (99.5 mg, 0.50 mmol) and 1-bromo-2,4,6-trimethylbenzene (as the electrophile) (104.9 mg, 0.55 mmol), CuI as a copper source, and **L2** (SPhos) as a ligand. The reaction was carried out at 80 ºC (and the purification with reverse-phase silica gel chromatography was needed) and the desired product was obtained in 71% ^{1}H NMR yield with this procedure.

A white powder: **^{1}H NMR (400 MHz, $CDCl_3$)**: δ 6.93 (s, 4H), 2.33 (s, 6H), 1.86 (s, 12H).

All spectral data are consistent with those previously reported.[S21]

9-Mesitylanthracene (31,33):

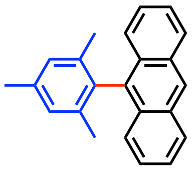

Compound (**31**) was prepared according to **Representative Procedure 4**, using 1-bromo-2,4,6-trimethylbenzene (106.3 mg, 0.53 mmol) and 9-bromoanthracene (141.5 mg, 0.55 mmol), CuI as a copper source, and **L2** (SPhos) as a ligand. The reaction was carried out at 120 ºC (and the purification with reverse-phase silica gel chromatography was needed) and the desired product was obtained in 31% yield with this procedure.

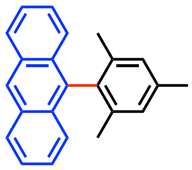

Compound (**33**) was prepared according to **Representative Procedure 4**, using 9-bromoanthracene (128.5 mg, 0.50 mmol) and 1-bromo-2,4,6-trimethylbenzene (109.4 mg, 0.55 mmol), CuI as a copper source, and **L2** (SPhos) as a ligand. The reaction was carried out at 120 ºC (and the purification with reverse-phase silica gel chromatography was needed) and the desired product was obtained in 41% yield with this procedure.

A yellow powder: **^{1}H NMR (500 MHz, $CDCl_3$)**: δ 8.43 (s, 1H), 8.05 (d, J = 8.5 Hz, 2H), 7.48-7.43 (m, 4H), 7.32 (ddd, J = 8.5, 6.5, 1.0 Hz, 2H), 7.08 (s, 2H), 2.45 (s, 3H), 1.70 (s, 6H).

All spectral data are consistent with those previously reported.[S22]

9-Mesitylacridine (32):

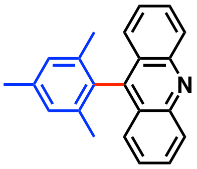

Compound (**32**) was prepared according to **Representative Procedure 4**, using 1-bromo-2,4,6-trimethylbenzene (102.5 mg, 0.51 mmol) and 9-bromoacridine (141.8 mg, 0.55 mmol), CuI as a copper source, and **L2** (SPhos) as a ligand. The reaction was carried out at 100 ºC (and the purification with reverse-phase silica gel chromatography was needed) and the desired product was obtained in 58% yield with this procedure.

A pale-yellow solid: **^{1}H NMR (500 MHz, $CDCl_3$)**: δ 8.29 (dd, J = 9.0, 1.5 Hz, 2H), 7.77 (ddd, J = 9.0, 6.0, 1.5 Hz, 2H), 7.52 (dd, J = 9.0, 1.5 Hz, 2H), 7.39 (ddd, J = 9.0, 6.0, 1.5 Hz, 2H), 7.09 (s, 2H), 2.45 (s, 3H), 1.71 (s, 6H).

All spectral data are consistent with those previously reported.[S23]

Methyl 2',4',6'-triisopropyl-[1,1'-biphenyl]-4-carboxylate (34):

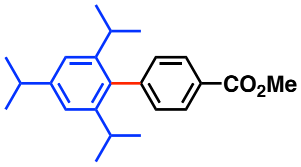

Compound (**34**) was prepared according to **Representative Procedure 4**, using 1-bromo-2,4,6-triisopropylbenzene (143.4 mg, 0.51 mmol) and methyl 4-iodobenzoate (144.2 mg, 0.55 mmol), CuI as a copper source, and **L1** as a ligand. The reaction was carried out at room temperature and the desired product was obtained in 85% isolated yield.

A white powder: **^{1}H NMR (500 MHz, $CDCl_3$)**: δ 8.07 (d, J = 8.0 Hz, 2H), 7.27 (d, J = 8.0 Hz, 2H), 7.06 (s, 2H), 3.94 (s, 3H), 2.93 (sep, J = 7.0 Hz, 1H), 2.52 (sep, J = 7.0 Hz, 2H), 1.30 (d, J = 7.0 Hz, 6H), 1.07 (d, J = 7.0 Hz, 12H).

^{13}C NMR (125 MHz, $CDCl_3$): δ 167.3, 148.5, 146.4, 146.3, 136.1, 130.1, 129.4, 128.6, 120.7, 52.2, 34.4, 30.4, 24.3, 24.2.

HRMS (ESI (+)) *m/z* calcd for $C_{23}H_{31}O_2^+$ $[M+H]^+$ 339.2319, found 339.2299.

IR (neat) 3003, 2958, 1716, 1608, 1444, 1277, 1110, 1101, 771, 714 cm^{-1}.

mp 99–101°C.

Methyl 2',4',6'-tri-*tert*-butyl-[1,1'-biphenyl]-4-carboxylate (35):

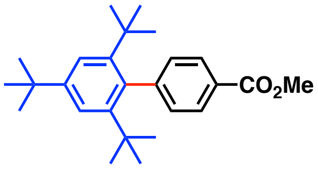

Compound (**35**) was prepared according to **Representative Procedure 4**, using 1-bromo-2,4,6-tri-*tert*-butylbenzene (162.5 mg, 0.50 mmol) and methyl 4-iodobenzoate (144.1 mg, 0.55 mmol), CuI as a copper source, and **L2** (SPhos) as a ligand. The reaction was carried out at 120 °C and the desired product was obtained in 34% yield with this procedure.

A white powder: **^{1}H NMR (500 MHz, $CDCl_3$)**: δ 7.96 (d, J = 8.0 Hz, 2H), 7.52 (s, 2H), 7.47 (d, J = 8.0 Hz, 2H), 3.94 (s, 3H), 1.36 (s, 9H), 1.05 (s, 18H).

^{13}C NMR (125 MHz, $CDCl_3$): δ 167.4, 148.8, 148.5, 147.9, 137.1, 134.3, 129.0, 127.3, 122.5, 52.2, 37.8, 35.1, 33.7, 31.6.

HRMS (ESI (+)) *m/z* calcd for $C_{52}H_{72}NaO_4^+$ $[2M+Na]^+$ 783.5323, found 783.5388.

IR (neat) 3014, 2950, 1716, 1283, 1273, 1114, 1102, 824, 781, 722 cm^{-1}.

mp 148-150 °C.

4-(*p*-Methylbenzoate)-1,1,7,7-tetraethyl-3,3,5,5-tetramethyl-1,2,3,5,6,7-hexahydro-*s*-indacene (36):

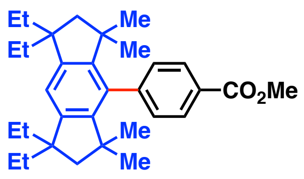

Compound (**36**) was prepared according to **Representative Procedure 4**, using 4-bromo-1,1,7,7-tetraethyl-1,2,3,5,6,7-hexahydro-3,3,5,5-tetramethyl-*s*-indacene (202.7 mg, 0.50 mmol) and methyl 4-iodobenzoate (144.1 mg, 0.55 mmol), CuCl as a copper source, and **L2** (SPhos) as a ligand. The reaction was carried out at 120 ºC and the desired product was obtained in 53% isolated yield.

A white solid: **^{1}H NMR (500 MHz, $CDCl_3$)**: δ 7.99 (d, *J* = 8.0 Hz, 2H), 7.38 (d, *J* = 8.0 Hz, 2H), 6.69 (s, 1H), 3.94 (s, 3H), 1.77 (s, 4H), 1.67 (dq, *J* = 14, 7.5 Hz, 4H), 1.58 (dq, *J* = 14, 7.5 Hz, 4H), 0.92 (s, 12H), 0.82 (dd, *J* = 7.5, 7.5 Hz, 12H).

^{13}C NMR (125 MHz, $CDCl_3$): δ 167.4, 148.1, 146.7, 144.3, 135.2, 132.9, 128.6, 127.5, 119.3, 53.6, 52.2, 47.6, 43.7, 32.8, 31.9, 9.3.

HRMS (ESI (+)) *m/z* calcd for $C_{64}H_{88}NaO_4^+$ $[2M+Na]^+$ 943.6575, found 943.6624.

IR (neat) 3032, 2959, 2934, 2874, 1728, 1457, 1434, 1267, 1099, 730 cm^{-1}.

mp 168-170 ºC.

***N,N*-diisopropyl-4-mesitylcubane-1-carboxamide (37):**

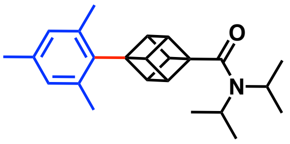

Compound (**37**) was prepared according to **Representative Procedure 4**, using 4-iodo-*N,N*-diisopropylcubane-1-carboxamide (196.8 mg, 0.55 mmol), palladium(II) acetate (11.2 mg, 0.05 mmol) as a catalyst, CuI as a copper source, and **L2** (61.6 mg, 0.15 mmol) as a ligand. The reaction was carried out at room temperature for 24 h. The reaction was carried out at room temperature and the desired product was obtained in 49% ^{1}H NMR yield with this procedure. The obtained material was purified by silica gel flash column chromatography (eluent: CH_2Cl_2/Et_2O/n-hexane) and PTLC (eluent: Et_2O/n-hexane) gave the desired compound **37** in 31% isolated yield. Byprodcuts including an cuneane derivative having mesityl group (the stereochemistry was unidentified) were also obtained.

Colorless oil: **^{1}H NMR (500 MHz, $CDCl_3$)**: δ 6.75 (s, 2H), 4.42-4.40 (m, 3H), 4.24-4.22 (m, 3H), 3.58 (sep, J = 6.9 Hz, 1H), 3.32 (sep, J = 6.9 Hz, 1H), 2.32 (s, 6H), 2.22 (s, 3H), 1.44 (d, J = 6.9 Hz, 6H), 1.22 (d, J = 6.9 Hz, 6H).

^{13}C NMR (125 MHz, $CDCl_3$): δ 171.1, 136.2, 135.5, 135.3, 129.9, 62.4, 57.8, 49.9, 48.3, 46.2, 45.9, 21.6, 21.2, 20.7 (overlap).

HRMS (ESI (+)) *m/z* calcd for $C_{24}H_{32}NO^+$ [$(M+H)^+$] 350.2478, found 350.2483.

IR (neat) 2965, 2926, 1620, 1439, 1342, 1214, 1035, 849, 751, 730 cm^{-1}.

3-3. X Ray crystallographic data

< Top view >

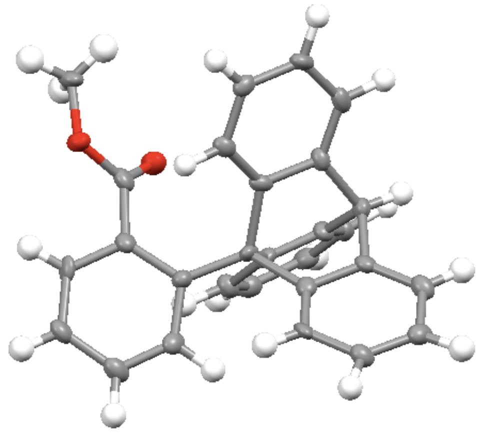

< Side view >

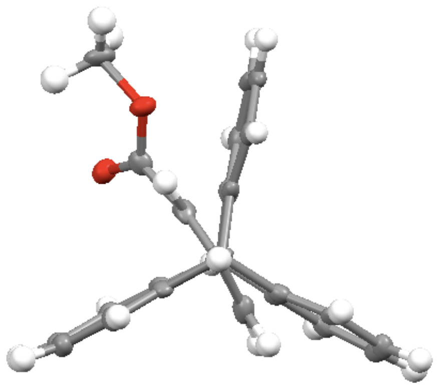

< ORTEP >

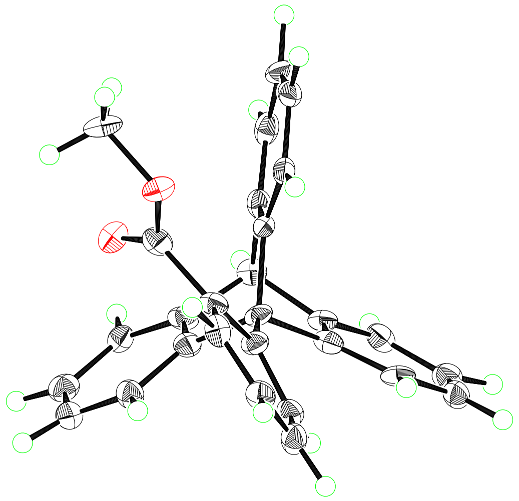

Fig. S15. X-Ray crystallographic structures for compound **9**.

Table S6. Crystal data and structure refinement for compound **9**.

Identification Code	CCDC 1566502
Empirical Formula	C28H20O2
Molecular Formula	C28H20O2
Formula Weight	388.46
Temperature	93 K
Radiation CuKa	l = 1.54187 Å
Crystal System	trigonal
Space Group	P31 (#144)
Lattice Parameters	a = 9.3566(6) Å
	c = 19.436(2) Å γ = 120°
Volume	V = 1473.5(2) Å^3
Z value	3
D_{calc}	1.313 g/cm^3
Absorption coefficient	0.640 mm^{-1}
F(000)	612.00
Crystal Dimensions	0.300 X 0.300 X 0.300 mm
Crystal Color, Habit	colorless, prism
Theta range for data collection	5.459 to 68.11°.
Reflections Collected	15478
Independent Reflections	Unique: 3499 (R_{int} = 0.0720)
Absorption Corrections	Lorentz-polarization
Max. and min. transmission	0.674 and 0.825
Refinement Method	Full-matrix least-squares on F^2
Goodness of Fit Indicator	1.039
Residuals: R1 (I>2.00s(I))	0.0431
Residuals: R (All reflections)	0.0859
Residuals: wR2 (All reflections)	0.0943
Extinction Coefficient	n/a
Largest diff. Peak and Hole	–0.91 and 0.73 e-/Å^3

Table S7. Atomic coordinates and B_{iso}/B_{eq}.

atom	x	y	z	Beq
O(1)	0.1221(3)	-0.1939(2)	0.39600(10)	1.96(5)
O(2)	0.2898(3)	-0.2973(2)	0.37514(10)	1.81(5)
C(3)	0.3334(4)	-0.0678(4)	0.61702(15)	1.77(7)
C(4)	0.1934(4)	0.1613(3)	0.41344(15)	1.48(7)
C(5)	-0.0325(4)	0.1716(4)	0.46841(15)	1.84(7)
C(6)	0.4134(4)	-0.0930(4)	0.56309(14)	1.70(7)
C(7)	0.6338(4)	0.4789(4)	0.43618(14)	1.60(7)
C(8)	0.1083(4)	0.1238(4)	0.35122(15)	1.65(7)
C(9)	0.6315(4)	0.6640(4)	0.52184(15)	1.90(7)
C(10)	0.7107(4)	0.6317(4)	0.46929(14)	1.82(7)
C(11)	0.4737(4)	0.3603(4)	0.45366(14)	1.42(7)
C(12)	0.2365(4)	0.2421(4)	0.53248(15)	1.54(7)
C(13)	0.1228(4)	0.1875(4)	0.47137(15)	1.56(7)
C(14)	0.5228(4)	0.2605(4)	0.31191(14)	1.72(7)
C(15)	0.2708(4)	0.0385(4)	0.60921(15)	1.71(7)
C(16)	0.4539(4)	-0.0516(4)	0.28189(15)	1.54(7)
C(17)	0.4277(3)	-0.0177(3)	0.49940(15)	1.49(7)
C(18)	0.3983(4)	0.3917(4)	0.50948(15)	1.54(7)
C(19)	0.4762(4)	0.5424(4)	0.54318(15)	1.81(7)
C(20)	0.5902(4)	0.2336(4)	0.25434(15)	1.93(7)
C(21)	0.4269(4)	0.1375(4)	0.35911(14)	1.45(7)
C(22)	0.2837(4)	0.1117(4)	0.54607(14)	1.41(7)
C(23)	0.2505(4)	-0.1756(4)	0.37478(14)	1.53(7)
C(24)	0.3562(4)	0.0795(4)	0.48988(15)	1.44(7)
C(25)	0.3832(4)	-0.0250(4)	0.34001(15)	1.44(7)
C(26)	-0.0467(4)	0.1114(4)	0.34809(16)	1.94(7)
C(27)	0.3655(4)	0.1802(4)	0.42465(15)	1.36(7)
C(28)	0.5610(4)	0.0761(4)	0.24003(15)	1.94(7)
C(29)	-0.1174(4)	0.1335(4)	0.40682(16)	1.99(8)
C(30)	0.1668(4)	-0.4518(4)	0.40497(16)	2.32(8)

4. Computational details

General information

All the calculations were performed on the full structures of the reported compounds. DFT calculations were performed with Gaussian 09 program (Revision D.01)[S24] and the GRRM 11 (Version 11.03, based on Gaussian 09) program.[S25] Structure optimizations were carried out at the *ω*B97X-D[S6] level in the gas phase using the SDD basis set[S26] for Cu, Zn, Pd, and Br, and the 6-31+G* basis set[S27] for H, C, O, B, P, and Cl. All stationary points were optimized without any symmetry assumptions and characterized by normal coordinate analysis at the same level of theory (number of imaginary frequencies, NIMAG, 0 for minima and 1 for TSs). The intrinsic reaction coordinate (IRC) method was used to track minimum energy paths from transition structures to the corresponding local minima.[S28] NBO (Natural Bond Orbital) calculations were performed with NBO 6.0 program[S8] at the *ω*B97X-D level using the LanL2DZ[S29] for Cu, Zn, Pd, and Br, and the 6-31G* basis set for other atoms. All structures with their associated free enthalpy and Gibbs free energies as well as their Cartesian coordinates are detailed in the following section.

Cartesian coordinates and energies

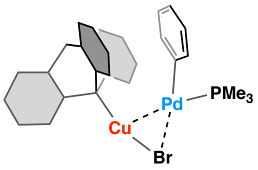

$IM_{Cu}1$

Energy (RwB97XD): -1801.11272194 A.U.

Gibbs Free Energy: -1800.700286 A.U.

Pd	-1.802307	-0.373060	-0.421012
Br	-1.626861	-2.951482	-0.981377
P	-3.535012	-0.075385	0.961826
C	-4.081090	-1.694855	1.613087
H	-3.249709	-2.177829	2.133967
H	-4.921542	-1.564269	2.304023
H	-4.379370	-2.343561	0.785465
C	-5.046544	0.654946	0.241703
H	-5.363135	0.066799	-0.624332
H	-5.852118	0.667784	0.984030
H	-4.837108	1.676969	-0.084692
C	-3.216959	0.921502	2.457643
H	-4.045346	0.812649	3.166161
H	-2.284821	0.592660	2.926511
H	-3.103199	1.972563	2.181665
C	-1.756757	1.605525	-0.400749
C	-2.413761	2.280293	-1.432280
C	-0.889425	2.297525	0.446971
C	-2.161611	3.637086	-1.647346
H	-3.104444	1.753452	-2.087500
C	-0.637381	3.649132	0.220738
H	-0.380397	1.785256	1.257849
C	-1.271238	4.320983	-0.823713
H	-2.659810	4.153749	-2.463685
H	0.070076	4.170761	0.859333
H	-1.066850	5.373681	-0.997294
C	1.770953	-0.559390	-0.111015
C	3.238157	-1.012950	-0.101266
C	1.537383	-0.081566	1.334488
C	1.792542	0.726227	-0.955370
C	3.724770	-2.255953	-0.483573
C	4.141388	-0.033843	0.347844
C	2.421624	0.919707	1.770014
C	0.575425	-0.551665	2.223434
C	2.682392	1.711885	-0.501315
C	1.040667	0.984391	-2.094886
C	5.096822	-2.523191	-0.419498
H	3.036103	-3.021672	-0.835265
C	5.501637	-0.291822	0.414274
C	3.464827	1.277523	0.725943
C	2.308357	1.482904	3.032532
C	0.464300	-0.000337	3.505097
H	-0.092110	-1.356669	1.922028
C	2.793397	2.935962	-1.142912
C	1.142779	2.215420	-2.749037
H	0.364459	0.223060	-2.481898
C	5.982452	-1.547374	0.027197
H	5.470370	-3.498062	-0.721301
H	6.189199	0.475881	0.763242
H	4.171739	2.036752	1.072962
C	1.315478	1.025407	3.905322
H	2.995545	2.266100	3.345699
H	-0.282176	-0.385619	4.196168
C	2.011760	3.191544	-2.273413
H	3.485459	3.688665	-0.770539
H	0.534189	2.409537	-3.627864
H	7.047329	-1.758196	0.075246
H	1.224702	1.455802	4.898944
H	2.085900	4.150046	-2.780022
Cu	0.435325	-1.853205	-0.651415

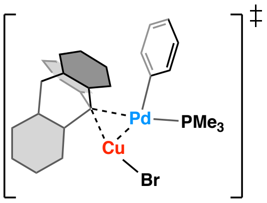

TS$_{Cu}$1

Energy (RwB97XD): -1801.07933653 A.U.

Gibbs Free Energy: -1800.666174 A.U.

Pd	1.230065	-0.241222	-0.164582
Br	1.578832	-3.026813	0.622926
P	3.451259	-0.162447	-0.738216
C	3.865568	-1.456983	-1.967297
H	3.284325	-1.295179	-2.880225
H	4.933255	-1.429022	-2.212196
H	3.606633	-2.439439	-1.564297
C	4.605370	-0.478857	0.644518
H	4.333645	-1.420168	1.130468
H	5.638531	-0.533945	0.284103
H	4.525470	0.331202	1.375204
C	4.115166	1.360809	-1.505930
H	5.154075	1.206609	-1.817811
H	3.509874	1.628841	-2.376850
H	4.064155	2.189236	-0.794493
C	1.466733	1.716738	0.202154
C	2.057097	2.038961	1.432224
C	1.105696	2.756723	-0.654167
C	2.253143	3.367147	1.808056
H	2.357518	1.251786	2.120612
C	1.301626	4.087308	-0.276472
H	0.656747	2.550113	-1.618158
C	1.874706	4.400655	0.952899
H	2.700388	3.590705	2.773614
H	0.998617	4.880706	-0.955221
H	2.023846	5.436908	1.242960
C	-1.182904	-0.065482	-0.065878
C	-2.344215	-1.118926	-0.207903
C	-1.468212	0.727924	-1.355562
C	-1.602919	0.799032	1.135126
C	-2.236295	-2.486840	-0.471054
C	-3.634185	-0.541900	-0.178670
C	-2.737949	1.330100	-1.387377
C	-0.685282	0.774800	-2.503403
C	-2.870090	1.388587	1.057646
C	-0.872246	0.965898	2.307738
C	-3.389279	-3.287295	-0.541660
H	-1.274341	-2.949386	-0.698963
C	-4.767518	-1.326454	-0.268466
C	-3.610987	0.982855	-0.190641
C	-3.161198	2.056764	-2.490624
C	-1.113109	1.486681	-3.627254
H	0.275867	0.264105	-2.517703
C	-3.374117	2.175741	2.083028
C	-1.368394	1.750828	3.350719
H	0.098361	0.490626	2.412513
C	-4.644978	-2.716337	-0.416132
H	-3.285719	-4.353395	-0.720847
H	-5.751862	-0.863966	-0.253305
H	-4.613096	1.415093	-0.259099
C	-2.336195	2.150106	-3.614613
H	-4.143667	2.523746	-2.490890
H	-0.483925	1.521830	-4.512815
C	-2.609421	2.369595	3.235176
H	-4.362267	2.621790	1.994332
H	-0.776241	1.880468	4.252403
H	-5.535643	-3.335008	-0.477379
H	-2.663686	2.712108	-4.484796
H	-2.990643	2.986715	4.043999
Cu	-0.403617	-1.815199	0.928598

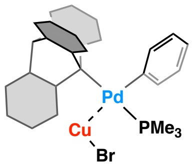

IM*Cu*2

Energy (RwB97XD): -1801.10259215 A.U.

Gibbs Free Energy: -1800.691059 A.U.

Pd	1.047540	0.394952	-0.269040
Br	1.469000	-3.325351	0.529676
P	3.401255	0.118341	-0.239503
C	4.126711	-1.160405	-1.337642
H	3.944869	-0.892633	-2.383428
H	5.206829	-1.250322	-1.175475
H	3.649039	-2.123369	-1.135384
C	3.969959	-0.381775	1.430324
H	3.477638	-1.316550	1.713639
H	5.056906	-0.518922	1.456121
H	3.689064	0.393770	2.150223
C	4.449866	1.582644	-0.585236
H	5.509173	1.334214	-0.455991
H	4.284892	1.931495	-1.608817
H	4.179692	2.398487	0.091218
C	1.225133	2.351909	-0.031216
C	1.018756	2.946140	1.216632
C	1.605361	3.158664	-1.110296
C	1.196951	4.321121	1.382398
H	0.686154	2.351091	2.062258
C	1.772416	4.533840	-0.943836
H	1.759426	2.720870	-2.093333
C	1.571620	5.121272	0.304795
H	1.025504	4.767454	2.358800
H	2.057383	5.146569	-1.795854
H	1.700207	6.192182	0.434732
C	-1.059785	0.415256	-0.045092
C	-1.573248	-0.668489	-1.017666
C	-2.110278	1.526875	-0.241171
C	-1.343868	-0.145309	1.367689
C	-0.930205	-1.103485	-2.198923
C	-2.897940	-1.121752	-0.768404
C	-3.430784	1.096851	-0.003362
C	-1.903857	2.838897	-0.649190
C	-2.660518	-0.571602	1.587711
C	-0.423586	-0.276420	2.404818
C	-1.566454	-2.047174	-3.045017
H	-0.016636	-0.609503	-2.531782
C	-3.510993	-2.028885	-1.607106
C	-3.548862	-0.375334	0.379034
C	-4.508933	1.957046	-0.141415
C	-2.988606	3.711261	-0.788019
H	-0.904064	3.193560	-0.859335
C	-3.059165	-1.110807	2.801900
C	-0.814600	-0.815567	3.632505
H	0.611112	0.022625	2.256493
C	-2.830626	-2.515047	-2.739255
H	-1.059108	-2.377734	-3.945958
H	-4.531350	-2.348147	-1.409591
H	-4.583040	-0.686481	0.549477
C	-4.285216	3.280443	-0.530805
H	-5.519495	1.601942	0.048945
H	-2.808091	4.736331	-1.100258
C	-2.127046	-1.230938	3.834713
H	-4.087231	-1.435848	2.945579
H	-0.082823	-0.919130	4.428616
H	-3.315643	-3.235542	-3.390885
H	-5.122462	3.964531	-0.638242
H	-2.425637	-1.653574	4.789858
Cu	-0.109523	-2.230288	-0.644314

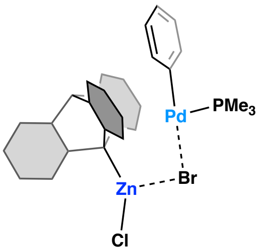

IM$_{Zn}$1

Energy (RwB97XD): -2291.161275348311 A.U.

Gibbs Free Energy: -2290.748233214304 A.U.

Pd	-1.366043	0.419749	0.895384
Br	-2.206935	-2.047839	0.451821
P	-3.226007	1.306327	-0.058367
C	-4.773766	0.478966	0.458391
H	-4.917056	0.607348	1.535080
H	-5.626101	0.915624	-0.073771
H	-4.714693	-0.590161	0.243042
C	-3.163773	1.082919	-1.870024
H	-3.050542	0.018615	-2.094498
H	-4.077704	1.464392	-2.338600
H	-2.295718	1.614104	-2.271320
C	-3.633533	3.077085	0.150376
H	-4.574738	3.297532	-0.365318
H	-3.737463	3.312269	1.213033
H	-2.835077	3.702152	-0.256024
C	-0.781890	2.314721	1.047794
C	-0.942603	3.045508	2.227398
C	-0.134541	2.908421	-0.037658
C	-0.424553	4.337228	2.332148
H	-1.459068	2.607273	3.077836
C	0.390874	4.196632	0.070115
H	-0.022878	2.366562	-0.972246
C	0.248511	4.915161	1.256196
H	-0.546994	4.891900	3.259309
H	0.907250	4.636367	-0.779923
H	0.652134	5.920456	1.340092
Zn	0.105857	-2.853445	-0.286983
Cl	-0.045074	-4.947076	-0.799154
C	1.375753	-1.317748	-0.237445
C	1.295897	-0.422873	1.009059
C	1.183597	-0.344788	-1.411606
C	2.859833	-1.722929	-0.306120
C	0.521274	-0.641518	2.157754
C	2.202291	0.658395	0.998760
C	2.093022	0.724278	-1.433967
C	0.220716	-0.431543	-2.409816
C	3.753953	-0.641194	-0.317431
C	3.360198	-3.017913	-0.357737
C	0.659585	0.211897	3.272656
H	-0.065770	-1.549890	2.262507
C	2.327377	1.493607	2.092005
C	3.053173	0.709019	-0.259479
C	2.066981	1.667585	-2.449511
C	0.179546	0.524061	-3.430933
H	-0.503628	-1.244361	-2.409211
C	5.123839	-0.842052	-0.375853
C	4.742357	-3.227189	-0.417306
H	2.687222	-3.872308	-0.361517
C	1.547361	1.272676	3.235774
H	0.070884	0.016294	4.164154
H	3.034514	2.319086	2.067886
H	3.748224	1.552829	-0.264788
C	1.102005	1.566179	-3.456616
H	2.784857	2.484720	-2.455241
H	-0.568823	0.440880	-4.214578
C	5.620608	-2.148334	-0.425676
H	5.803694	0.007070	-0.383612
H	5.127641	-4.241975	-0.459281
H	1.644155	1.934877	4.090632
H	1.074571	2.299130	-4.258158
H	6.692612	-2.318509	-0.472203

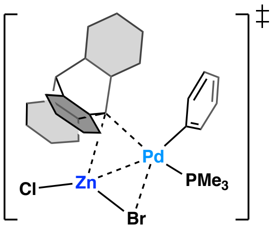

$TS_{Zn}1$

Energy (RwB97XD): -2291.11512251 A.U.

Gibbs Free Energy: -2290.703614 A.U.

Pd	-0.976591	0.134101	-0.443630
Br	-2.147193	-2.697733	-0.401194
P	-3.199339	0.690281	-1.167744
C	-4.617901	0.224560	-0.111232
H	-4.564814	0.775307	0.831976
H	-5.562000	0.466998	-0.611066
H	-4.581299	-0.847231	0.101269
C	-3.592409	-0.061106	-2.793773
H	-3.566074	-1.150842	-2.708717
H	-4.583108	0.252696	-3.140511
H	-2.844038	0.244416	-3.532057
C	-3.467835	2.479632	-1.456681
H	-4.458196	2.661505	-1.887900
H	-3.376931	3.018764	-0.509361
H	-2.703198	2.866733	-2.137581
C	-1.193514	1.689657	0.792877
C	-2.028471	1.513938	1.902041
C	-0.582938	2.923898	0.582610
C	-2.239534	2.563336	2.796611
H	-2.501822	0.554110	2.095658
C	-0.806912	3.973023	1.476064
H	0.088184	3.078116	-0.254839
C	-1.632558	3.799680	2.583969
H	-2.876319	2.406180	3.663376
H	-0.316001	4.927514	1.305418
H	-1.795074	4.616980	3.280569
Zn	-0.458159	-1.815873	1.065983
Cl	0.221245	-2.458362	2.976554
C	1.181972	0.133185	-0.292857
C	1.901106	0.591985	0.998933
C	1.607112	1.146292	-1.393600
C	1.954391	-1.138116	-0.733528
C	1.351035	0.774594	2.262034
C	3.283051	0.787528	0.852771
C	2.996333	1.327956	-1.496081
C	0.790709	1.817616	-2.300763
C	3.348970	-0.956849	-0.814709
C	1.432998	-2.375062	-1.090425
C	2.146489	1.172466	3.338239
H	0.296741	0.611971	2.436677
C	4.083281	1.187425	1.912365
C	3.790501	0.467794	-0.534050
C	3.547261	2.185231	-2.436598
C	1.337810	2.679842	-3.255573
H	-0.289434	1.691869	-2.271353
C	4.190457	-1.999813	-1.158308
C	2.279478	-3.438679	-1.432962
H	0.362785	-2.532598	-1.171458
C	3.508286	1.390835	3.167724
H	1.688523	1.302910	4.314343
H	5.151745	1.326720	1.763851
H	4.870524	0.603701	-0.636024
C	2.712784	2.873928	-3.319850
H	4.626433	2.308800	-2.490679
H	0.680609	3.198650	-3.948274
C	3.654106	-3.258969	-1.452779
H	5.264808	-1.836987	-1.204420
H	1.846104	-4.400238	-1.692017
H	4.124533	1.701712	4.006664
H	3.137709	3.546101	-4.059800
H	4.311909	-4.082607	-1.714523

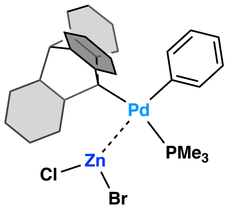

IM$_{Zn}$2

Energy (RwB97XD): -2291.119229745535 A.U.

Gibbs Free Energy: -2290.707536995063 A.U.

Pd	-0.764051	0.507567	-0.366556
Br	-0.757585	-2.976298	1.076656
P	-3.040888	-0.226802	-0.919354
C	-4.208930	-0.833389	0.359408
H	-4.339246	-0.099085	1.159357
H	-5.183208	-1.059122	-0.087874
H	-3.805284	-1.747509	0.807188
C	-2.960186	-1.656789	-2.068713
H	-2.467704	-2.496561	-1.567589
H	-3.966135	-1.964098	-2.374629
H	-2.384476	-1.392663	-2.961722
C	-4.019799	0.996244	-1.876231
H	-4.974737	0.566390	-2.198049
H	-4.211169	1.887526	-1.272655
H	-3.449981	1.303009	-2.758674
C	-1.103299	2.177338	0.711992
C	-2.431323	2.467753	1.044999
C	-0.117716	3.087147	1.111460
C	-2.771239	3.634071	1.728952
H	-3.229790	1.774373	0.814065
C	-0.456276	4.254793	1.793708
H	0.927971	2.910365	0.903294
C	-1.783305	4.538208	2.103186
H	-3.811871	3.818439	1.981784
H	0.334072	4.940722	2.086450
H	-2.042356	5.445783	2.640718
Zn	-1.042730	-0.810591	1.844972
Cl	-2.026589	0.017084	3.569760
C	1.291858	0.668677	-0.339989
C	2.081841	0.545632	0.966843
C	1.945692	1.808636	-1.138227
C	1.674603	-0.578264	-1.163537
C	1.591410	0.591058	2.266946
C	3.459350	0.358472	0.772468
C	3.332318	1.644996	-1.297471
C	1.317403	2.893247	-1.737649
C	3.053779	-0.749122	-1.345624
C	0.797118	-1.473190	-1.759207
C	2.447179	0.403510	3.356202
H	0.553888	0.854163	2.459879
C	4.316357	0.180237	1.848116
C	3.866235	0.355487	-0.689309
C	4.081996	2.569778	-2.008087
C	2.069764	3.825617	-2.458361
H	0.244992	3.028563	-1.632700
C	3.547095	-1.810233	-2.086374
C	1.284555	-2.545234	-2.512454
H	-0.285049	-1.393574	-1.640180
C	3.805554	0.192294	3.149122
H	2.041151	0.436155	4.362878
H	5.379387	0.026697	1.677096
H	4.943337	0.234066	-0.830849
C	3.446465	3.672292	-2.586562
H	5.154442	2.430260	-2.123254
H	1.572117	4.675820	-2.916468
C	2.654748	-2.715734	-2.670903
H	4.619447	-1.934226	-2.217402
H	0.589266	-3.250029	-2.958850
H	4.471006	0.045850	3.994915
H	4.027224	4.401710	-3.144072
H	3.034234	-3.552136	-3.250573

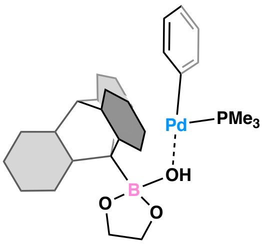

IM$_B$1

Energy (RwB97XD): -1920.09564538 A.U.

Gibbs Free Energy: -1919.593814 A.U.

Pd	-1.206015	0.610598	-0.603857
P	-2.668453	1.874542	0.566337
C	-1.966166	3.516006	0.955032
H	-1.743887	4.054116	0.029933
H	-2.667842	4.100148	1.560613
H	-1.025988	3.383623	1.495517
C	-3.158358	1.206378	2.193064
H	-2.260093	0.941274	2.758965
H	-3.737494	1.951473	2.749709
H	-3.759000	0.304315	2.051734
C	-4.261802	2.250566	-0.249310
H	-4.870114	2.908508	0.381062
H	-4.080885	2.740127	-1.210800
H	-4.803596	1.318202	-0.430397
C	-2.586020	-0.813691	-0.454347
C	-3.542874	-1.003997	-1.457009
C	-2.542077	-1.719419	0.611019
C	-4.415724	-2.092443	-1.413190
H	-3.599895	-0.311385	-2.294280
C	-3.415435	-2.806804	0.657082
H	-1.814748	-1.584759	1.408919
C	-4.353404	-2.999310	-0.356052
H	-5.143440	-2.232032	-2.209064
H	-3.359888	-3.505117	1.488936
H	-5.032677	-3.846700	-0.320719
O	0.337531	2.129017	-0.721954
H	0.256088	2.654396	-1.527342
C	3.181783	3.498932	-0.811112
C	3.309127	2.734410	-2.138080
H	4.141536	3.869112	-0.432691
H	2.500685	4.360967	-0.920273
H	4.295043	2.253078	-2.231454
H	3.166242	3.378320	-3.014946
B	1.824724	1.645237	-0.692109
O	2.647784	2.552761	0.082206
O	2.293264	1.758427	-2.079918
C	1.754455	0.133384	-0.060278
C	1.171876	0.090766	1.369959
C	3.064206	-0.662161	0.068636
C	0.846060	-0.806611	-0.885575
C	0.862903	1.191829	2.163517
C	0.993175	-1.198191	1.891138
C	2.905366	-1.950561	0.603154
C	4.336333	-0.221258	-0.268669
C	0.695996	-2.104960	-0.348679
C	0.293687	-0.534510	-2.150069
C	0.348408	0.999258	3.448894
H	1.052859	2.191063	1.785535
C	0.472662	-1.394562	3.162686
C	1.458990	-2.290483	0.946764
C	3.991869	-2.792240	0.784940
C	5.436128	-1.065149	-0.087000
H	4.476345	0.779859	-0.656596
C	-0.005002	-3.082162	-1.023845
C	-0.418502	-1.543685	-2.833704
H	0.552805	0.384852	-2.667017
C	0.140981	-0.285628	3.945937
H	0.120595	1.862472	4.070061
H	0.340403	-2.402546	3.550415
H	1.346000	-3.294699	1.364846
C	5.268813	-2.344871	0.433836
H	3.851169	-3.788140	1.199621
H	6.429837	-0.714592	-0.352973

C	-0.578542	-2.796189	-2.272833
H	-0.107902	-4.074945	-0.592413
H	-0.833999	-1.324813	-3.813030
H	-0.257417	-0.427693	4.947102
H	6.128384	-2.994981	0.572496
H	-1.141369	-3.562825	-2.796669

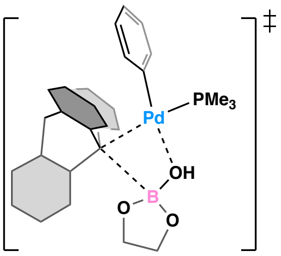

$TS_{B}1$

Energy (RwB97XD): -1920.01879201 A.U.

Gibbs Free Energy: -1919.519812 A.U.

Pd	-1.144326	0.414413	-0.720490
P	-3.025191	1.448799	-0.042502
C	-3.212832	3.097777	-0.812533
H	-3.276160	2.997650	-1.899419
H	-4.116106	3.591386	-0.436781
H	-2.335701	3.703262	-0.571302
C	-3.003893	1.812686	1.747734
H	-2.136576	2.442100	1.964101
H	-3.920940	2.331654	2.048408
H	-2.909258	0.880740	2.311668
C	-4.653079	0.657071	-0.306593
H	-5.455649	1.334162	0.006291
H	-4.779284	0.409940	-1.364614
H	-4.710632	-0.270767	0.268083
C	-2.045813	-1.215940	-0.038716
C	-2.731546	-2.024033	-0.950796
C	-1.929564	-1.628858	1.288830
C	-3.274726	-3.241416	-0.542214
H	-2.835319	-1.715742	-1.987324
C	-2.477008	-2.848016	1.693390
H	-1.393290	-1.023930	2.012222
C	-3.151176	-3.657659	0.782558
H	-3.793329	-3.865730	-1.265675
H	-2.361740	-3.162220	2.727647
H	-3.573713	-4.607024	1.099987
O	-0.240022	2.349667	-1.799239
H	0.320386	2.017185	-2.513026
C	1.002575	4.116898	1.030173
C	2.298107	3.811027	0.257428
H	1.059446	3.815135	2.079957
H	0.717999	5.173540	0.970972
H	2.869898	3.004897	0.730901
H	2.937048	4.686296	0.120283
B	0.571203	2.885930	-0.809181
O	0.003235	3.338631	0.365815
O	1.841533	3.357880	-1.018175
C	1.402612	0.153675	-0.217513
C	1.123805	-0.153673	1.260738
C	2.931023	-0.016441	-0.311628
C	0.970647	-1.081301	-1.021660
C	0.504626	0.712571	2.160512
C	1.561950	-1.395425	1.747755
C	3.402959	-1.254021	0.182432
C	3.847978	0.866809	-0.869160
C	1.346760	-2.336147	-0.486898
C	0.368035	-1.072176	-2.284369
C	0.301184	0.339457	3.493355
H	0.153056	1.673463	1.803626
C	1.347803	-1.789820	3.061035
C	2.296036	-2.183323	0.688443
C	4.750975	-1.573320	0.159282
C	5.211804	0.547113	-0.898006
H	3.492970	1.801439	-1.291729
C	0.974313	-3.520075	-1.091088
C	0.010375	-2.273027	-2.919881
H	0.223996	-0.135787	-2.821367
C	0.708824	-0.914250	3.943774
H	-0.188075	1.028745	4.178291

H 1.685178 -2.766466 3.402589
H 2.681009 -3.141764 1.049421
C 5.665954 -0.660588 -0.379156
H 5.095629 -2.530520 0.546566
H 5.918924 1.248569 -1.334579
C 0.271519 -3.487073 -2.306956
H 1.259195 -4.474448 -0.652852
H -0.471408 -2.243524 -3.893688
H 0.541901 -1.207371 4.977032
H 6.725135 -0.903352 -0.402306
H -0.023098 -4.416577 -2.786254

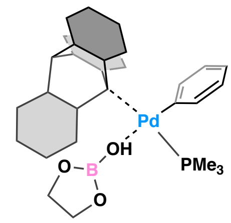

IM$_{B}$2

Energy (RwB97XD): -1920.09006262 A.U.

Gibbs Free Energy: -1919.593378 A.U.

Pd -1.117471 0.039223 0.468579
P -3.325882 -0.786652 0.539287
C -3.599603 -2.357310 1.449636
H -3.313260 -2.233940 2.498400
H -4.649800 -2.666285 1.396068
H -2.972247 -3.137536 1.010292
C -3.911118 -1.195967 -1.149397
H -3.246613 -1.953524 -1.576475
H -4.938925 -1.576406 -1.141233
H -3.862858 -0.295605 -1.770161
C -4.665658 0.290342 1.176894
H -5.635632 -0.215369 1.112240
H -4.466713 0.554225 2.220287
H -4.697171 1.215699 0.595169
C -1.852548 1.754818 -0.180756
C -2.365058 2.706094 0.713397
C -1.930129 2.032867 -1.551697
C -2.921063 3.900460 0.254001
H -2.313706 2.525824 1.784666
C -2.492222 3.224259 -2.013521
H -1.515129 1.333638 -2.273720
C -2.988447 4.165960 -1.113621
H -3.297458 4.628282 0.969317
H -2.526769 3.422006 -3.082337
H -3.416823 5.097767 -1.472690
O -0.305372 -2.061077 1.147872
H 0.483181 -1.850221 1.679311
C -0.448198 -4.456174 -1.486041
C 1.019901 -4.588971 -1.001434
H -0.509967 -4.142474 -2.531642
H -1.021599 -5.378786 -1.353681
H 1.734448 -4.250054 -1.755817
H 1.270394 -5.607031 -0.691191
B -0.058515 -3.038680 0.219129
O -1.025565 -3.438268 -0.665959
O 1.116291 -3.724692 0.130494
C 0.926904 0.592142 0.164446
C 1.395106 -0.289986 -1.015438
C 1.731258 0.138317 1.400677
C 1.597971 1.954532 -0.142370
C 0.575730 -0.914882 -1.954132
C 2.784606 -0.394725 -1.177689
C 3.115561 0.006110 1.202465
C 1.225136 -0.063637 2.684876
C 2.992487 1.859167 -0.335764
C 1.011746 3.212589 -0.204340
C 1.130528 -1.651239 -3.006044
H -0.505113 -0.823794 -1.876486
C 3.345106 -1.139295 -2.205202
C 3.548783 0.447123 -0.180875
C 3.957412 -0.395533 2.227796
C 2.069364 -0.467674 3.727239
H 0.168333 0.113676 2.880298

C	3.766176	2.975000	-0.611122
C	1.789237	4.343044	-0.481677
H	-0.049094	3.329845	-0.036413
C	2.510969	-1.777556	-3.126856
H	0.474903	-2.113921	-3.740095
H	4.426407	-1.203629	-2.305344
H	4.632322	0.401641	-0.322909
C	3.428404	-0.651403	3.496877
H	5.026227	-0.491852	2.049583
H	1.658349	-0.625706	4.720712
C	3.158258	4.231348	-0.691687
H	4.839729	2.871983	-0.755889
H	1.308316	5.316459	-0.531267
H	2.940379	-2.348358	-3.946185
H	4.082323	-0.967138	4.304920
H	3.755623	5.112890	-0.908387

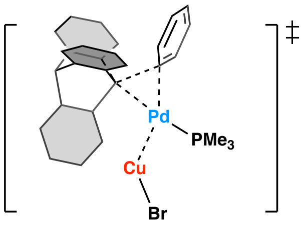

TS$_{Cu}$2

Energy (RwB97XD): -1801.07111253 A.U.

Gibbs Free Energy: -1800.660717 A.U.

Pd	-0.732579	0.955382	0.206132
Br	-3.260682	-2.139964	0.133214
P	-2.829541	1.962342	0.137903
C	-3.995191	1.622431	1.514394
H	-3.539209	1.918075	2.464409
H	-4.938876	2.164920	1.383595
H	-4.194781	0.546785	1.547622
C	-3.866851	1.611108	-1.336365
H	-4.091150	0.539995	-1.363873
H	-4.805538	2.176886	-1.308362
H	-3.316475	1.876065	-2.244818
C	-2.726632	3.796941	0.127887
H	-3.718181	4.260304	0.063278
H	-2.226578	4.136886	1.040042
H	-2.117222	4.118391	-0.722601
C	0.893005	2.095389	-0.120771
C	1.083478	2.622664	-1.408275
C	1.336890	2.850326	0.977951
C	1.639146	3.888602	-1.584519
H	0.809915	2.048293	-2.286713
C	1.888994	4.116628	0.794942
H	1.271281	2.447135	1.983067
C	2.039602	4.647777	-0.485538
H	1.766140	4.278066	-2.591546
H	2.215536	4.684400	1.662539
H	2.476452	5.632121	-0.625969
C	1.304377	0.023725	0.090803
C	1.102233	-0.689349	1.451895
C	2.858604	0.069065	-0.069148
C	0.887473	-0.980069	-1.018218
C	0.447076	-0.194144	2.578845
C	1.666354	-1.970103	1.516176
C	3.431452	-1.214793	0.041999
C	3.711902	1.144913	-0.294049
C	1.491638	-2.254282	-0.902251
C	0.116109	-0.716639	-2.173690
C	0.323210	-0.985112	3.723662
H	0.013431	0.802064	2.579690
C	1.544281	-2.761261	2.648092
C	2.437087	-2.343530	0.271147
C	4.798495	-1.415870	-0.063782
C	5.092361	0.943969	-0.398887
H	3.337969	2.152675	-0.392161
C	1.306036	-3.236479	-1.860081
C	-0.089144	-1.736577	-3.132485
H	-0.255513	0.283662	-2.374390
C	0.859384	-2.267308	3.759216
H	-0.202845	-0.590752	4.588065
H	1.985810	-3.754740	2.668516

H 2.921521 -3.320218 0.346786
C 5.641750 -0.325815 -0.284252
H 5.206234 -2.419981 0.027310
H 5.735244 1.802248 -0.572960
C 0.497399 -2.984745 -2.974420
H 1.796574 -4.199651 -1.746896
H -0.694046 -1.520508 -4.007394
H 0.752669 -2.880411 4.649236
H 6.714933 -0.472400 -0.366160
H 0.335588 -3.759572 -3.716969
Cu -1.355048 -1.433774 -0.870280

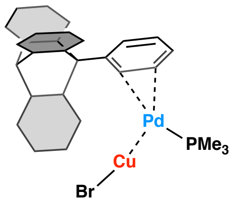

PD_{Cu}

Energy (RwB97XD): -1801.15593871 A.U.

Gibbs Free Energy: -1800.742446 A.U.

Pd -1.409048 1.558684 -0.354143
Br -1.807329 -3.161295 0.043736
P -3.503663 0.807826 0.145525
C -3.836187 0.017540 1.770770
H -3.360900 0.603646 2.563450
H -4.915088 -0.034635 1.958007
H -3.417492 -0.993627 1.783263
C -4.489634 -0.216898 -1.017204
H -4.095466 -1.237450 -1.040689
H -5.540015 -0.241814 -0.704331
H -4.422741 0.206988 -2.023622
C -4.558823 2.320782 0.265021
H -5.589165 2.053428 0.528834
H -4.157482 2.994942 1.027488
H -4.561189 2.849551 -0.692745
C 0.957691 1.813392 0.016776
C 0.548073 2.497919 -1.150785
C 0.989293 2.567678 1.219486
C 0.102452 3.837861 -1.087300
H 0.716496 2.064524 -2.128580
C 0.576038 3.887243 1.268731
H 1.394935 2.108349 2.114384
C 0.101926 4.526900 0.114945
H -0.204543 4.330075 -2.005061
H 0.629664 4.428852 2.208445
H -0.233345 5.558760 0.155701
C 1.674273 0.466375 0.000697
C 1.368895 -0.424126 1.239689
C 3.202693 0.715895 0.008722
C 1.435149 -0.522474 -1.180937
C 0.351552 -0.259899 2.176568
C 2.172249 -1.569260 1.308706
C 3.970122 -0.449597 0.098507
C 3.837556 1.948760 -0.086953
C 2.240499 -1.671829 -1.084232
C 0.501414 -0.464322 -2.215577
C 0.168810 -1.211430 3.184329
H -0.320076 0.590108 2.131636
C 1.993431 -2.513458 2.306436
C 3.134016 -1.706989 0.143804
C 5.355482 -0.392139 0.112902
C 5.234260 2.010607 -0.075902
H 3.264701 2.867299 -0.170599
C 2.138189 -2.707137 -1.997023
C 0.398349 -1.511073 -3.140685
H -0.178495 0.370587 -2.331615
C 0.987749 -2.330112 3.257023
H -0.637581 -1.078986 3.899696
H 2.615786 -3.404409 2.328344
H 3.734722 -2.618291 0.198070
C 5.991817 0.848477 0.028476
H 5.937767 -1.307622 0.184468
H 5.726789 2.976015 -0.149921
C 1.213736 -2.626565 -3.040264

H	2.760356	-3.590947	-1.881545
H	-0.347493	-1.446260	-3.927101
H	0.829767	-3.074499	4.031484
H	7.076637	0.903658	0.038790
H	1.117832	-3.443530	-3.748717
Cu	-1.371141	-0.927551	-0.222032

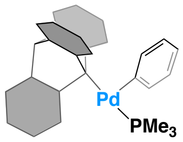

IM2''

Energy (RwB97XD): -1590.26936445 A.U.

GIbbs Free Energy: -1589.854569 A.U.

Pd	-1.071436	-0.576847	0.211240
P	-3.262492	-1.502114	0.003045
C	-3.297736	-3.286713	-0.441240
H	-2.866406	-3.881324	0.370447
H	-4.320382	-3.632517	-0.629598
H	-2.694905	-3.453070	-1.339474
C	-4.204573	-0.720891	-1.361896
H	-3.643917	-0.822983	-2.296052
H	-5.194411	-1.175568	-1.480521
H	-4.312700	0.347699	-1.152041
C	-4.455446	-1.422029	1.397817
H	-5.403934	-1.903315	1.133749
H	-4.035753	-1.916606	2.279102
H	-4.650245	-0.375466	1.649507
C	-1.769675	1.276686	0.027823
C	-1.491128	2.122578	-1.048496
C	-2.624388	1.742104	1.038127
C	-2.074678	3.389021	-1.126163
H	-0.794954	1.820995	-1.823011
C	-3.196954	3.012603	0.964610
H	-2.828999	1.124491	1.909400
C	-2.931060	3.840464	-0.124744
H	-1.840983	4.030100	-1.972613
H	-3.846598	3.355380	1.766638
H	-3.375949	4.829767	-0.185454
C	0.921010	-0.032067	0.082682
C	1.621567	-1.241795	0.727115
C	1.598840	1.177825	0.737363
C	1.427575	-0.061599	-1.372102
C	1.001440	-2.282729	1.409989
C	3.018433	-1.250818	0.583707
C	2.998548	1.178499	0.601525
C	0.996139	2.204176	1.454013
C	2.825725	-0.036674	-1.496411
C	0.657939	-0.171933	-2.525650
C	1.762667	-3.342022	1.919536
H	-0.077161	-2.276168	1.596139
C	3.778333	-2.287797	1.098589
C	3.536730	-0.025047	-0.155338
C	3.775478	2.190842	1.144059
C	1.775041	3.227213	2.004076
H	-0.078812	2.216299	1.585383
C	3.440735	-0.073075	-2.738714
C	1.269917	-0.208261	-3.782712
H	-0.424830	-0.239481	-2.448822
C	3.144048	-3.344700	1.763231
H	1.272995	-4.153431	2.450941
H	4.860683	-2.279951	0.990504
H	4.626196	-0.015598	-0.250007
C	3.157341	3.228014	1.847788
H	4.857000	2.172165	1.026993
H	1.290274	4.027440	2.557032
C	2.655625	-0.149034	-3.892653
H	4.525922	-0.052237	-2.811929
H	0.656675	-0.286350	-4.676670
H	3.734908	-4.162164	2.166826
H	3.756646	4.026990	2.275927
H	3.128123	-0.175437	-4.870720

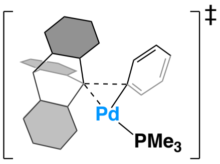

TS2''

Energy (RwB97XD): -1590.23893634 A.U.

GIbbs Free Energy: -1589.822505 A.U.

Pd	1.293620	-0.561062	-0.075001
P	3.591854	-0.794143	-0.004363
C	4.511285	-2.084537	-0.943719
H	4.263595	-2.008408	-2.007093
H	5.595664	-1.977567	-0.821240
H	4.210274	-3.078429	-0.597720
C	4.316900	-0.976891	1.678098
H	3.998805	-1.927104	2.118391
H	5.412586	-0.941425	1.653737
H	3.948088	-0.166346	2.314733
C	4.397313	0.751908	-0.585810
H	5.485667	0.718724	-0.457376
H	4.160620	0.910349	-1.642402
H	3.985209	1.598770	-0.027164
C	0.849320	1.399515	-0.022265
C	1.062464	2.080345	1.188318
C	1.000710	2.118139	-1.220094
C	1.474256	3.412130	1.195265
H	0.892532	1.578710	2.135505
C	1.412716	3.449375	-1.206999
H	0.783203	1.645728	-2.172554
C	1.660277	4.104946	-0.001026
H	1.639547	3.912914	2.146156
H	1.530121	3.979270	-2.149129
H	1.976665	5.144010	0.007062
C	-0.841519	0.076930	-0.005882
C	-1.192365	-0.765985	-1.256762
C	-1.978988	1.143597	0.033802
C	-1.135477	-0.796203	1.239471
C	-0.415784	-0.953153	-2.399957
C	-2.460748	-1.363030	-1.204076
C	-3.253102	0.537674	0.054358
C	-1.919583	2.533554	0.053693
C	-2.405914	-1.390975	1.229996
C	-0.308895	-1.012257	2.341775
C	-0.882236	-1.758096	-3.444307
H	0.560070	-0.483653	-2.490120
C	-2.927825	-2.162434	-2.235916
C	-3.239102	-0.982933	0.035474
C	-4.416855	1.289022	0.091614
C	-3.094138	3.293283	0.091237
H	-0.977291	3.060039	0.040981
C	-2.828163	-2.214453	2.262503
C	-0.730295	-1.839999	3.387173
H	0.671811	-0.548850	2.398533
C	-2.128444	-2.369080	-3.362734
H	-0.262445	-1.900295	-4.325229
H	-3.915574	-2.613023	-2.170379
H	-4.244952	-1.411012	0.053326
C	-4.340747	2.682868	0.109992
H	-5.382647	0.788815	0.106769
H	-3.017534	4.377089	0.105846
C	-1.980273	-2.447815	3.347540
H	-3.818435	-2.663055	2.229889
H	-0.072006	-2.003259	4.235983
H	-2.486271	-2.993360	-4.176640
H	-5.247755	3.280205	0.139091
H	-2.302614	-3.090507	4.161964

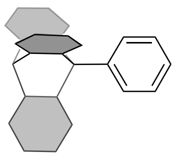

PD”

Energy (RwB97XD): -1590.32319016 A.U.

GIbbs Free Energy: -1589.904166 A.U.

Pd	2.058950	0.942095	-0.375661
P	3.425248	-0.813609	0.101746
C	4.996872	-1.065095	-0.829621
H	5.672537	-0.224259	-0.644450
H	5.494873	-1.997602	-0.536777
H	4.783402	-1.096213	-1.902574
C	2.580463	-2.427794	-0.173535
H	2.270593	-2.508476	-1.219961
H	3.232328	-3.273299	0.079121
H	1.676178	-2.470157	0.442330
C	4.014544	-1.026242	1.836172
H	4.595085	-1.949560	1.953224
H	4.634148	-0.171828	2.124869
H	3.151871	-1.064048	2.508604
C	-0.171325	1.470851	0.087497
C	-0.116384	2.179610	1.318210
C	0.315776	2.153776	-1.060585
C	0.442884	3.442349	1.417299
H	-0.577119	1.742352	2.197762
C	0.922827	3.425885	-0.936924
H	0.073274	1.799039	-2.056368
C	0.993584	4.067383	0.290893
H	0.447478	3.948646	2.378306
H	1.290924	3.915128	-1.833913
H	1.449244	5.049570	0.371276
C	-1.102835	0.261172	0.009523
C	-1.028528	-0.709064	-1.207242
C	-2.568947	0.771600	0.018985
C	-0.976332	-0.723332	1.202811
C	-0.078876	-0.775258	-2.220877
C	-2.037051	-1.689434	-1.176262
C	-3.533461	-0.239450	0.056766
C	-2.977390	2.099939	-0.033195
C	-1.975301	-1.706275	1.224546
C	0.043058	-0.778038	2.147242
C	-0.167724	-1.759565	-3.211116
H	0.772192	-0.102405	-2.235089
C	-2.129126	-2.665065	-2.155870
C	-2.932936	-1.625192	0.049974
C	-4.887872	0.058801	0.064352
C	-4.341408	2.406163	-0.029595
H	-2.251858	2.905725	-0.075858
C	-1.989022	-2.694790	2.197097
C	0.034967	-1.775273	3.126973
H	0.858655	-0.063693	2.117522
C	-1.194121	-2.695008	-3.192928
H	0.583694	-1.788683	-3.995703
H	-2.918440	-3.411290	-2.103564
H	-3.685640	-2.417933	0.062483
C	-5.294648	1.394110	0.024504
H	-5.622543	-0.742448	0.095125
H	-4.654312	3.445728	-0.069368
C	-0.980620	-2.724942	3.162995
H	-2.771760	-3.449702	2.193571
H	0.833935	-1.806126	3.863209
H	-1.259998	-3.455524	-3.965834
H	-6.352808	1.639996	0.029634
H	-0.982766	-3.496116	3.928119

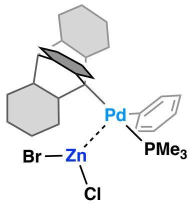

IM$_{Zn}$2'

Energy (RwB97XD): -2291.12511396 A.U.

Gibbs Free Energy: -2290.712038 A.U.

Pd	0.791702	0.354480	0.522404
P	3.133539	0.455666	1.007090
C	4.082867	-1.091876	1.263064
H	4.075855	-1.687082	0.344764
H	5.120112	-0.858802	1.528278
H	3.627562	-1.686143	2.059409
C	3.446699	1.378859	2.561938
H	2.950022	0.877127	3.398242
H	4.520794	1.441145	2.768926
H	3.042116	2.390941	2.470861
C	4.158827	1.351639	-0.216040
H	5.205910	1.373161	0.105180
H	4.089032	0.846269	-1.184361
H	3.790369	2.374435	-0.331320
C	0.959242	2.233255	-0.091837
C	0.911208	3.261615	0.857788
C	1.199696	2.568896	-1.427391
C	1.095129	4.592443	0.479595
H	0.696184	3.034564	1.899484
C	1.377352	3.899973	-1.806727
H	1.258405	1.798159	-2.191006
C	1.326646	4.918076	-0.855975
H	1.043281	5.376040	1.231516
H	1.555805	4.138794	-2.852097
H	1.462978	5.953959	-1.153170
C	-1.311640	0.292771	0.107212
C	-1.490197	-0.659592	-1.079868
C	-2.395088	1.349563	-0.236662
C	-1.904565	-0.370031	1.368415
C	-0.583650	-0.833987	-2.137619
C	-2.733195	-1.298668	-1.170176
C	-3.678995	0.776453	-0.386106
C	-2.254265	2.718251	-0.419254
C	-3.162409	-0.960655	1.208834
C	-1.348562	-0.333231	2.644407
C	-0.845688	-1.761693	-3.167673
H	0.211271	-0.096125	-2.273931
C	-2.986620	-2.228781	-2.167469
C	-3.728002	-0.739667	-0.176122
C	-4.781847	1.553918	-0.696531
C	-3.369520	3.505495	-0.731464
H	-1.285827	3.187573	-0.321051
C	-3.817938	-1.576576	2.265444
C	-2.001827	-0.940216	3.718396
H	-0.396351	0.166419	2.814173
C	-2.022619	-2.496673	-3.148012
H	-0.129118	-1.878293	-3.975656
H	-3.949934	-2.732541	-2.204336
H	-4.733361	-1.150456	-0.302512
C	-4.627470	2.933423	-0.869606
H	-5.761228	1.092697	-0.804422
H	-3.240301	4.575832	-0.867733
C	-3.224879	-1.578239	3.529093
H	-4.794086	-2.031729	2.113238
H	-1.550514	-0.912706	4.706325
H	-2.223817	-3.229259	-3.923316
H	-5.488004	3.550452	-1.112831
H	-3.727935	-2.054966	4.365641
Zn	1.231612	-1.733059	-1.131437
Br	0.775904	-2.520359	1.017485
Cl	2.904252	-1.740273	-2.496002

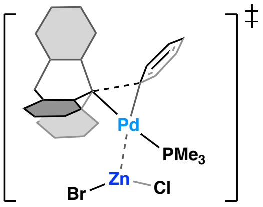

TS$_{Zn}$2

Energy (RwB97XD): -2291.09938524 A.U.

Gibbs Free Energy: -2290.687782 A.U.

Pd	0.681855	0.604819	0.450150
P	2.773150	1.542244	0.849726
C	4.309619	0.589248	1.164056
H	4.623554	0.078683	0.248097
H	5.118489	1.251049	1.493622
H	4.121700	-0.168150	1.931011
C	2.632895	2.581918	2.358779
H	2.439248	1.947340	3.229199
H	3.551209	3.155101	2.530534
H	1.793730	3.274740	2.239674
C	3.271899	2.755354	-0.427250
H	4.155260	3.323218	-0.114306
H	3.491471	2.226623	-1.359733
H	2.438908	3.442909	-0.606849
C	-0.509724	2.127351	-0.172360
C	-0.939724	2.987373	0.852273
C	-0.375123	2.654060	-1.464625
C	-1.170181	4.338841	0.601099
H	-1.124800	2.604572	1.850769
C	-0.616978	4.004639	-1.712353
H	-0.081166	2.023333	-2.295485
C	-1.008137	4.858549	-0.682294
H	-1.497845	4.982090	1.413850
H	-0.498598	4.387078	-2.722899
H	-1.197786	5.909429	-0.879778
C	-1.454769	0.166949	-0.008018
C	-1.114843	-0.898407	-1.069790
C	-2.914870	0.545647	-0.381118
C	-1.608609	-0.533509	1.366149
C	-0.163738	-0.809622	-2.094322
C	-1.919235	-2.041221	-1.009121
C	-3.747968	-0.594989	-0.374668
C	-3.474967	1.772021	-0.717825
C	-2.424620	-1.670739	1.339311
C	-1.063716	-0.134985	2.586247
C	0.054953	-1.897053	-2.955504
H	0.360600	0.121658	-2.286597
C	-1.697079	-3.123797	-1.848794
C	-3.051547	-1.903970	-0.015572
C	-5.095498	-0.503895	-0.682470
C	-4.836665	1.861921	-1.028107
H	-2.880735	2.673772	-0.748866
C	-2.642883	-2.433915	2.475348
C	-1.281915	-0.896945	3.736857
H	-0.456358	0.763383	2.665696
C	-0.686081	-3.064802	-2.809280
H	0.803881	-1.811003	-3.737153
H	-2.321231	-4.010121	-1.764986
H	-3.733219	-2.758153	-0.029138
C	-5.648751	0.736129	-1.010535
H	-5.715541	-1.397320	-0.669252
H	-5.254457	2.830892	-1.286776
C	-2.057003	-2.049604	3.682893
H	-3.275116	-3.317162	2.425197
H	-0.837973	-0.582195	4.676807
H	-0.503825	-3.912251	-3.462704
H	-6.704677	0.815202	-1.252860
H	-2.218441	-2.642096	4.578704
Zn	2.037039	-1.137331	-0.917792
Br	1.717349	-2.529589	0.905018
Cl	3.449076	-0.591041	-2.462029

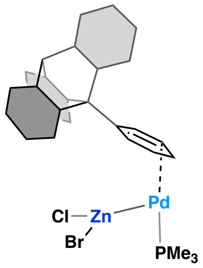

PD*Zn*

Energy (RwB97XD): -2291.19979620 A.U.

Gibbs Free Energy: -2290.784397 A.U.

Pd	1.387686	0.171153	0.990957
P	1.995994	2.301173	0.560420
C	3.777791	2.686781	0.800227
H	4.369211	2.139115	0.059487
H	3.969518	3.760494	0.688489
H	4.094673	2.364244	1.796321
C	1.185634	3.549558	1.644576
H	1.336416	3.287135	2.696453
H	1.593507	4.551083	1.464245
H	0.110391	3.567071	1.437726
C	1.683296	3.030153	-1.096481
H	2.073527	4.053334	-1.147110
H	2.168945	2.425044	-1.868755
H	0.605616	3.056634	-1.285784
C	-2.170420	1.761408	-0.321586
C	-2.514506	2.565766	0.772582
C	-2.108042	2.380232	-1.576335
C	-2.675558	3.943276	0.640569
H	-2.708320	2.111119	1.737650
C	-2.267176	3.757528	-1.715752
H	-1.975393	1.780942	-2.469625
C	-2.526269	4.552671	-0.602604
H	-2.936054	4.536516	1.512854
H	-2.203124	4.204288	-2.704040
H	-2.646681	5.626930	-0.708171
C	-2.111901	0.246706	-0.186504
C	-1.224400	-0.524684	-1.202931
C	-3.544067	-0.321757	-0.354748
C	-1.582834	-0.319041	1.159818
C	-0.254668	-0.012116	-2.057559
C	-1.364982	-1.917336	-1.105637
C	-3.628965	-1.712383	-0.245264
C	-4.696458	0.416500	-0.598863
C	-1.719868	-1.709977	1.261541
C	-0.902229	0.365130	2.172817
C	0.500335	-0.872424	-2.865136
H	-0.048692	1.049234	-2.098993
C	-0.620376	-2.773488	-1.901290
C	-2.292048	-2.363910	0.013896
C	-4.843731	-2.368669	-0.374357
C	-5.923556	-0.240316	-0.729743
H	-4.656214	1.497230	-0.690289
C	-1.262492	-2.399246	2.375883
C	-0.423272	-0.336574	3.291141
H	-0.783158	1.440648	2.143634
C	0.307422	-2.248368	-2.801956
H	1.243968	-0.456519	-3.538985
H	-0.734619	-3.848940	-1.795055
H	-2.352453	-3.451478	0.100556
C	-6.000440	-1.625067	-0.618218
H	-4.890700	-3.451464	-0.286840
H	-6.821778	0.339984	-0.920734
C	-0.619611	-1.709060	3.402712
H	-1.374775	-3.478912	2.427247
H	0.091366	0.208143	4.077331
H	0.901310	-2.911343	-3.423804
H	-6.957465	-2.128477	-0.721320
H	-0.242835	-2.245804	4.267686
Zn	2.795251	-0.645880	-0.759119
Br	1.982000	-2.416563	0.714652
Cl	4.159598	-0.192270	-2.3567

5. Copies for NMR spectra

9-(4,4,5,5-Tetramethyl-1,3,2-dioxaborolane)triptycene (1a):

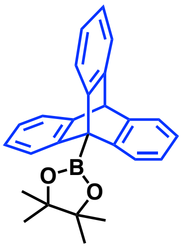

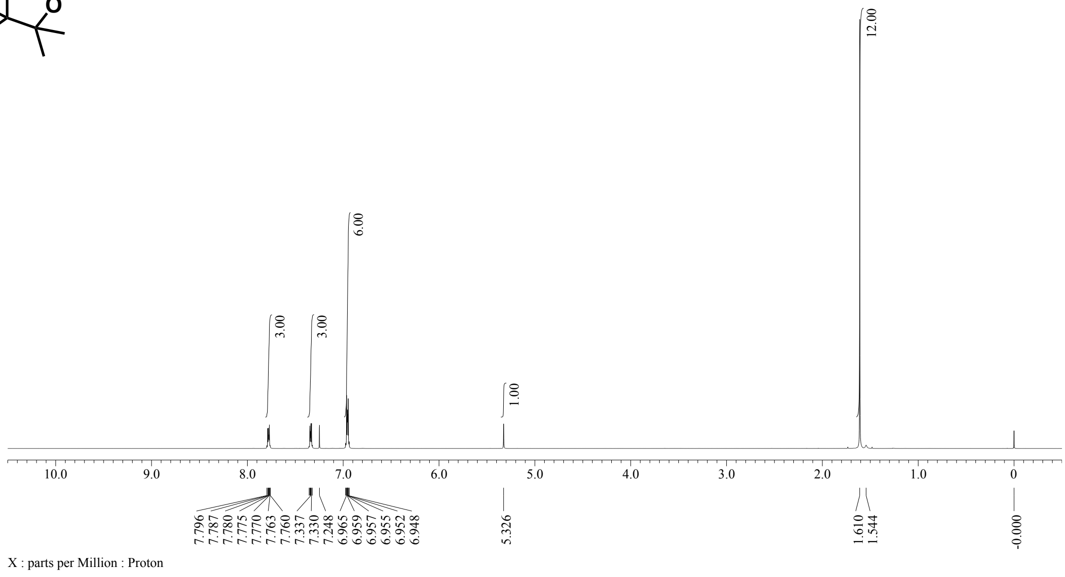

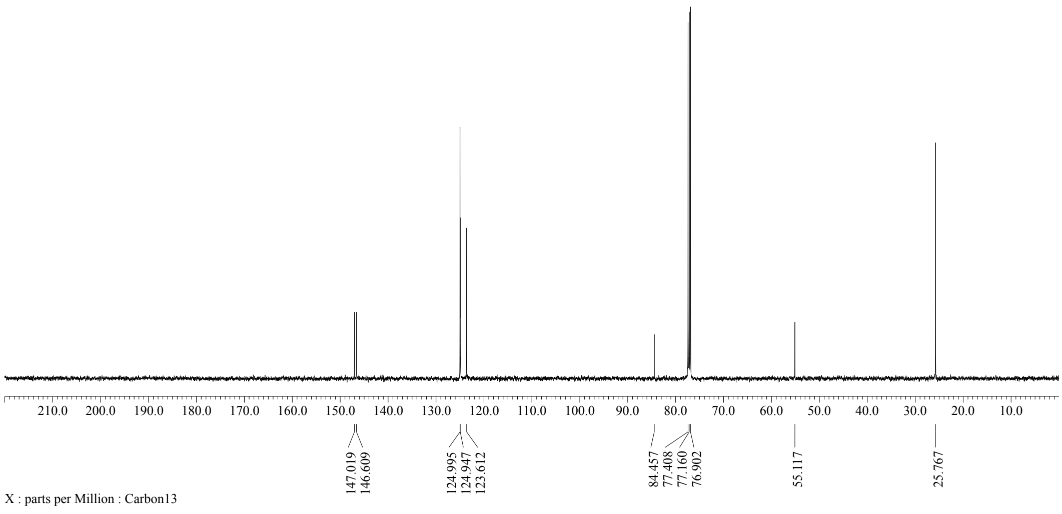

9-(4-Methoxycarbonyl)phenyltriptycene (3):

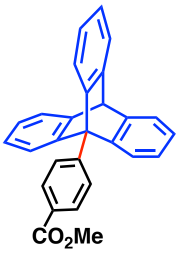

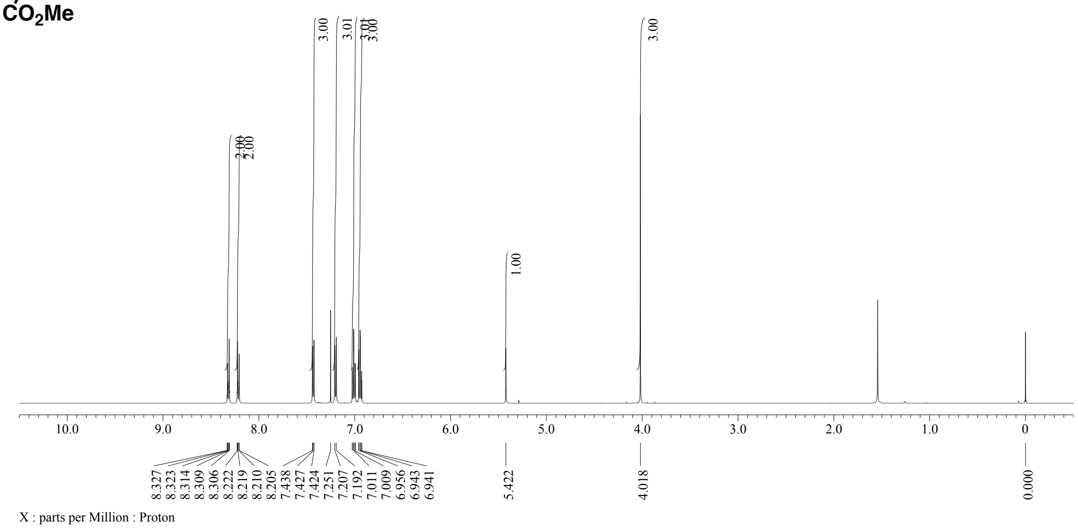

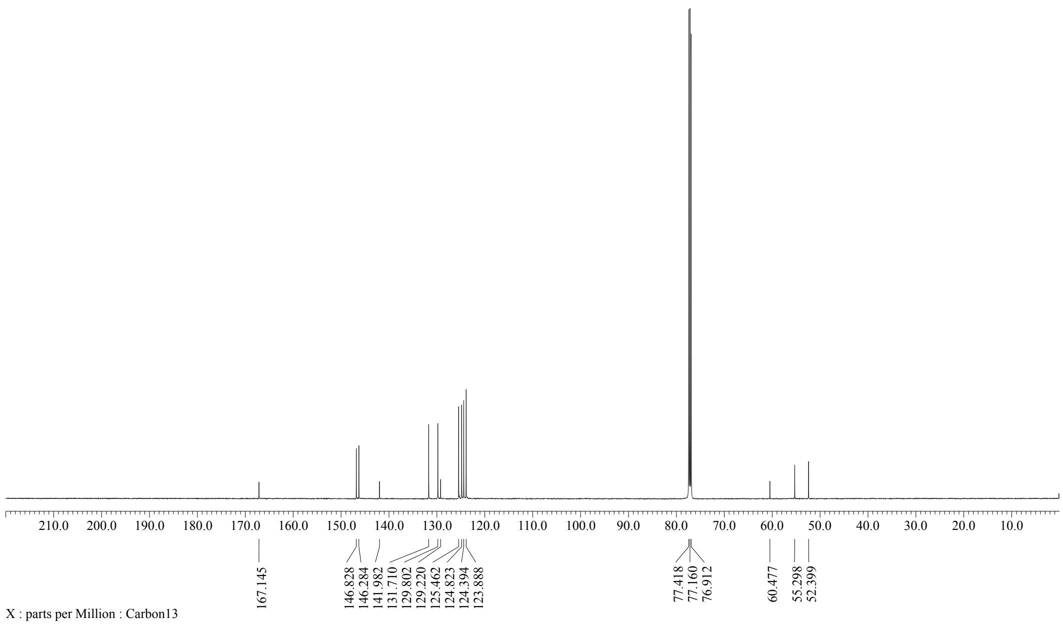

9-(4-Trifluoromethyl)phenyltriptycene (4):

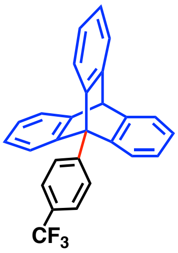

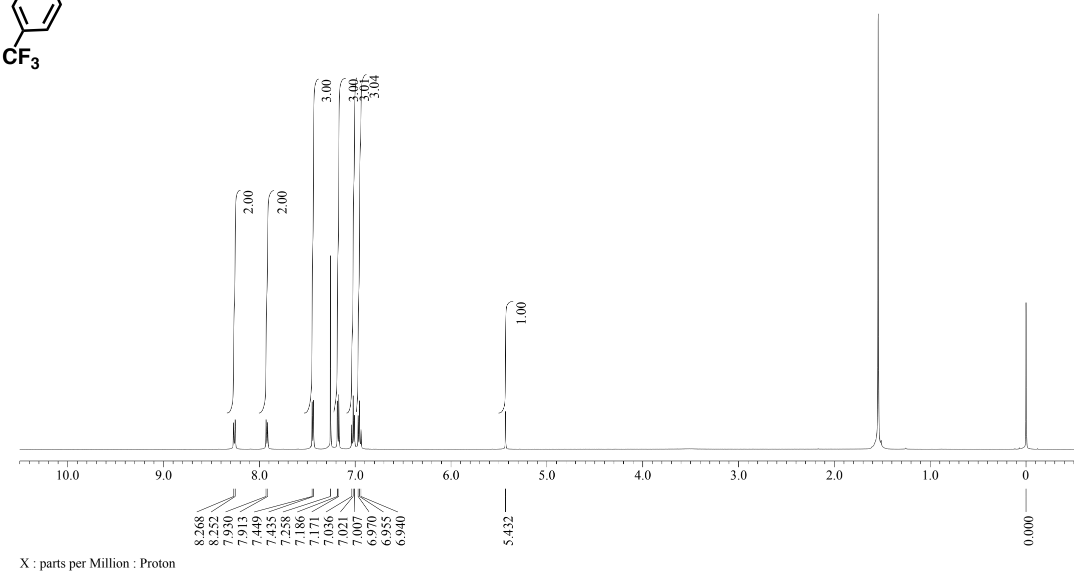

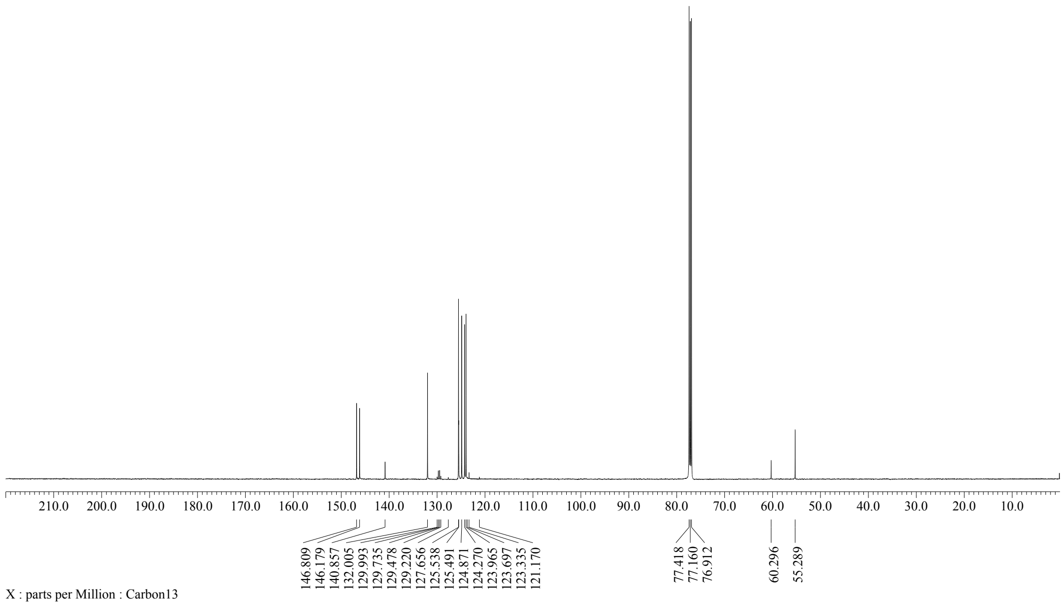

9-(4-Cyano)phenyltriptycene (5):

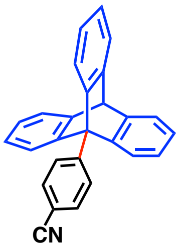

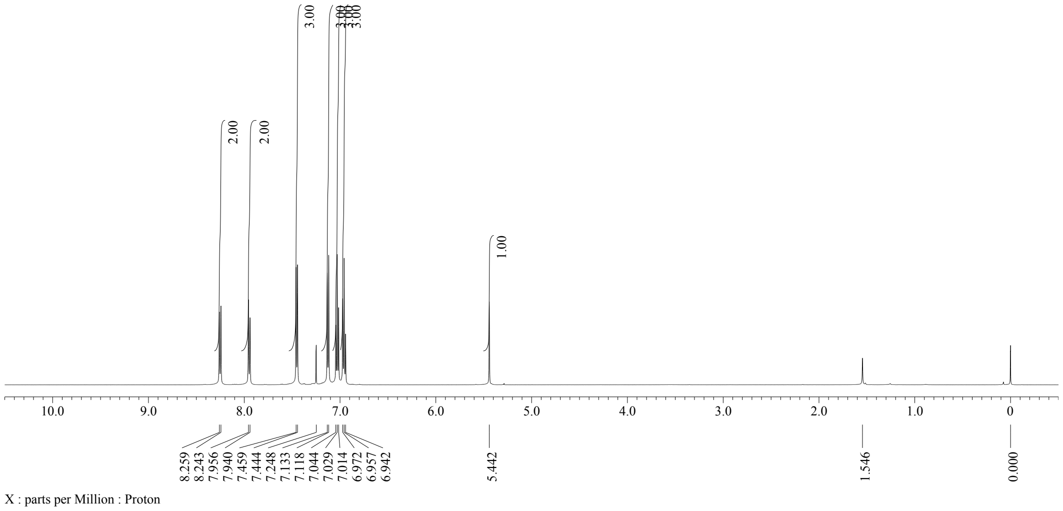

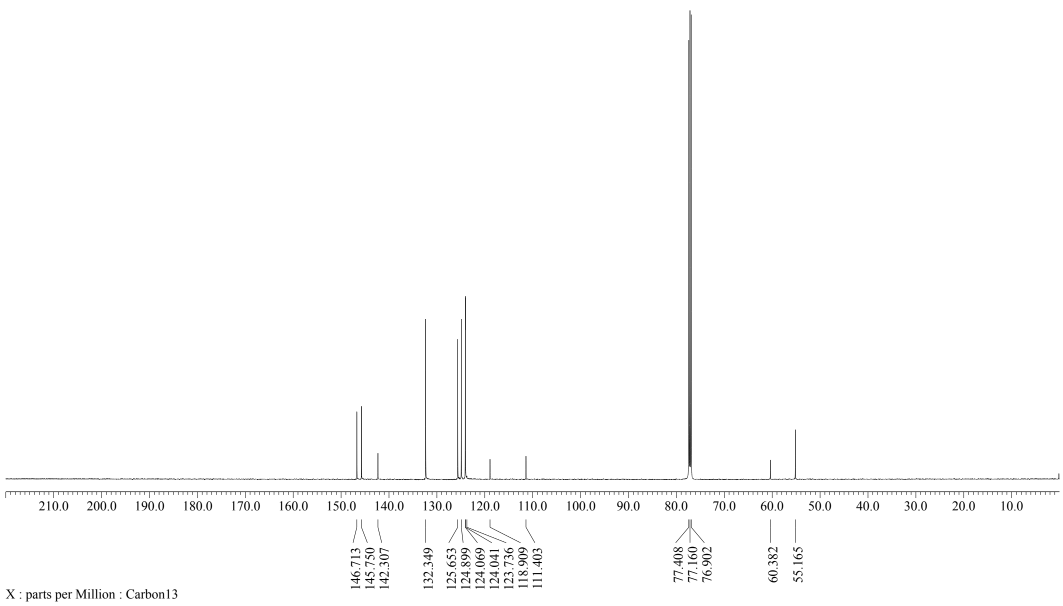

1-{4-(9-Triptycenyl)phenyl}ethan-1-one (6):

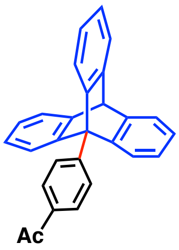

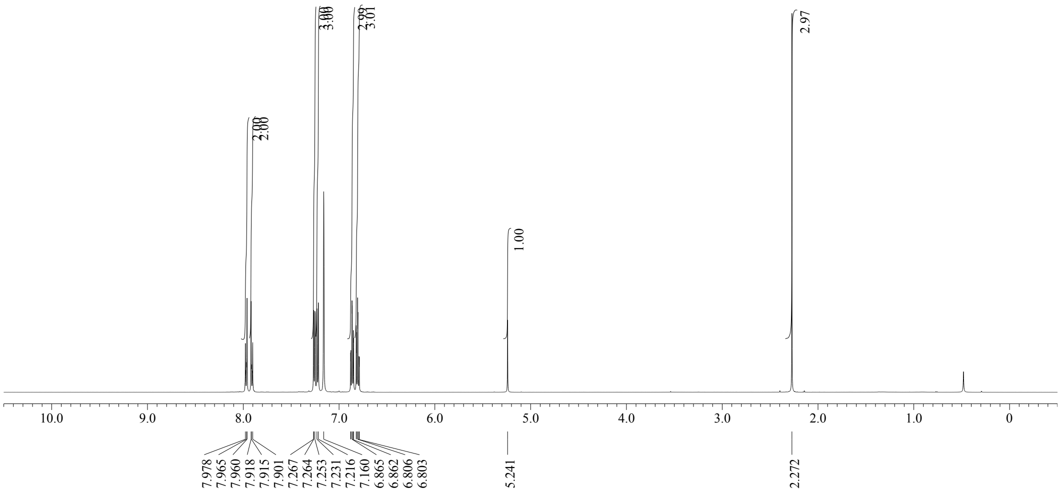

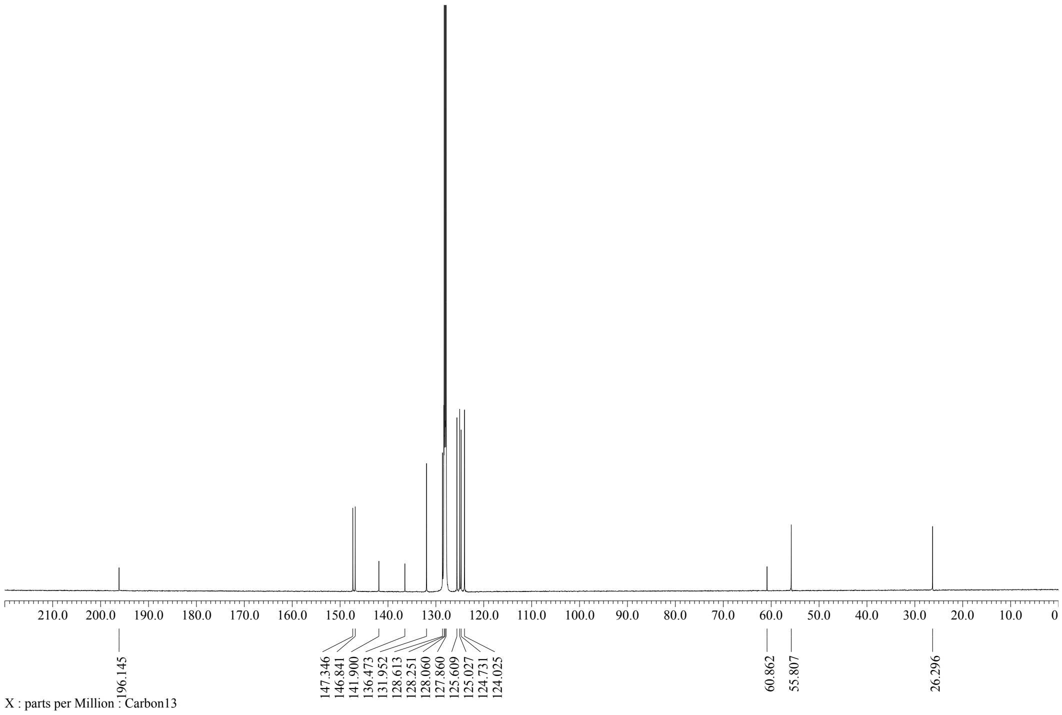

9-Phenyltriptycene (7):

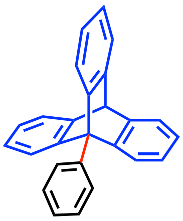

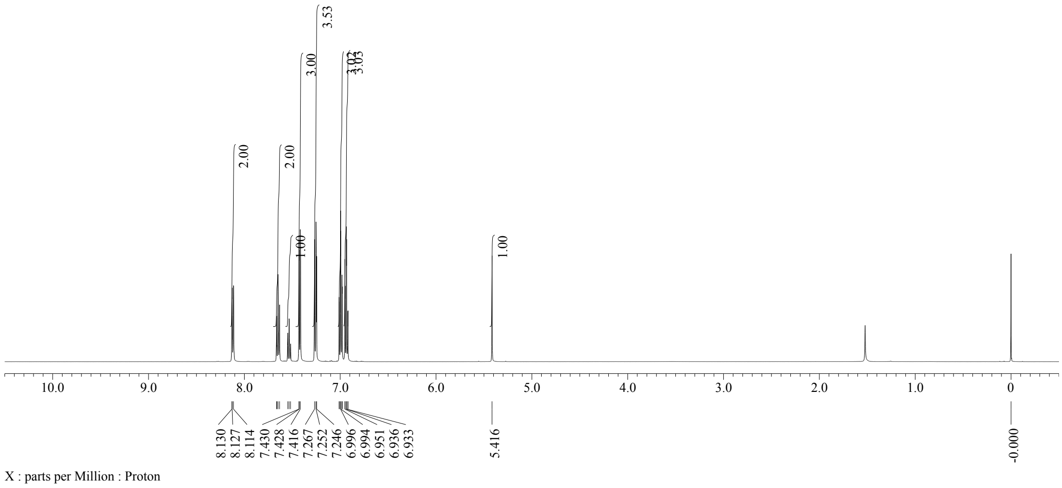

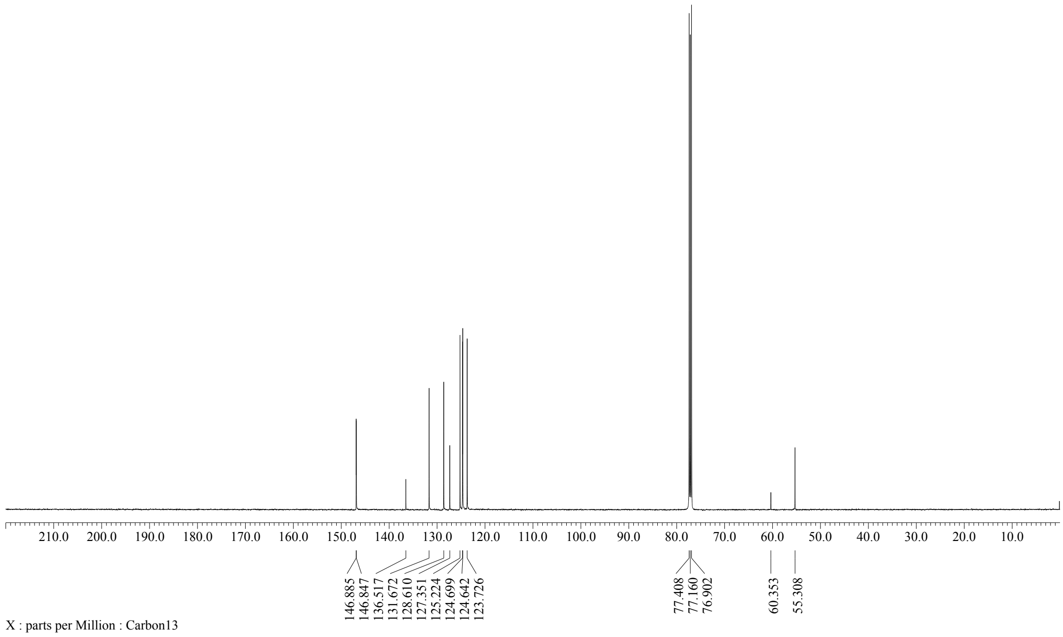

9-(4-Methoxyphenyl)triptycene (8):

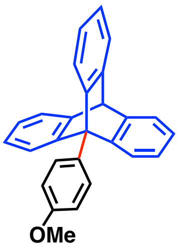

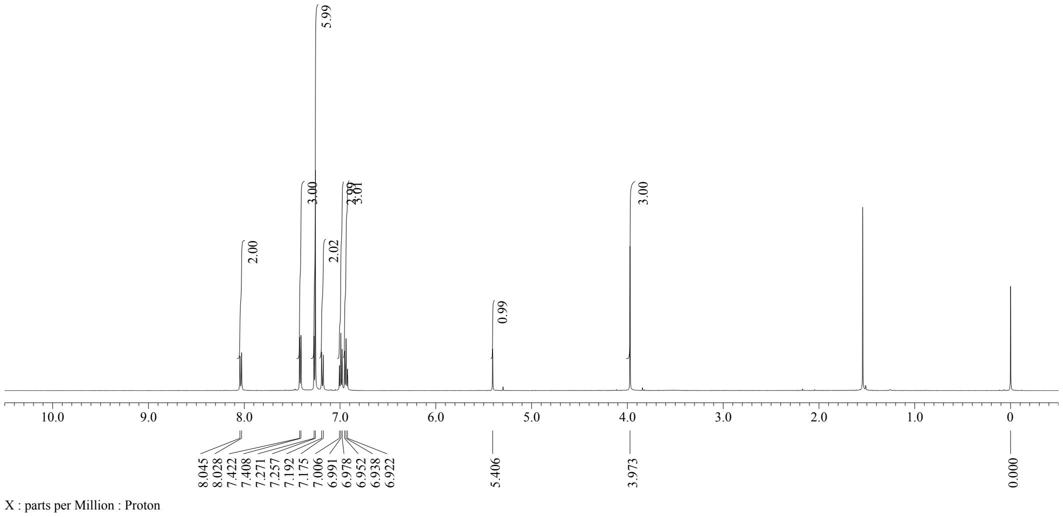

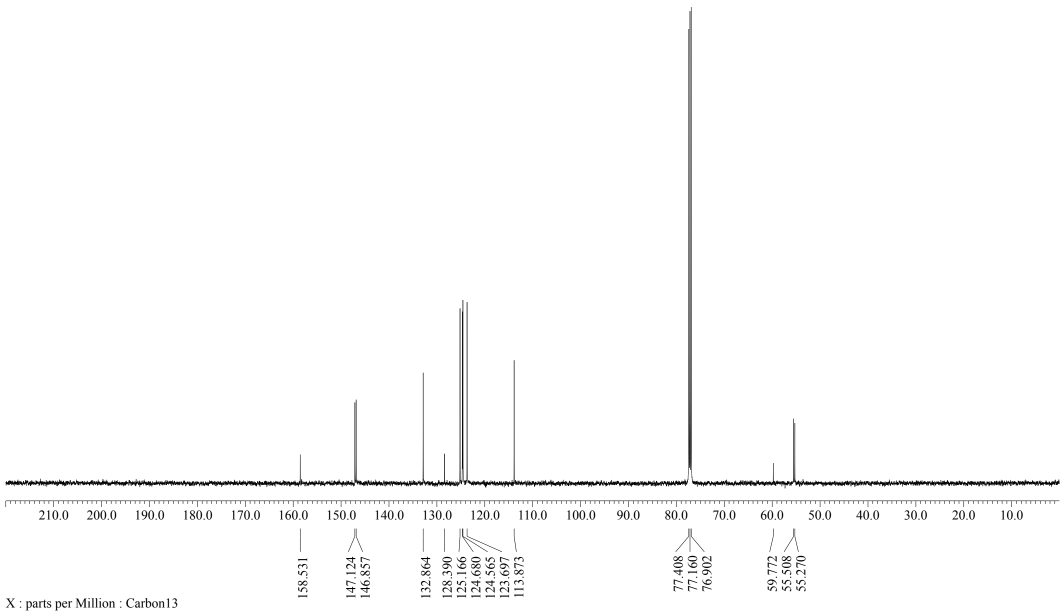

9-(2-Methoxycarbonylphenyl)triptycene (9):

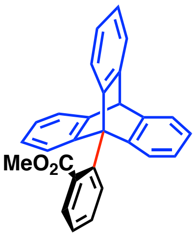

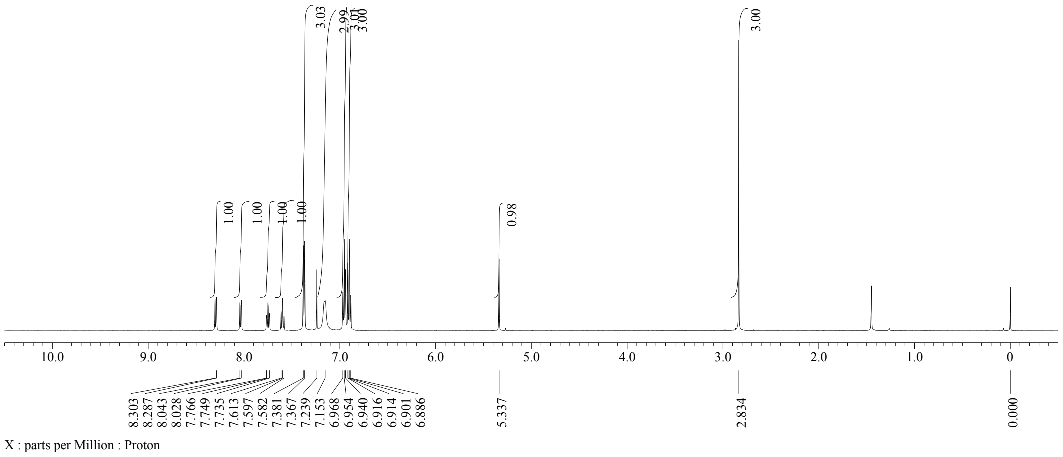

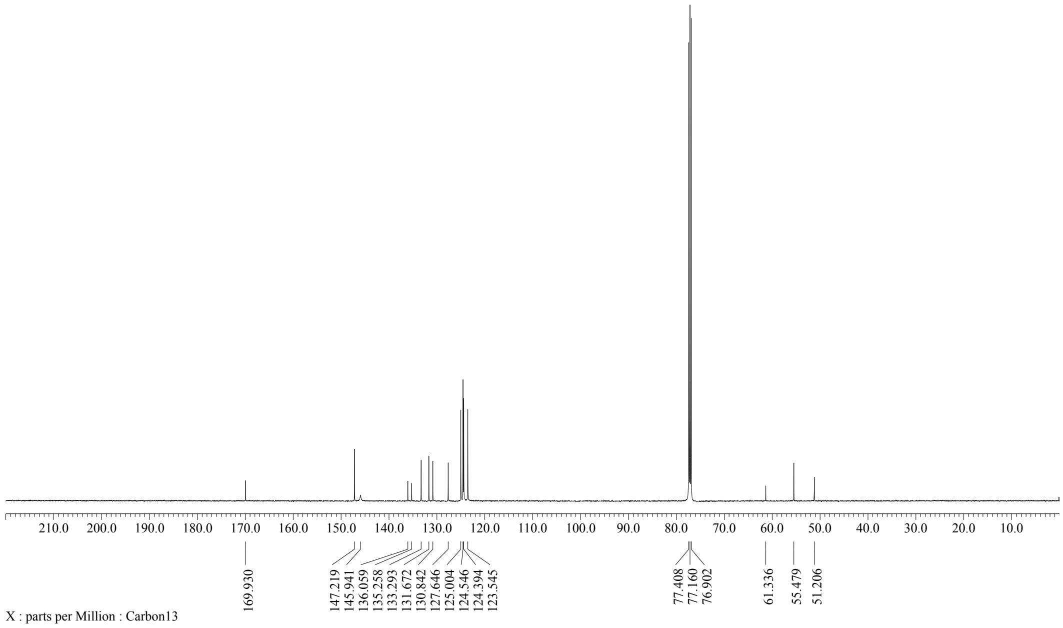

9-(2-Methoxyphenyl)triptycene (10):

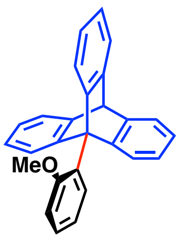

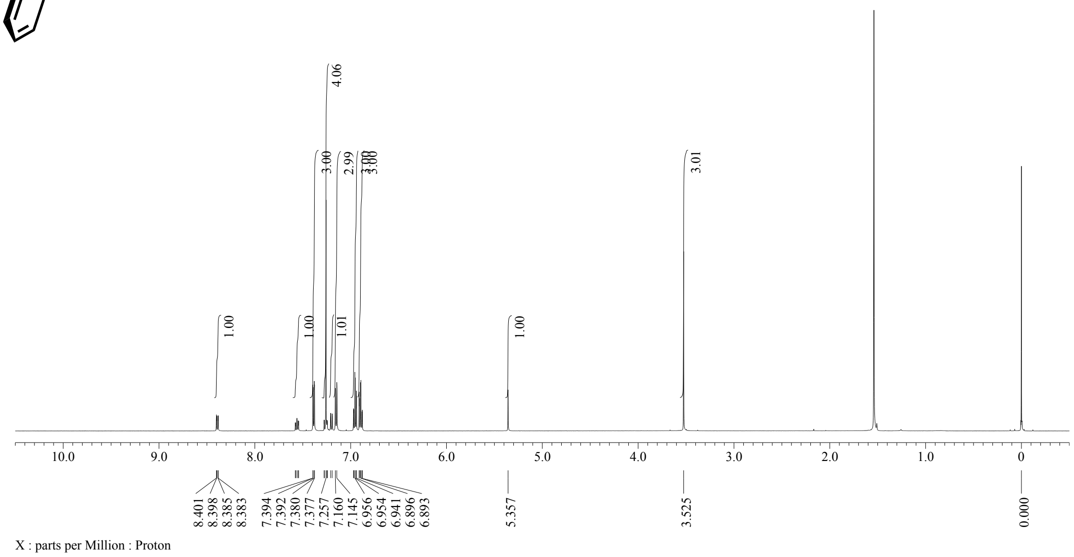

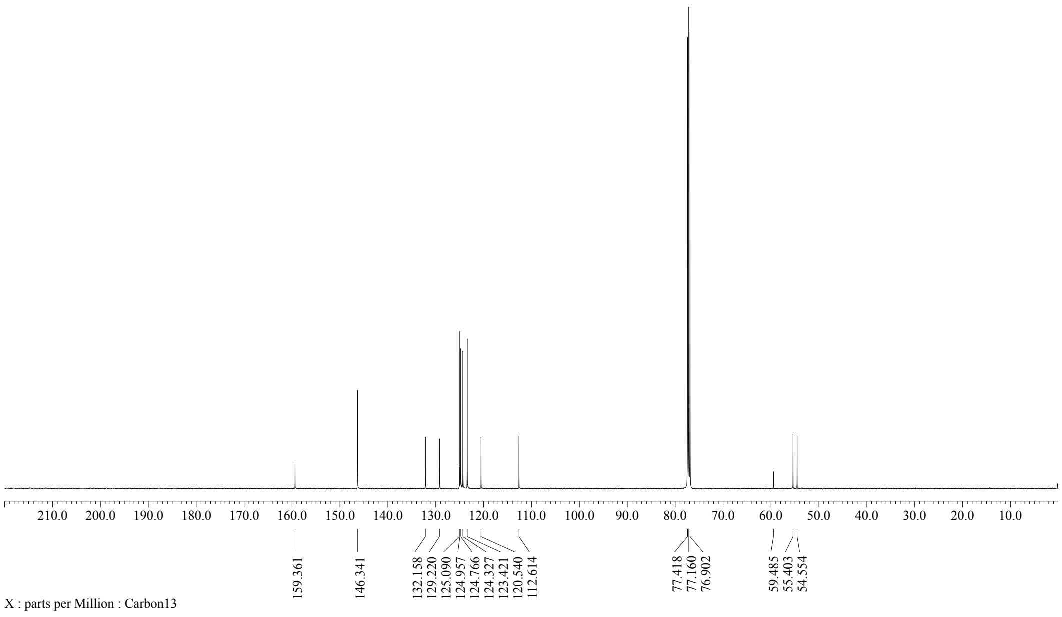

1-{5-(9-Triptycenyl)thiophen-2-yl}ethan-1-one (12):

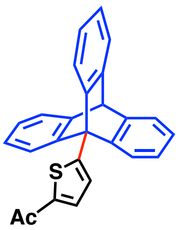

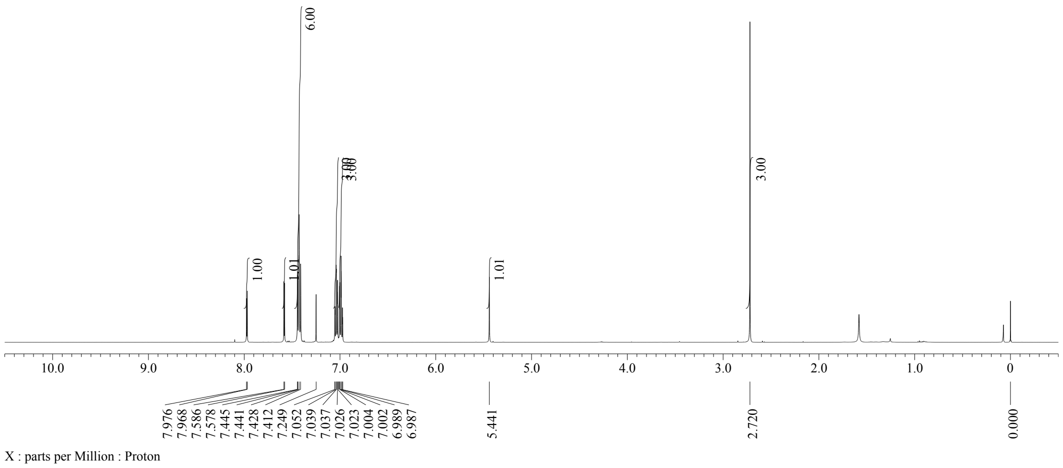

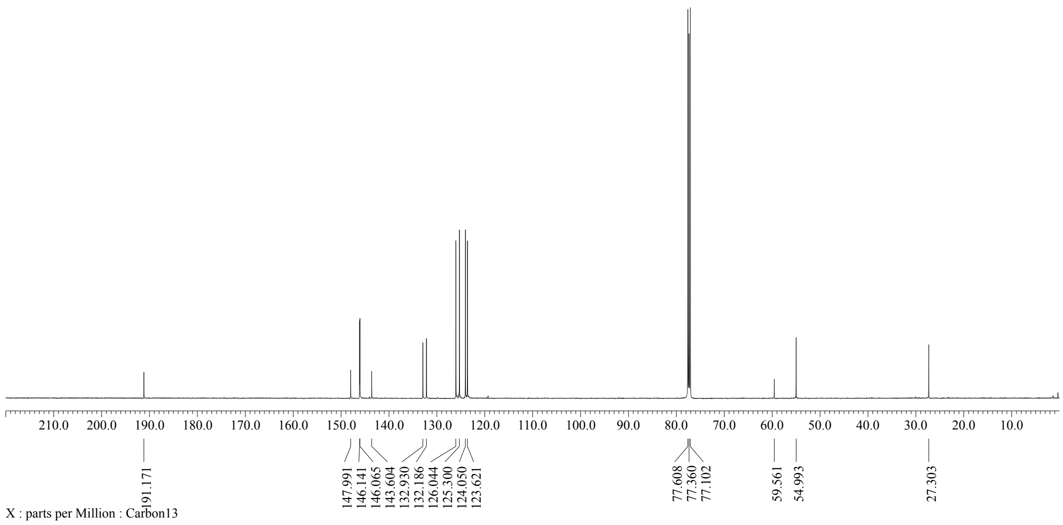

Ethyl 5-(9-triptycenyl)thiophene-2-carboxylate (13):

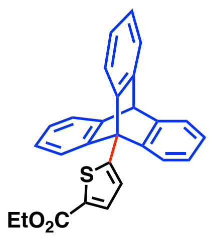

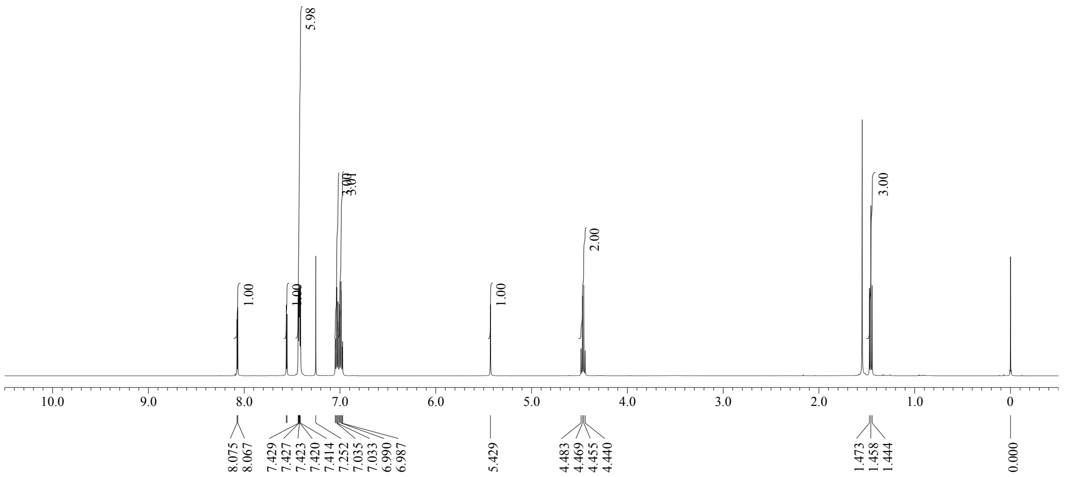

X : parts per Million : Proton

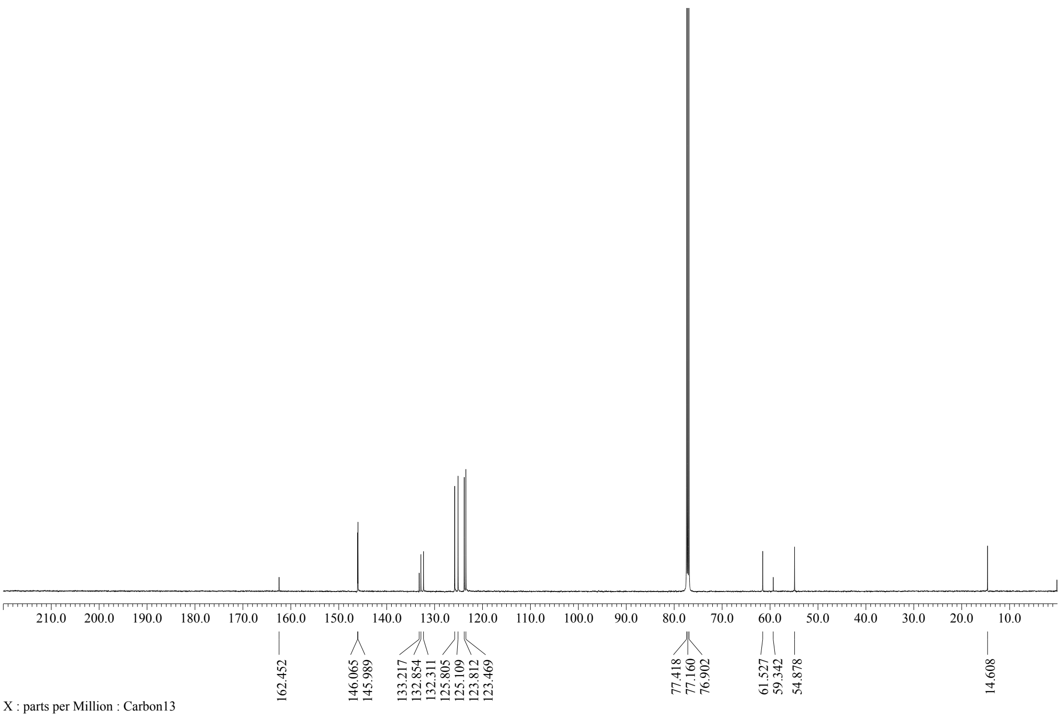

X : parts per Million : Carbon13

2-(9-Triptycenyl)pyridine (14):

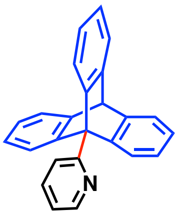

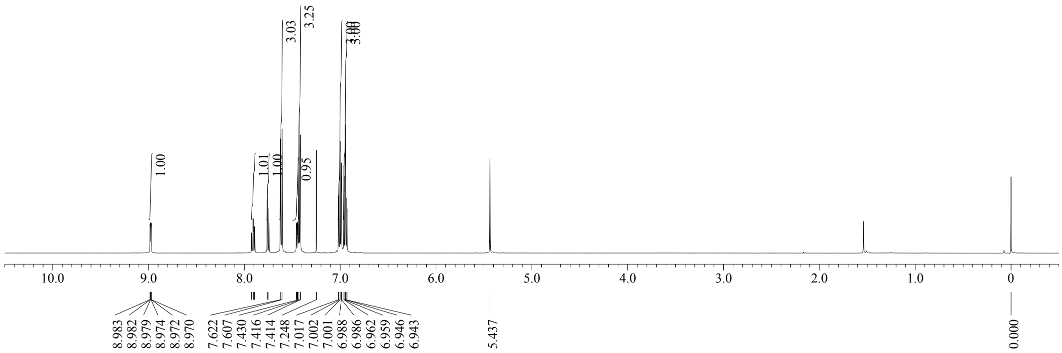

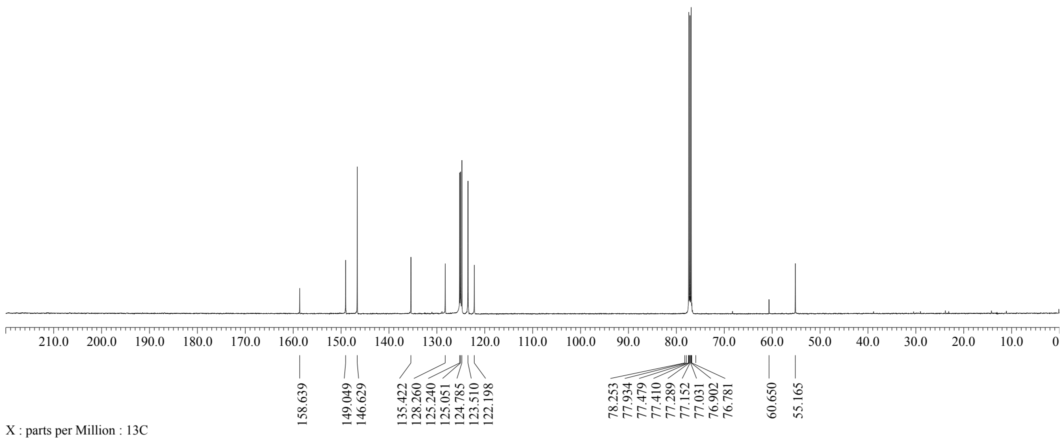

3-(9-Triptycenyl)pyridine (15):

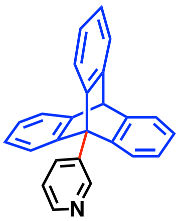

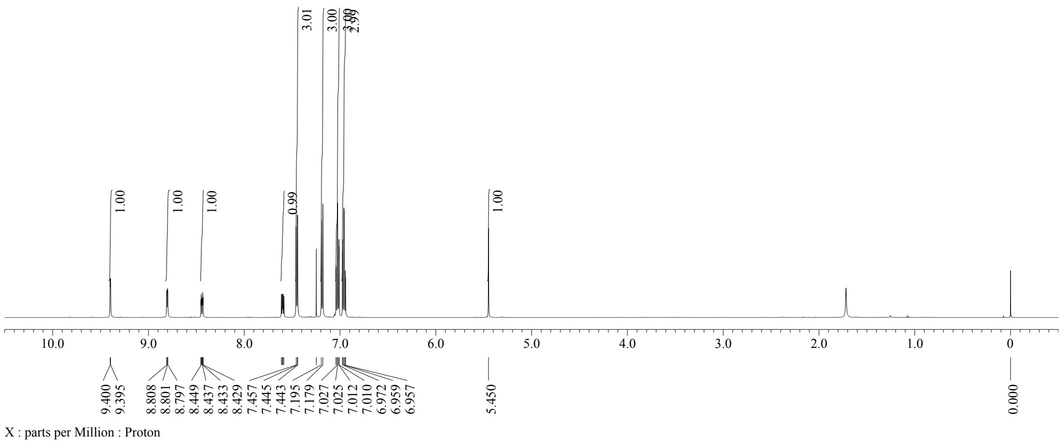

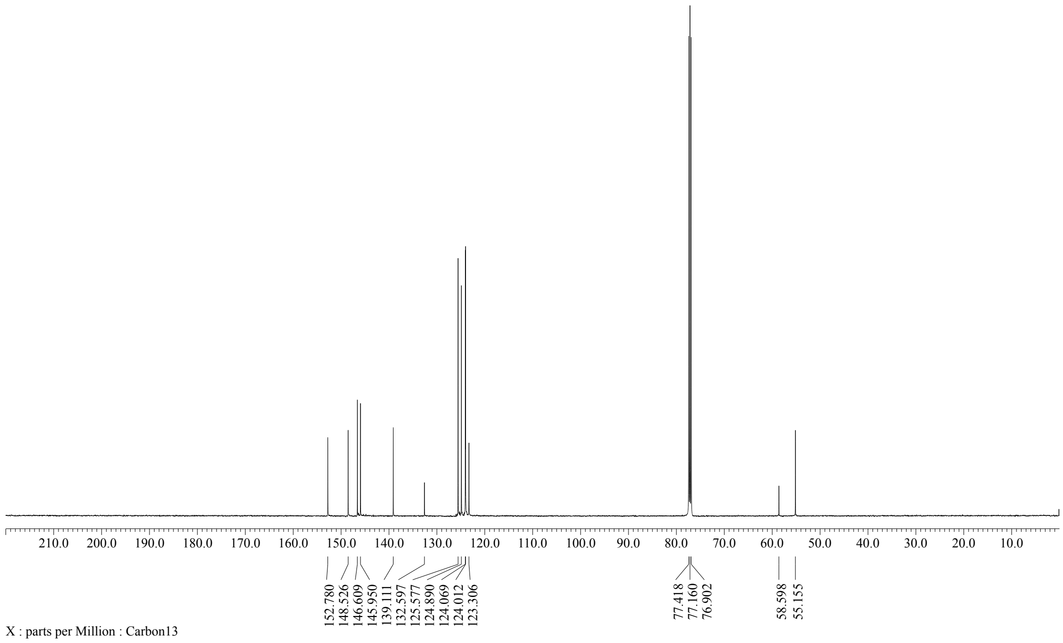

4-(9-Triptycenyl)pyridine (16):

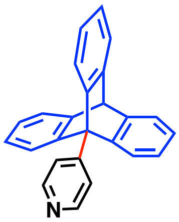

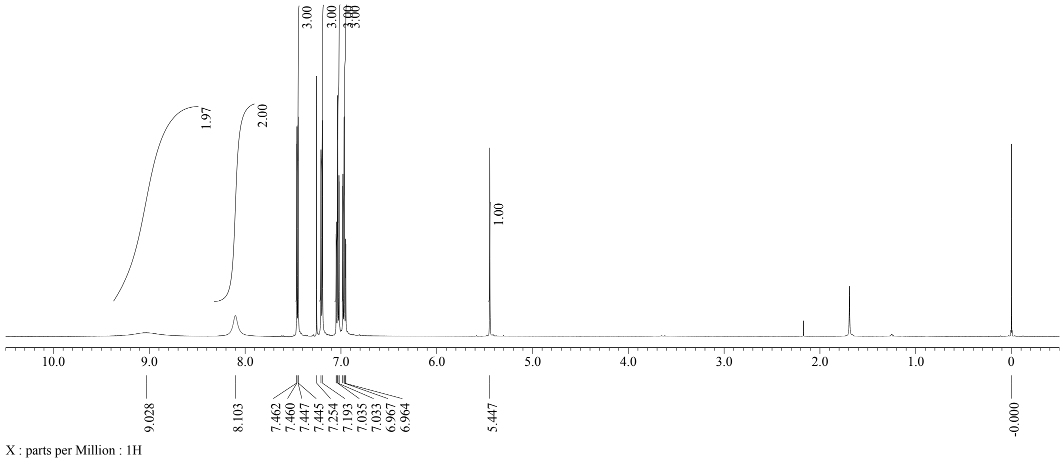

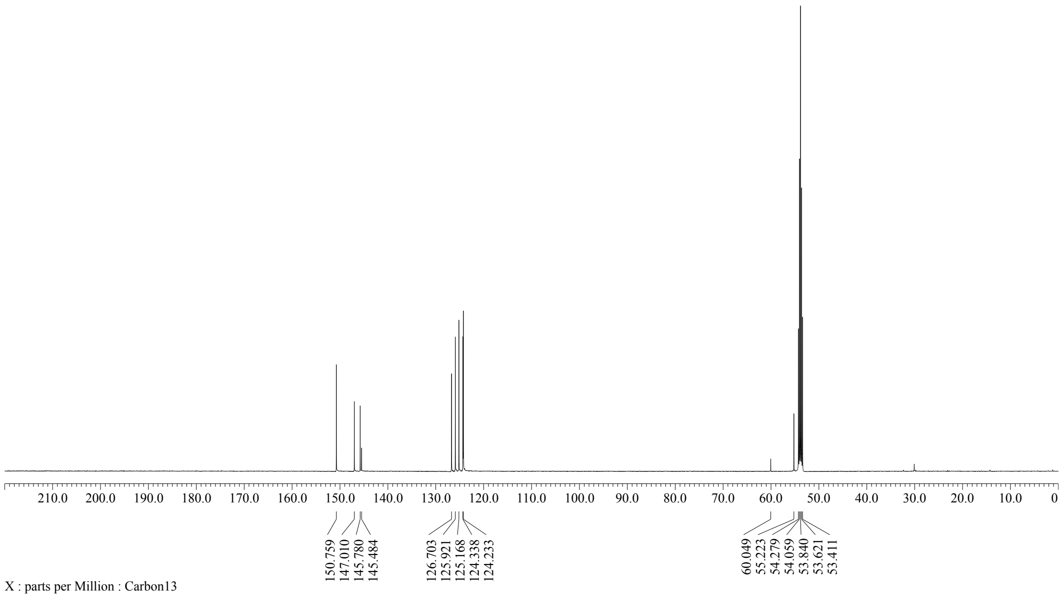

2-(9-Triptycenyl)quinoline (17):

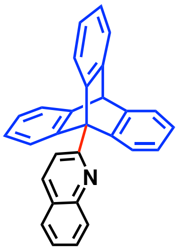

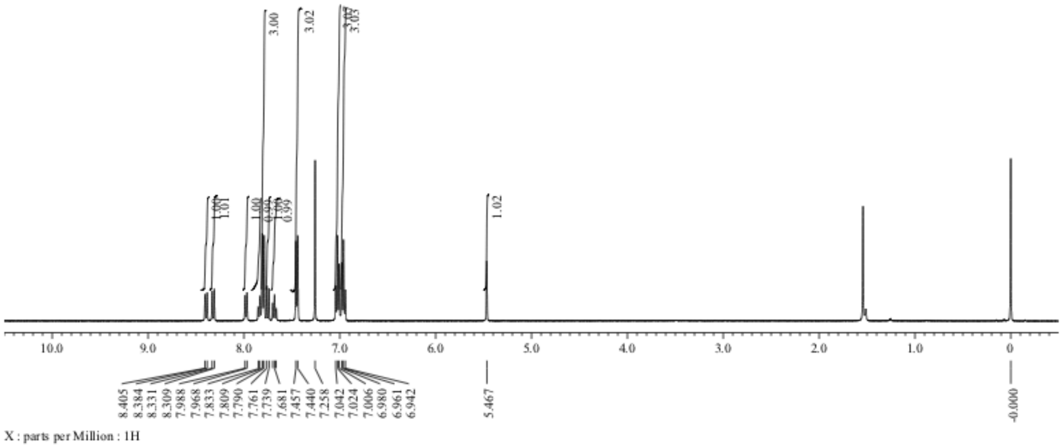

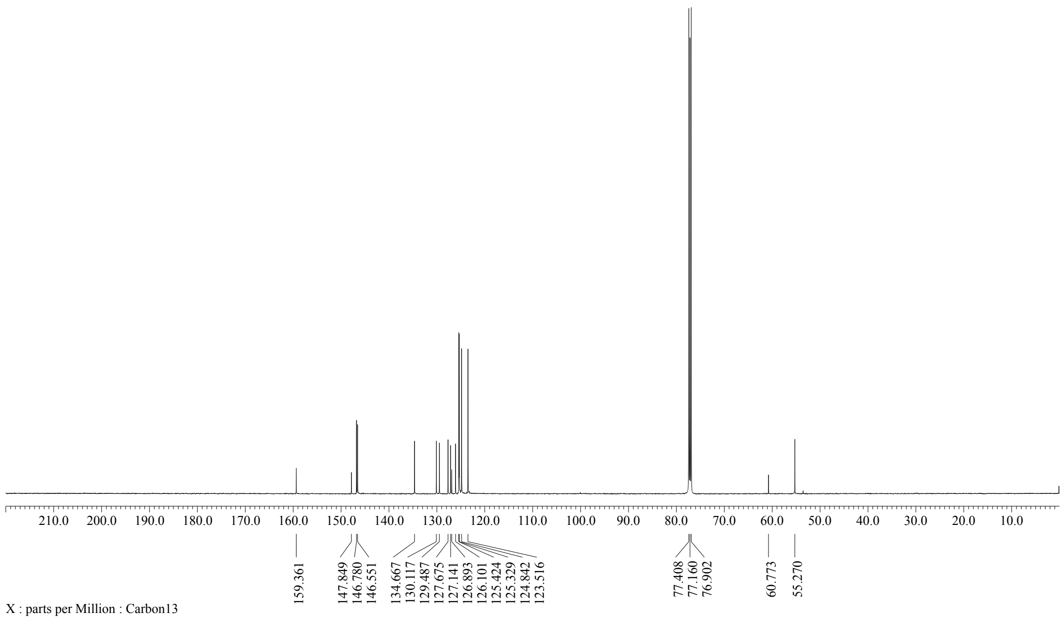

3-(9-Triptycenyl)quinoline (18):

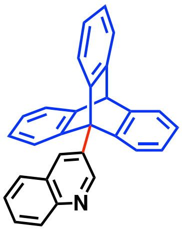

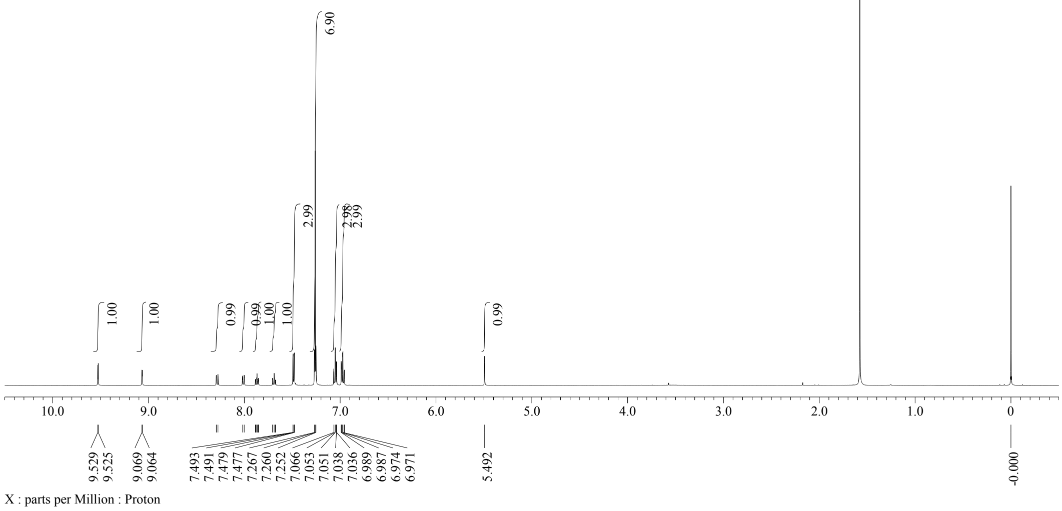

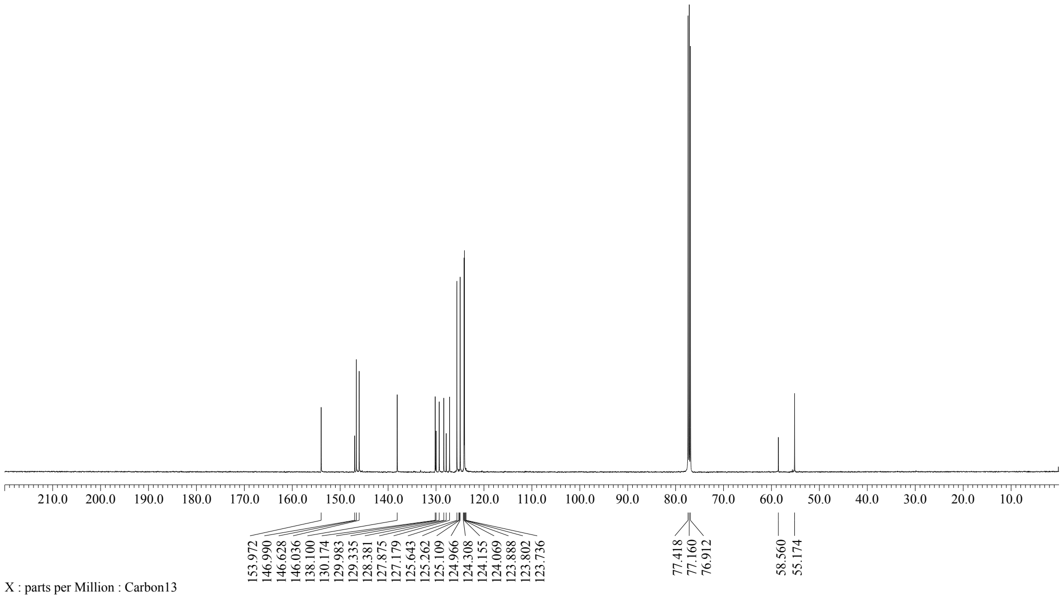

9-(Phenylethynyl)triptycene (19):

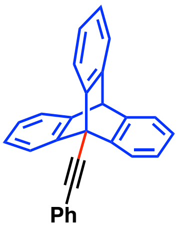

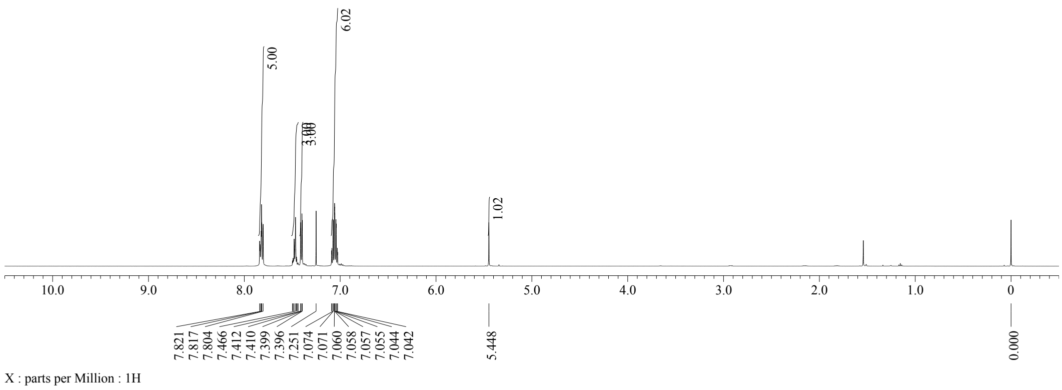

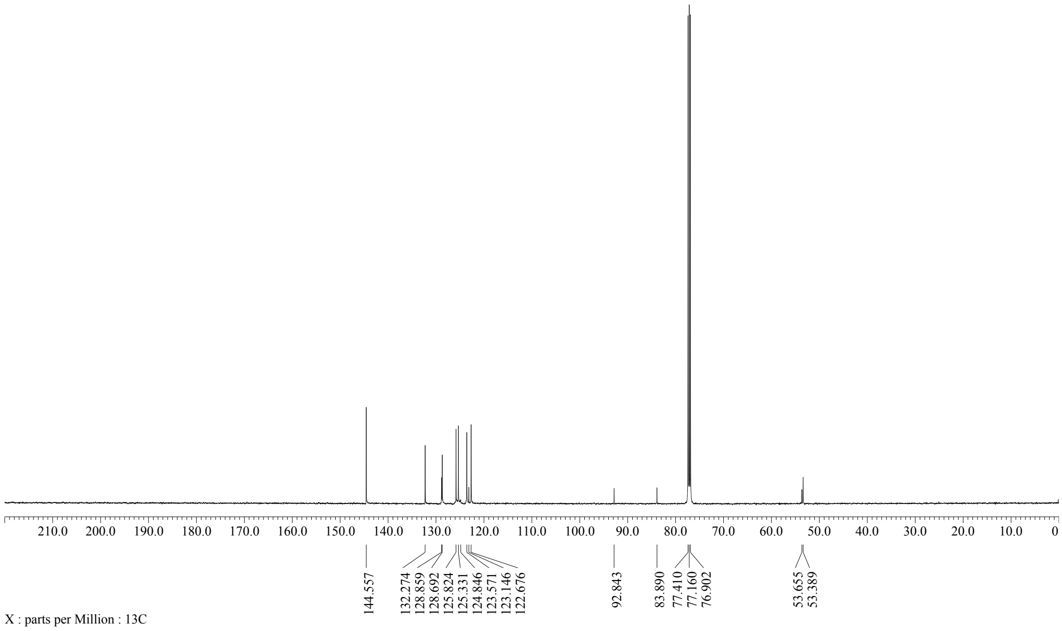

Methyl (9-triptycenyl)acetate (20):

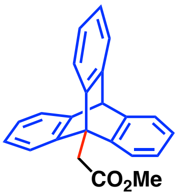

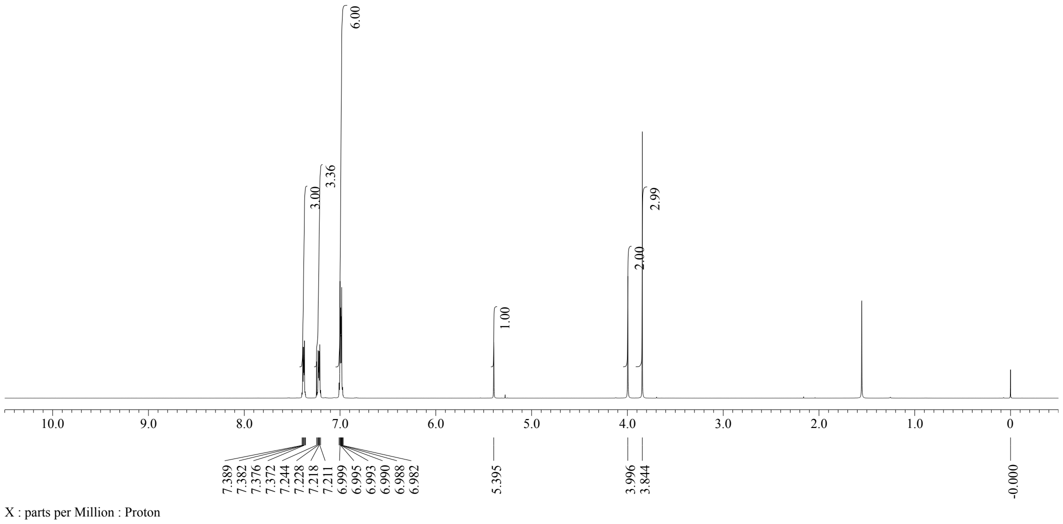

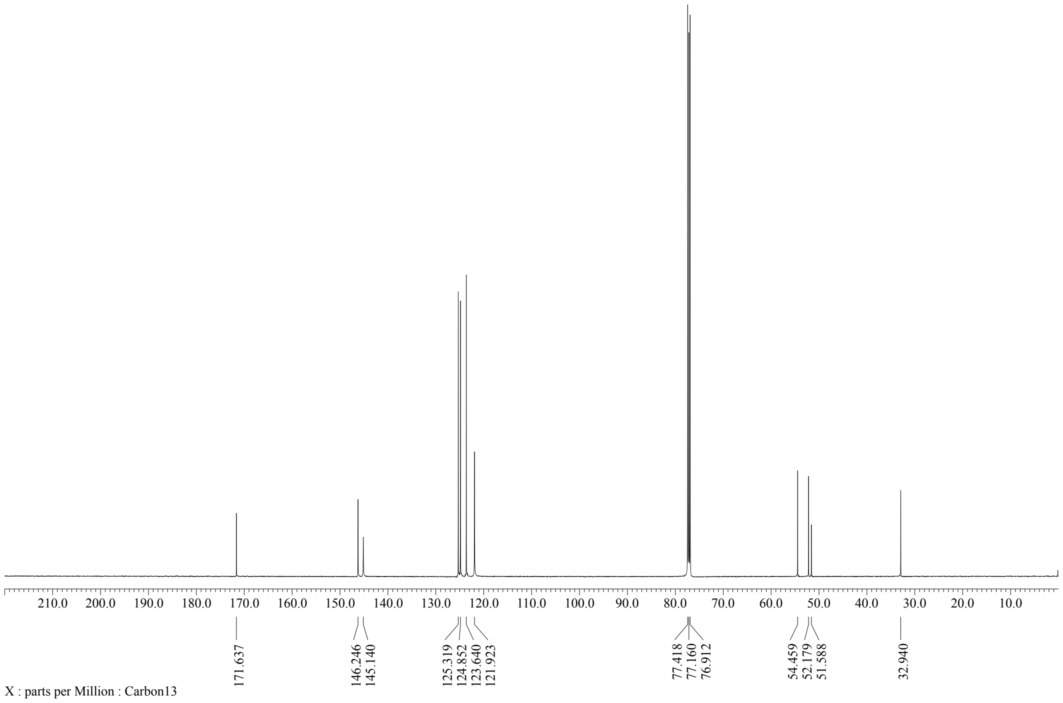

5-(2,2-Dimethyl-1,3-dioxolan-4-yl)-2,2-dimethyltetrahydrofuro[2,3-*d*] [1,3]dioxol-6-yl-4-(adamantan-1-yl)benzoate (22):

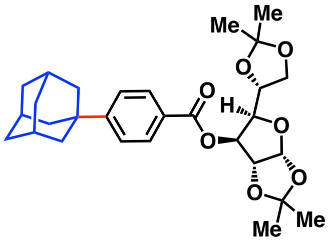

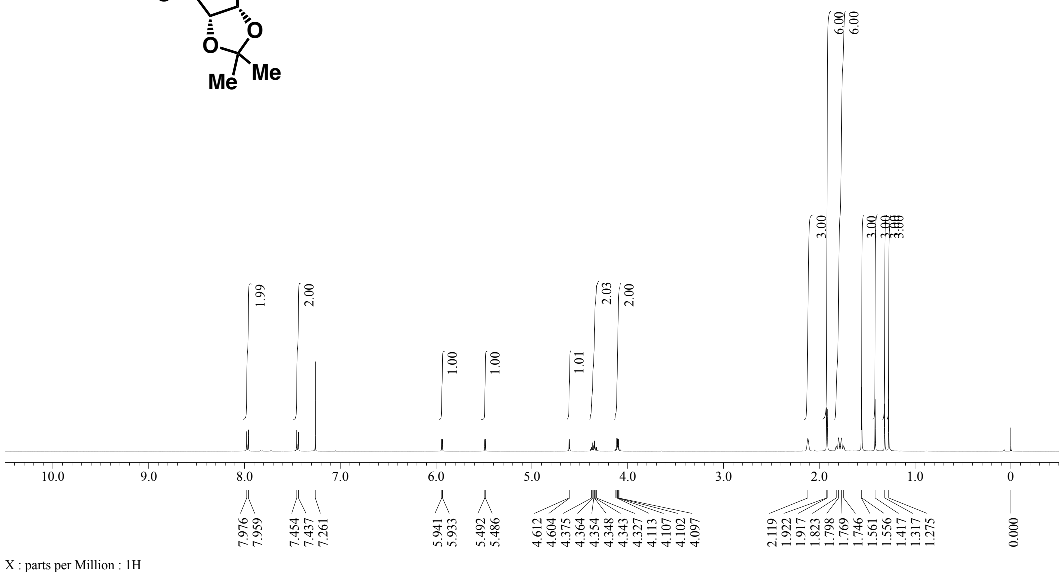

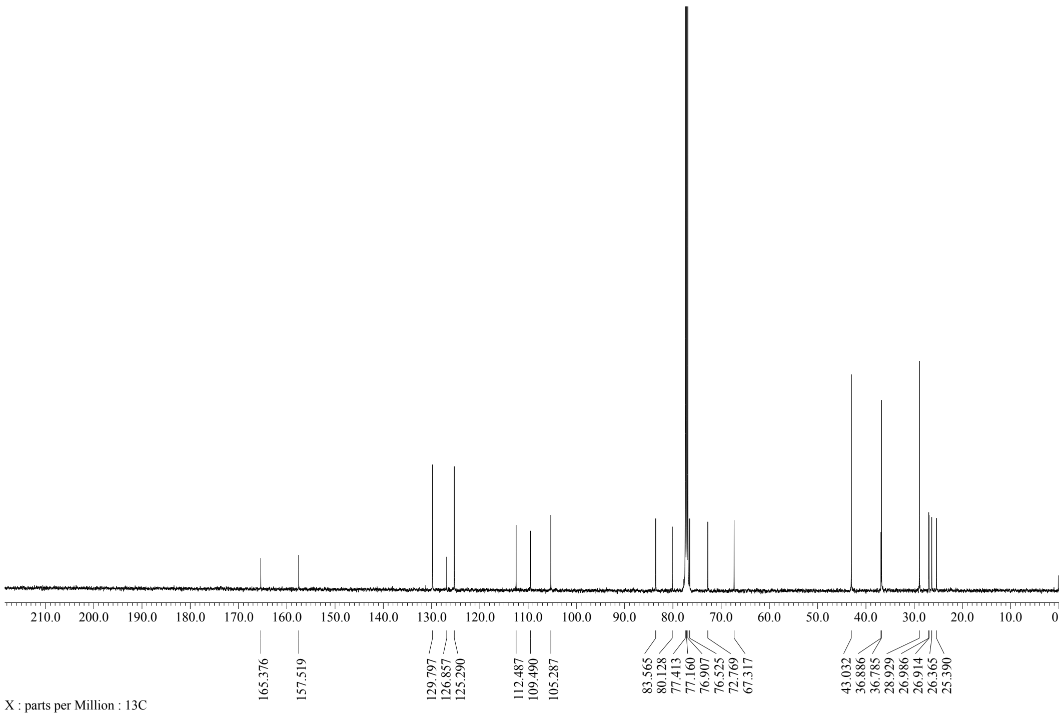

(8*R*,9*S*,13*S*,14*S*,17*S*)-2-(Adamantan-1-yl)-3,17-dimethoxy-13-methyl-7,8,9,11,12,13,14,15,16,17-decahydro-6*H* cyclopenta[*a*]phenanthrene (23):

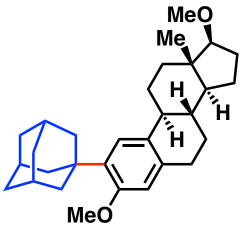

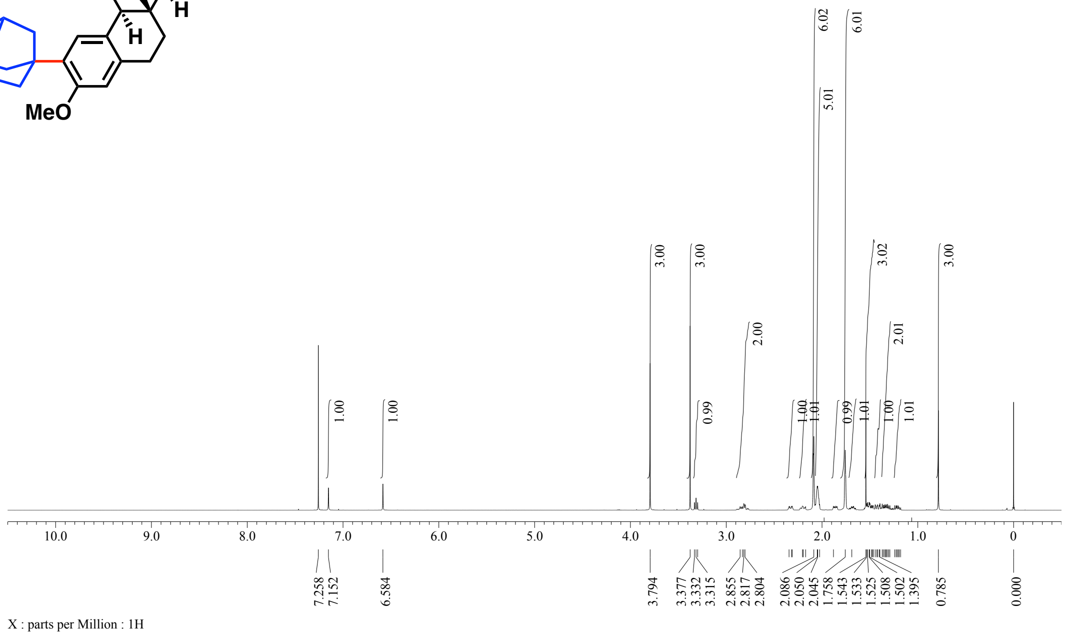

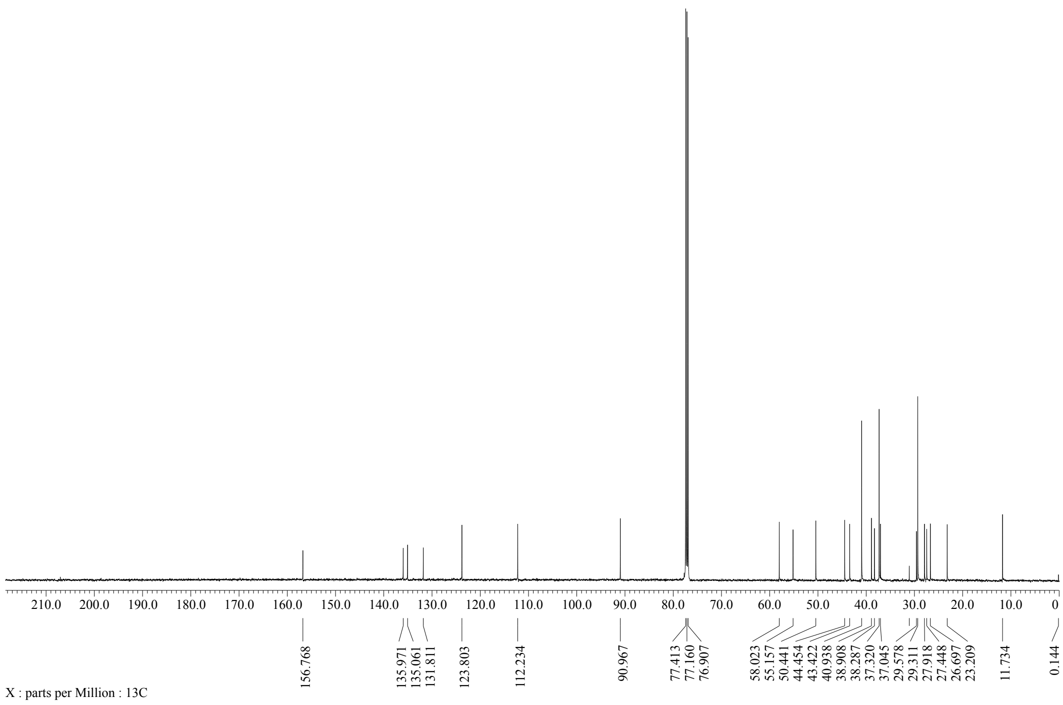

Methyl 4-(adamantan-2-yl)benzoate (25):

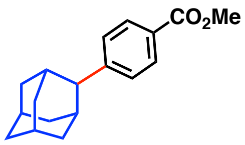

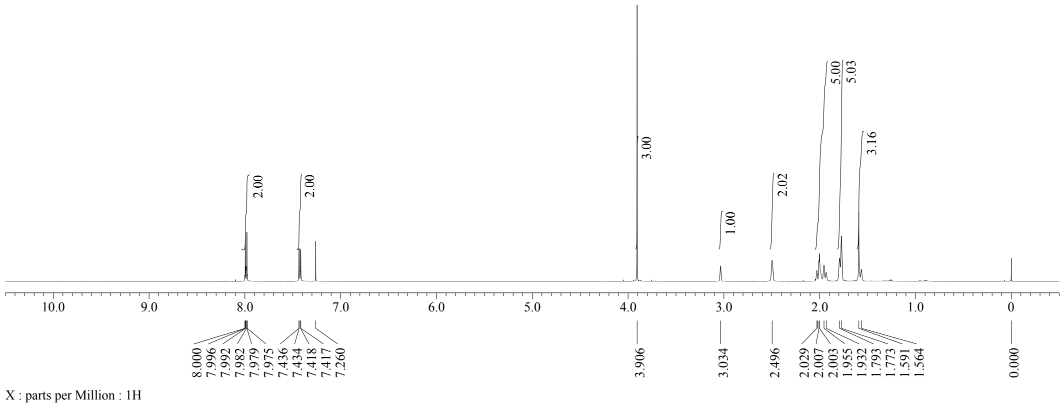

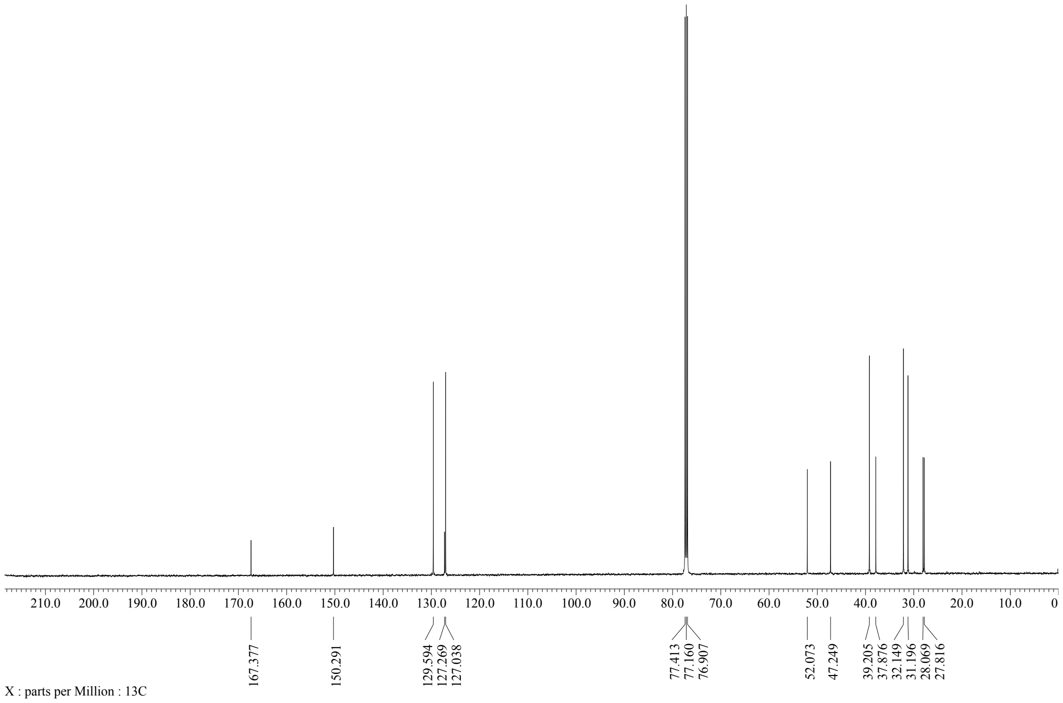

Methyl 2',4',6'-triisopropyl-[1,1'-biphenyl]-4-carboxylate (34):

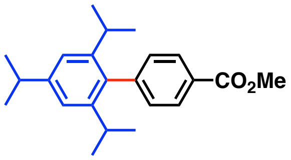

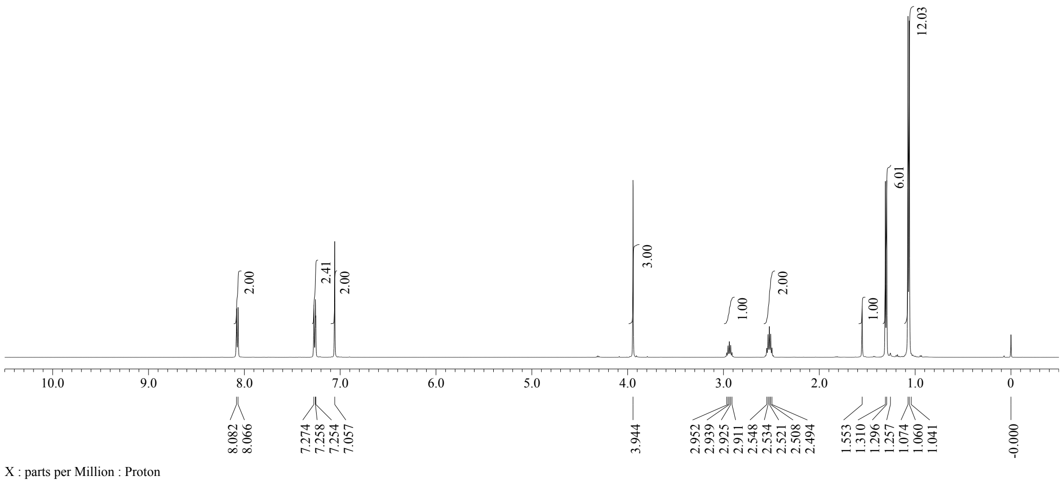

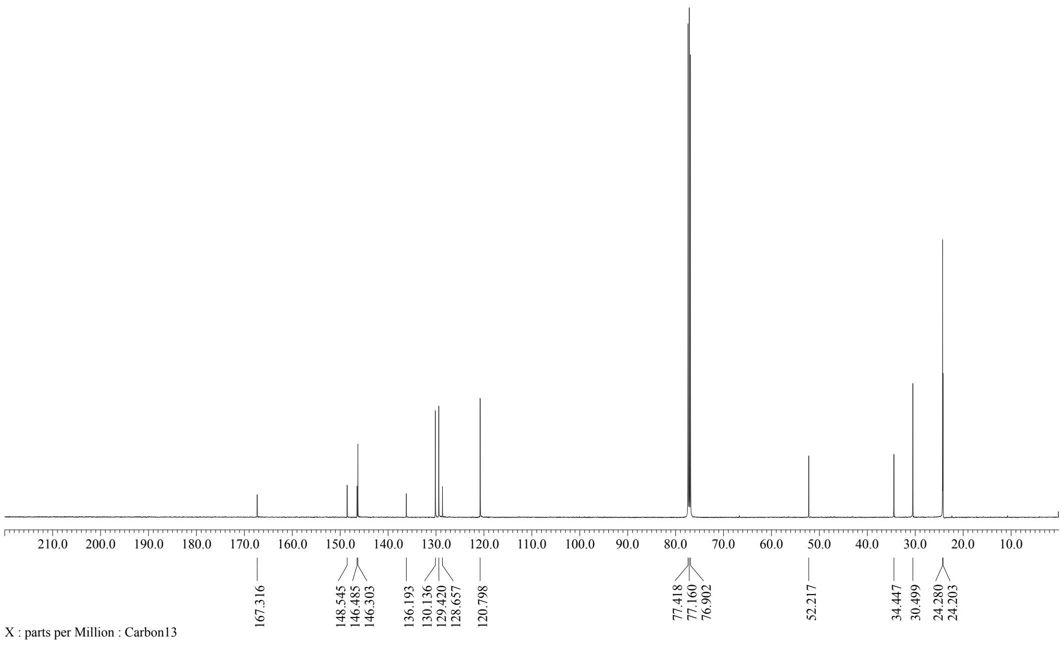

Methyl 2',4',6'-tri-*tert*-butyl-[1,1'-biphenyl]-4-carboxylate (35):

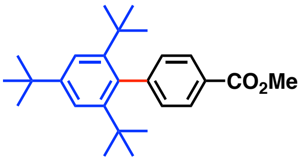

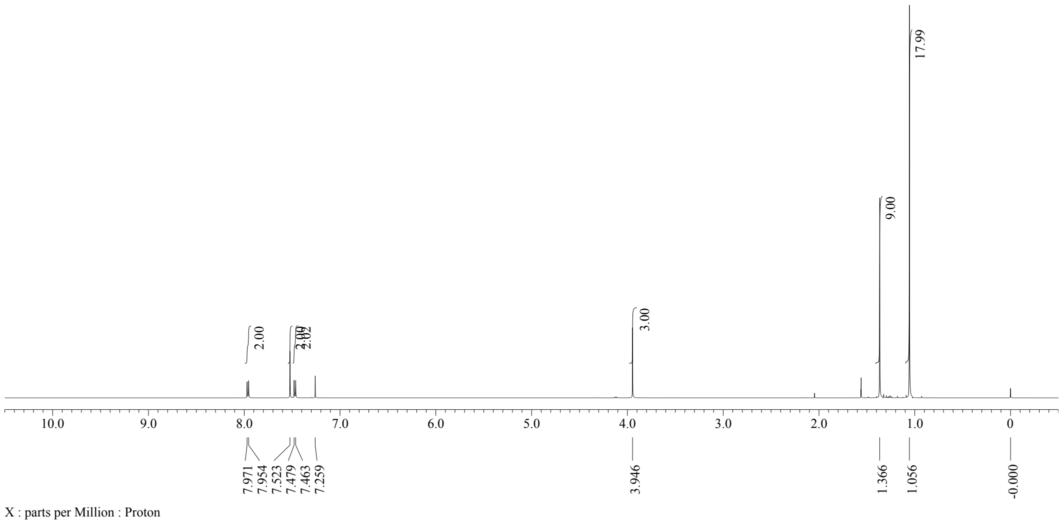

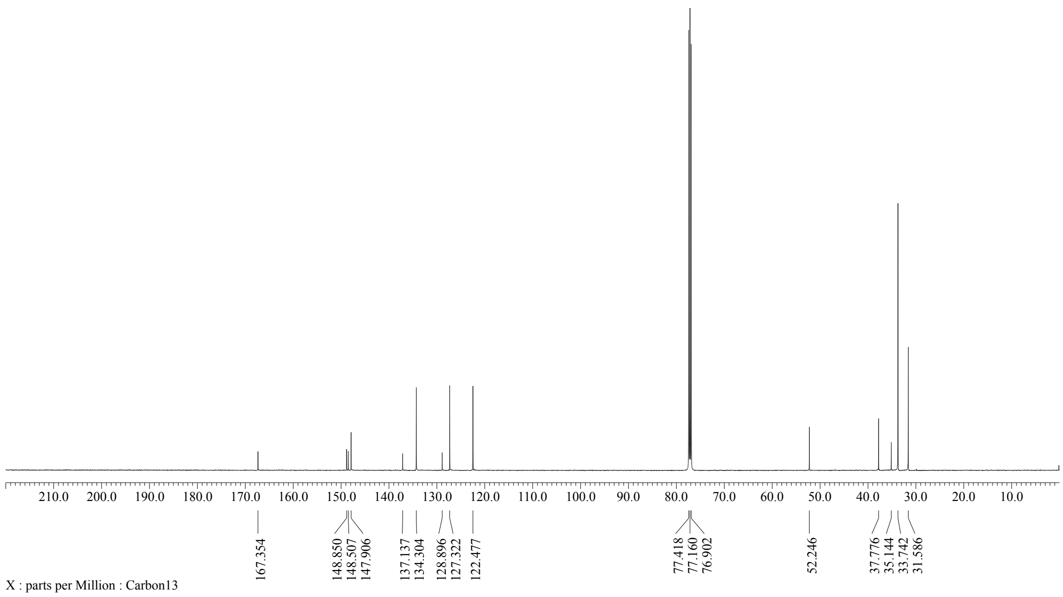

4-(*p*-Methylbenzoate)-1,1,7,7-tetraethyl-3,3,5,5-tetramethyl-1,2,3,5,6,7-hexahydro-*s*-indacene (36):

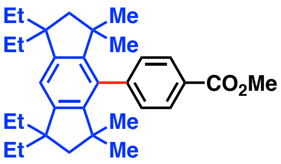

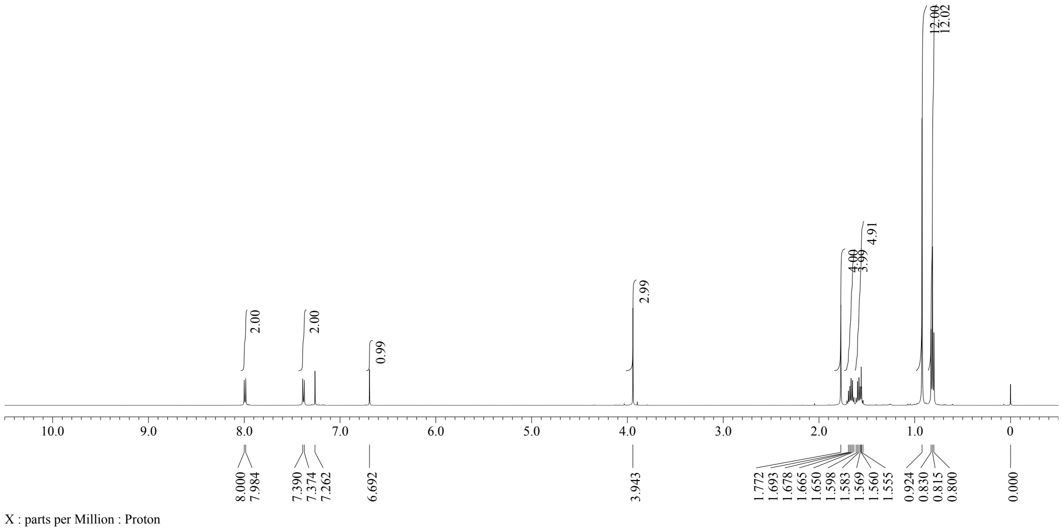

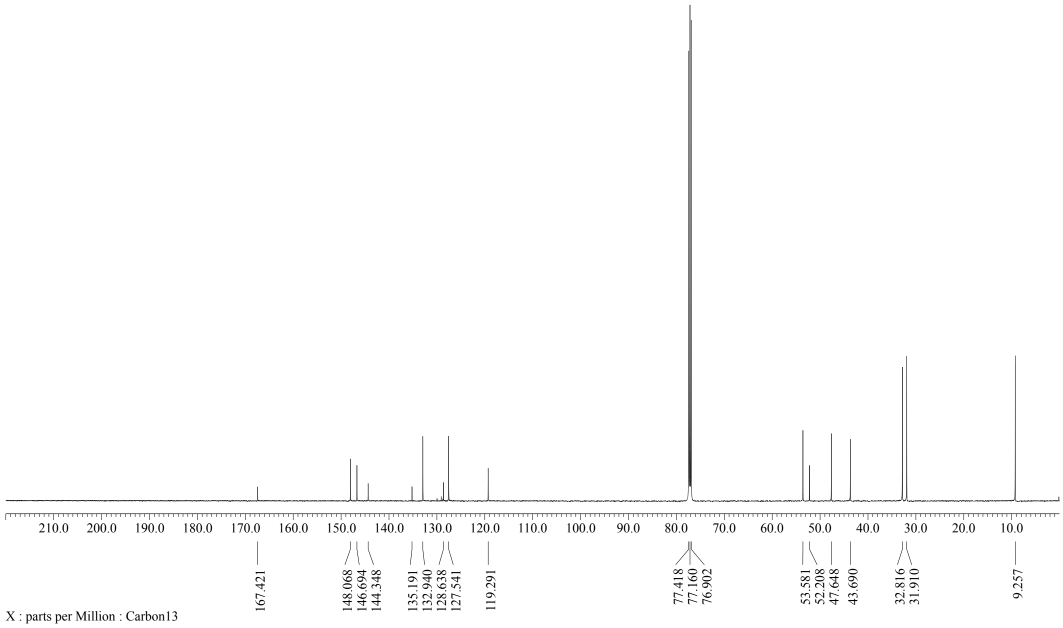

***N*,*N*-diisopropyl-4-mesitylcubane-1-carboxamide (37):**

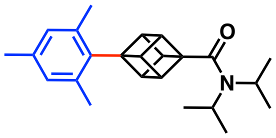

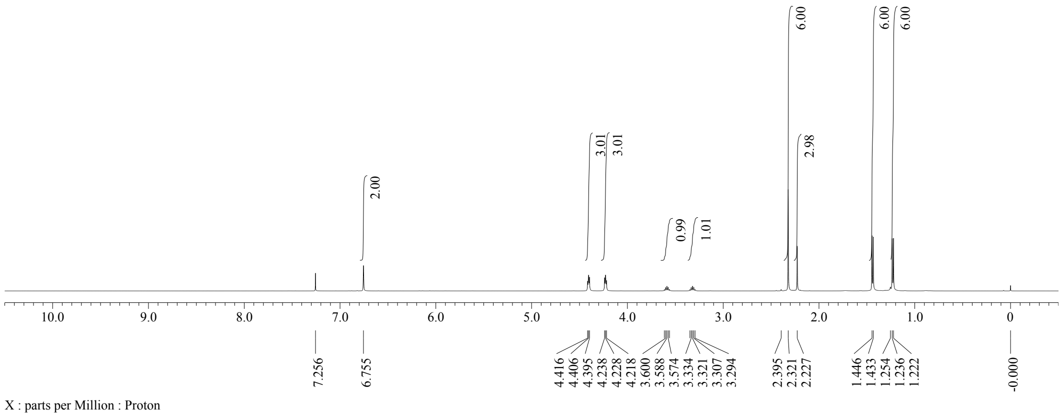

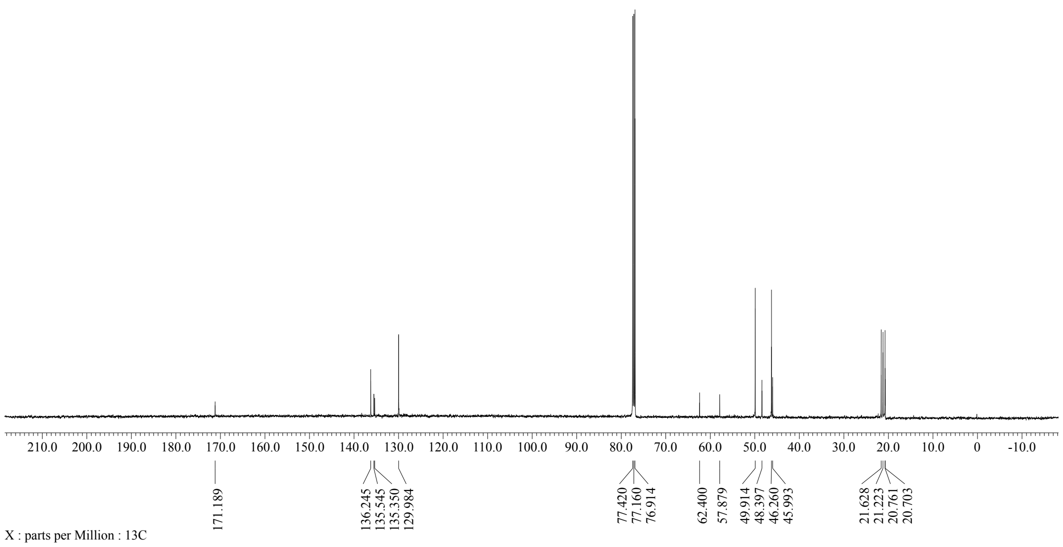

4-Iodo-*N,N*-diisopropylcubane-1-carboxamide (S3):

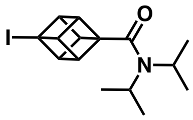

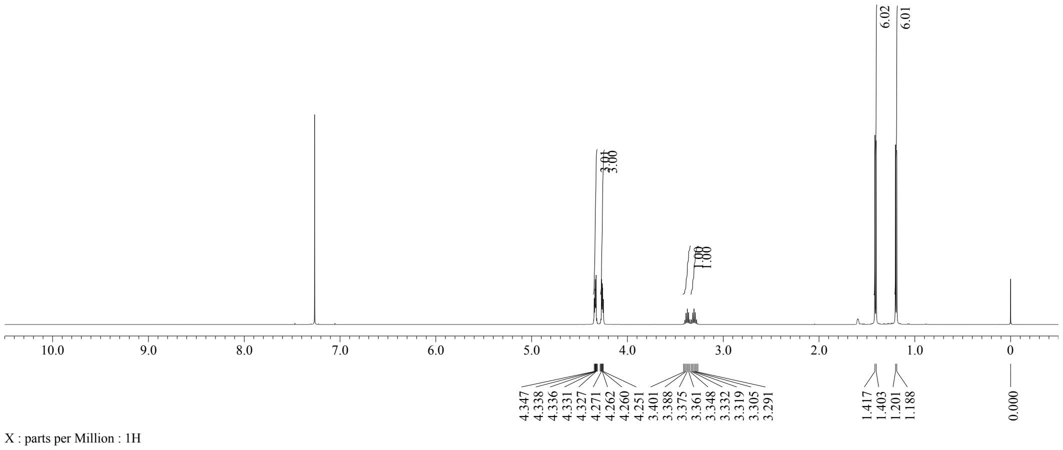

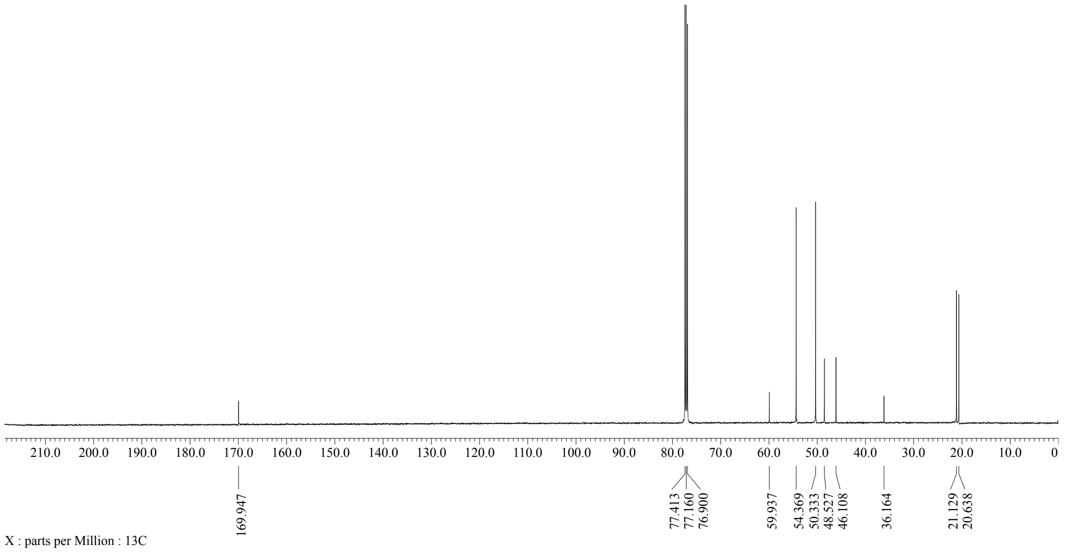

6. References

S1 (a) Y. Kawada and H. Iwamura, *J. Org. Chem*., 1981, **46**, 3357–3359; (b) G. Molle, P. Bauer and J. E. Dubois, *J. Org. Chem*., 1983, **48**, 2975–2981.

S2 (a) K. Fujimoto, H. Yorimitsu and A. Osuka, *Chem. Eur. J*., 2015, **21**, 11311–11314; (b) K. Kubota, K. Hayama, H. Iwamoto and H. Ito, *Angew. Chem. Int. Ed*., 2015, **54**, 8809–8813.

S3 T. Sperger, I. A. Sanhueza, I. Kalvet and F. Schoenebeck, *Chem. Rev*., 2015, **115**, 9532–9586.

S4 (a) A. D. Beche, *Phys. Rev.,* 1988, **A38**, 3098–3100; (b) A. D. Beche, *J. Chem. Phys.,* 1993, **98**, 1372–1377; (c) A. D. Beche, *J. Chem. Phys.,* 1993, **98**, 5648–5652; (d) C. Lee, W. Yang and R. G. Parr, *Phys. Rev.,* 1988, **B37**, 785–788.

S5 (a) Y. Zhao and D. G. Truhlar, *Theor. Chem. Acc.,* 2008, **120**, 215–241; (b) Y. Zhao and D. G. Truhlar, *Acc. Chem. Res.,* 2008, **41**, 157–167.

S6 J. D. Chai and M. Head-gordon, *J. Chem. Phys.,* 2009, **131**, 174105–174117.

S7 K. Morokuma, *J. Chem. Phys.,* 1971, **55**, 1236–1244.

S8 (a) *NBO 6.0.* E. D. Glendening, J. K. Badenhoop, A. E. Reed, J. E. Carpenter, J. A. Bohmann, C. M. Morales, C. R. Landis and F. Weinhold, Theoretical Chemistry Institute, University of Wisconsin, Madison (2013); (b) NBO6 Website: http://www.chem.wisc.edu/~nbo6; (c) E. D. Glendening, C. R. Landis and F. Weinhold, *J. Comp. Chem.,* 2013, **34**, 1429–1437.

S9 C. L. McMullin, N. Fey and J. N. Harvey, *Dalton Trans.,* 2014, **43**, 13545–13556.

S10 J. Louie and J. F. Hartwig, *J. Am. Chem. Soc.,* 1995, **117**, 11598–11599.

S11 P. E. Eaton, N. Nordari, J. Tanaktsidis and S. P. Updhyaya, *Synthesis*, 1995, 501.

S12 R. Priefer, P. G. Farrell and D. N. Harpp, *Synthesis*, 2002, **18**, 2671–2673.

S13 K. Nikitin, H. Müller-Bunz, Y. Ortin, J. Muldoon and M. J. McGlinchey, *J. Am. Chem. Soc.,* 2010, **132**, 17617–17622.

S14 I. I. Brunovlenskaya, A. A. Kolontsov and V. R. Skvarchenko, *Zhurnal Organicheskoi Khimii*, 1979, **15**, 1499–1502.

S15 X. Zhang and C. Yang, C. *Adv. Synth. Catal.,* 2015, **357**, 2721–2727.

S16 X. Wang, S. Wang, W. Xue and H. Gong, *J. Am. Chem. Soc.,* 2015, **137**, 11562–11565.

S17 Y.-X. Liu, D. Xue, J.-D. Wamg, C.-J. Zhao, Q.-Z. Zou, C. Wang and J. Xiao, *Synlett,* 2013, **24**, 507–513.

S18 J. C. Anderson, H. Namli and C. A. Roberts, *Tetrahedron,* 1997, **53**, 15123–15134.

S19 C.-L. Sun, H. Li, D.-G. Yu, M. Yu, X. Zhou, X.-Y. Lu, K. Huang, S.-F. Zheng, B.-J. Li and Z.-J. Shi, *Nat. Chem*., 2010, **2**, 1044–1049.

S20 C. E. Hartmann, S. P. Nolan and C. S. J. Cazin, *Organometallics*, 2009, **28**, 2915–2919.

S21 G. Cahiez, C. Chaboche, F. Mahuteau-Betzer and M. Ahr, *Org. Lett.,* 2005, **7**, 1943–1946.

S22 G.-Q. Li, Y. Yamamoto and N. Miyaura, *Synlett,* 2011, 1769–1773.

S23 T. Hasobe, S. Hattori, H. Kotani, K. Ohkubo, K. Hosomizu, H. Imahori, P. V. Kamat and S. Fukuzumi, *Org. Lett.,* 2004, **6**, 3103–3106.

S24 Gaussian 09, Revision D.01, M. J. Frisch, G. W. Trucks, H. B. Schlegel, G. E. Scuseria, M.A. Robb, J. R. Cheeseman, G. Scalmani, V. Barone, B. Mennucci, G. A. Petersson, H. Nakatsuji, M. Caricato, X. Li, H. P. Hratchian, A. F. Izmaylov, J. Bloino, G. Zheng, J. L. Sonnenberg, M. Hada, M. Ehara, K. Toyota, R. Fukuda, J. Hasegawa, M. Ishida, T. Nakajima, Y. Honda, O. Kitao, H. Nakai, T. Vreven, J. A. Montgomery, Jr., J. E. Peralta, F. Ogliaro, M. Bearpark, J. J. Heyd, E. Brothers, K. N. Kudin, V. N. Staroverov, R. Kobayashi, J. Normand, K. Raghavachari, A. Rendell, J. C. Burant, S. S. Iyengar, J. Tomasi, M. Cossi, N. Rega, J. M. Millam, M. Klene, J. E. Knox, J. B. Cross, V. Bakken, C. Adamo, J. Jaramillo, R. Gomperts, R. E. Stratmann, O. Yazyev, A. J. Austin, R. Cammi, C. Pomelli, J. W. Ochterski, R. L. Martin, K. Morokuma, V. G. Zakrzewski, G. A. Voth, P. Salvador, J. J. Dannenberg, S. Dapprich, A. D. Daniels, Ö. Farkas, J. B. Foresman, J. V. Ortiz, J. Cioslowski and D. J. Fox, Gaussian, Inc., Wallingford CT, 2009.

S25 (a) S. Maeda, Y. Osada, K. Morokuma and K. Ohno, *GRRM 11, Version 11.03* (2012); (b) S. Maeda, K. Ohno and K. Morokuma, *Phys. Chem. Chem. Phys.,* 2013, **15**, 3683–3701; (c) K. Ohno and S. Maeda, *Chem. Phys. Lett.,* 2004, **384**, 277–282; (d) S. Maeda and K. Ohno, *J. Phys. Chem. A*, 2005, **109**, 5742–5753; (e) K. Ohno and S. Maeda, *J. Phys. Chem. A*, 2006, **110**, 8933–8941.

S26 (a) P. Fuentealba, H. Preuss, H. Stoll and L. V. Szentpaly, *Chem. Phys. Lett.,* 1982, **89**, 418–422; (b) L. V. Szentpaly, P. Fuentealba, H. Preuss and H. Stoll, *Chem. Phys. Lett.,* 1982, **93**, 555–559.

S27 M. J. Frisch, J. A. Pople and J. S. Binkley, *J. Chem. Phys.,* 1984, **80**, 3265–3269.

S28 (a) K. Fukui, *Acc. Chem. Res.,* 1981, **14**, 363–368; (b) K. Ishida, K. Morokuma and A. Komornicki, *J. Chem. Phys.,* 1977, **66**, 2153–2156; (c) C. Gonzalez and H. B. Schlegel, *J. Chem. Phys.,* 1989, **90**, 2154–2161; (d) H. B. Schlegel and C. Gonzalez, *J. Phys. Chem.,* 1990, **94**, 5523–5527.

S29 P. J. Hay and W. R. Wadt, *J. Chem. Phys.,* 1985, **82**, 299–310.
